# Molecular glue that stabilizes the LRPPRC−*MET*-G4 interaction complex to drive *MET* downregulation

**DOI:** 10.1038/s41467-026-73806-6

**Published:** 2026-06-04

**Authors:** Yi-Pu Li, Ying Zheng, Yu-Shuang Liu, Li-Fang Zhang, Xin-Yuan Chen, Si-Han He, Juan-Nan Chen, Rong-Shuang Cheng, Yu Wang, Ying-Ying Wang, Li Tang, Ke-Wei Zheng, Sheng-Tao Xu, Jin-Lei Bian, Hao Zhang, Kai-Bo Wang, Ling-Yi Kong

**Affiliations:** 1 State Key Laboratory of Natural Medicines, China Pharmaceutical University, Nanjing, China; 2 Basic Medical Research Innovation Center for Anti-Cancer Drugs (Ministry of Education), China Pharmaceutical University, Nanjing, China; 3 Jiangsu Key Laboratory of Bioactive Natural Product Research, China Pharmaceutical University, Nanjing, China; 4 School of Biomedical Sciences, Hunan University, Changsha, Hunan China

**Keywords:** Gene regulation, Cancer therapy, Gene silencing

## Abstract

Targeting the MET oncoprotein is an effective strategy in precision cancer therapy, whereas its clinical efficacy varies dramatically across tumor types. Herein, we explore an alternative approach to downregulate *MET* at the transcriptional level. We identify a cis-regulatory element in the *MET* proximal promoter that forms a stable parallel G-quadruplex (*MET*-G4). We determine the high-resolution NMR solution structure of this *MET*-G4 and demonstrate that *MET*-G4 recruits LRPPRC to promote *MET* transcription, uncovering a previously unrecognized epigenetic mechanism that drives *MET* overexpression. We further characterize LRPPRC’s G4-binding domain and its structural basis for *MET*-G4 recognition. Through screening an in-house natural product library, we identify nitidine (NIT) as a *MET*-G4 stabilizer that acts as a molecular glue, strengthening the LRPPRC–*MET*-G4 interaction and inducing the formation of a stable LRPPRC−NIT−*MET*-G4 ternary complex. This complex likely alters the structure and function of LRPPRC, thereby downregulating *MET* expression and exerting the well-known anticancer effects of nitidine. Moreover, comprehensive in vitro and in vivo experiments demonstrate that nitidine significantly inhibits tumor progression through an LRPPRC−*MET-*G4-dependent mechanism. Collectively, our study suggests an epigenetic regulatory mechanism involving LRPPRC−*MET-*G4-mediated *MET* upregulation and provides a promising therapeutic strategy for *MET*-driven tumors using molecular glues that target the LRPPRC−*MET*-G4 interface.

## Abstract (web summary)

*MET* upregulation drives many human cancers. Herein, the authors identify a G-quadruplex in the *MET* promoter that recruits LRPPRC to boost *MET* transcription, and show that nitidine acts as a molecular glue to stabilize the LRPPRC–*MET*-G4 complex, suppressing *MET* expression and inhibiting tumor growth.

## Introduction

The *MET* oncogene, located on chromosome 7q21-q31, encodes the MET receptor tyrosine kinase, also known as c-MET^1^. Upon binding to its ligand, hepatocyte growth factor (HGF), the *MET* signaling pathway is activated, triggering downstream signaling cascades, including RAS-RAF-MAPK and PI3K-AKT/mTOR^2^. Normal *MET* signaling is crucial in embryogenesis, tissue regeneration, and wound healing^3^. However, aberrant and deregulated *MET* signaling, characterized by protein overexpression and genetic alterations such as amplification, mutation, and fusion, has been implicated in various malignancies, including lung cancers, hepatocellular carcinoma (HCC), gastric cancers, and colorectal cancers^4^. Notably, *MET* amplification is a well-documented resistance mechanism that commonly emerges in response to targeted anticancer therapies. This is particularly evident when tumors initially driven by other oncogenes, such as mutant *EGFR*, subsequently develop a secondary dependence on the *MET* pathway^5^. Consequently, extensive research has been conducted on targeting MET as a mechanism of action to overcome resistance to EGFR inhibitors^6,7^. Moreover, genetic alterations in *MET* that hyperactivate the RAS signaling pathway have been identified as a key mechanism of resistance to RAS inhibitors^8^. Elevated *MET* transcription is also associated with the activation of hypoxia-inducible factor (HIF) and inflammatory mediators^9^. Dysregulated *MET* signaling plays critical roles in initiating and maintaining multiple pathogenic processes, including cell proliferation, survival, resistance to apoptosis, angiogenesis, and metastasis, which are closely associated with poor prognosis in human malignancies^4,10^. Therefore, the therapeutic and prognostic benefits of targeting MET have garnered significant interest in cancer therapy.

Several MET-targeted tyrosine kinase inhibitors (TKIs), such as capmatinib^11^, tepotinib^12^, and savolitinib^13^, have been approved for the treatment of non-small-cell lung cancers (NSCLC) with *MET* exon 14 skipping mutations. Additionally, two multi-targeting TKIs, crizotinib and cabozantinib, have been approved for NSCLC patients and thyroid tumors that are not exclusively associated with MET activation^14,15^. However, the clinical benefits of these MET-targeted TKIs are limited, especially in patients with *MET* gene amplification and protein overexpression^16^. Moreover, their efficacy across various tumor types is modest, with only limited therapeutic response observed in HCC patients^17^. Currently, cabozantinib, a multi-targeted TKI that includes MET among its targets, is the only FDA-approved drug for patients with MET-dysregulated HCC and has been used as a second-line therapy for advanced and progressing cases since 2019^18,19^. Additionally, several MET-targeting antibodies, antibody-drug conjugates (ADCs), and chimeric antigen receptor T (CAR-T) cell therapies with diverse mechanisms are currently undergoing pre-clinical validation or clinical testing for NSCLC and HCC patients^20,21^. Whereas strategies that solely block the MET/HGF interaction with antibodies have demonstrated limited clinical efficacy, underscoring the challenges associated with developing effective MET-targeted antibody therapies^22^. The effectiveness of MET-directed therapeutics hinges on the accurate identification of *MET* as the primary oncogenic driver, particularly in MET-driven malignancies such as certain subtypes of lung cancers^23^. However, the absence of reliable biomarkers to identify responsive patients complicates patient selection. Therefore, a deeper understanding of the dysregulated *MET* signaling pathway is crucial for elucidating cancer-associated regulatory mechanisms and developing MET-targeted therapies.

G-quadruplexes (G4s) are non-canonical secondary structures that spontaneously form in single-stranded guanine-rich (G-rich) nucleic acid sequences in DNA or RNA through Hoogsteen hydrogen bonding and are stabilized by monovalent cations such as potassium (K⁺) or sodium (Na⁺)^24^. Accumulated at specific genome loci, G4s function as epigenetic regulators implicated in gene replication, transcription, and genome stability^25^. G4s have been shown to play a significant role in the development and progression of cancers. They act as binding hubs for transcription factors (TFs) or proteins, thereby contributing to transcriptional machinery in the human chromatin landscape^26^. Several G4-binding proteins, including DHX36^27^, hnRNP A1^28^, PARP1^29^, Rif1^30^, SLIRP^31^, and PRC2^32^, have been identified based on their functional roles in G4-mediated gene regulation. Given their unique structural features that enable specific nucleic acid targeting, G4s have emerged as attractive therapeutic targets for anticancer drug development^33^. Notably, targeting oncogene promoter G4 structures has offered alternative strategies to address the challenge of these “undruggable” and drug-resistant proteins, such as MYC, KRAS, and EGFR^34^. Strikingly, potential G4-forming sequences were identified within the proximal promoter region of the *MET* oncogene through G4-ChIP-seq experiments^35^. A G-rich sequence known as Pu24, located in the *MET* oncogene proximal promoter region from position −80 to −57 upstream of the transcription start site, is believed to form the major *MET* promoter G4 (*MET*-G4) involved in *MET* oncogene regulation^36^.

In this work, we determine the NMR solution structure of the major *MET*-G4 using a Pu25m1T DNA derived from the *MET* proximal promoter region. The *MET*-G4 adopts a three-tetrad core, parallel-stranded G4 with a unique 5-nucleotide third loop. Through G4-pull-down/MS/MS analysis and RNA interference (RNAi) experiments, we identify LRPPRC as a critical cellular protein that interacts with the *MET*-G4 to promote *MET* overexpression. Utilizing purified LRPPRC fragments, we demonstrate that the 1025-1394 domain of LRPPRC is responsible for *MET*-G4 binding. Subsequently, we construct the binding model and identify key interfacial residues of the LRPPRC−*MET*-G4 interaction complex by integrating AlphaFold prediction, molecular docking, and Amber molecular dynamics (MD) simulations. With the established LRPPRC−*MET*-G4 model system, we screen an in-house natural product library and discover that nitidine alkaloid is a strong *MET*-G4 stabilizer. Notably, nitidine significantly lowers *MET* expression levels via an *MET*-G4-dependent mechanism. Moreover, we demonstrate that nitidine functions as a molecular glue to promote the formation of a stable LRPPRC−NIT−*MET*-G4 ternary complex. Chromatin immunoprecipitation (ChIP) experiments confirm the enrichment of LRPPRC at the *MET*-G4-associated *MET* proximal promoter sequence, with nitidine enhancing *MET*-G4 stabilization and LRPPRC occupancy in human cancer cells. Additionally, both in vitro and in vivo experiments demonstrate that nitidine inhibits the proliferation of liver and lung cancer cells, induces apoptosis and G2/M phase arrest, and suppresses tumor progression without causing obvious toxicity. Collectively, these findings suggest an epigenetic mechanism driving *MET* upregulation and establish a promising anticancer strategy by targeting the LRPPRC−*MET*-G4 interaction complex for MET-driven tumors.

## Results

### Formation of a parallel G-quadruplex in the *MET* oncogene proximal promoter sequence

**Fig. 1:**
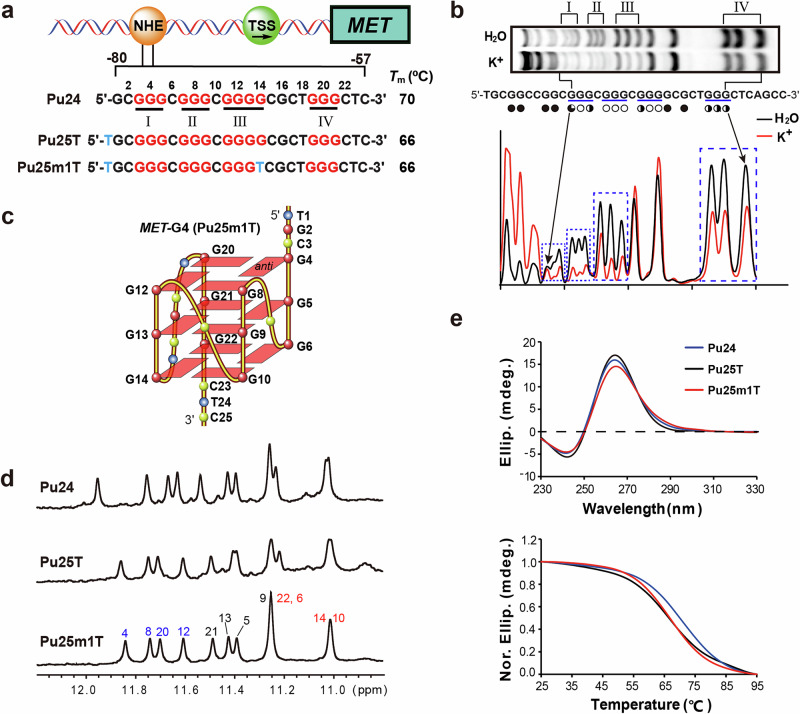
Formation of a Parallel G-quadruplex in the *MET* Oncogene Proximal Promoter Region. **a** The schematic representation of the human *MET* oncogene promoter highlights the G4-forming region. The DNA sequences involved in the formation of major *MET*-G4 and its modifications are shown. The G-runs with three or four continuous guanines are underlined and numbered. The guanines involved in the major *MET*-G4 formation and mutations are colored in red and blue, respectively. The ECD melting temperatures of DNA sequences in 50 mM K^+^-containing solution are provided. **b** DMS footprinting of the wild-type Pu36 DNA showing the guanines involved in G-tetrad formation of *MET-*G4. The experiment was repeated three times independently with similar results; a representative image is shown. **c** Folding topology of *MET-*G4 with the Pu25m1T DNA sequence. **d**, **e** ^1^H NMR spectra, ECD spectra, and ECD thermal melting curves of wild-type and mutant *MET* gene promoter sequences. Imino proton signals at the 5′-end (blue), middle (black), and 3′-end (red) are marked. Conditions: 20 and 150 μM DNA, pH 7.0, 50 mM K^+^. Source data are provided as a Source Data file.

The G-rich sequence, located from position –80 to –57 upstream of the transcriptional start site (TSS) in the proximal promoter region of the *MET* oncogene, has a high potential to form G4 structures, as supported by G4-specific ChIP-seq data and previous studies (Fig. 1a)^35,37^. Subsequently, we performed a DMS footprinting assay using a wild-type Pu36 DNA sequence to identify which guanines (Gs) are involved in G-tetrad formation (Fig. 1b). Guanines involved in G-tetrad formation are Hoogsteen hydrogen-bonded, making them resistant to DMS methylation and subsequent piperidine cleavage during the DMS footprinting experiments, a well-established technique for assessing G4 formation under near-physiological conditions^38^. As shown in Fig. 1b, significant protection of 12 Gs across G-runs I to Ⅳ was observed, suggesting the formation of a canonical three-layered G4 structure of the major *MET*-G4 (Fig. 1c).

To gain structural insight into the major *MET*-G4, we aimed to determine its NMR solution structure. Initially, we chose the truncated wild-type Pu24 DNA for NMR analysis because it contains all the necessary Gs required for the formation of the major *MET*-G4. The ^1^H NMR spectrum of the wild-type Pu24 DNA in K^+^-containing solution displayed 12 well-defined imino proton peaks, confirming the formation of a three-layered G4 structure. Whereas minor species were observed, as evidenced by small satellite-like peaks (Fig. 1d). To improve spectral quality for high-resolution NMR structural determination, we introduced an additional thymine residue at the 5′-end, generating Pu25T DNA. However, the NMR spectral quality of Pu25T was either comparable to or inferior to that of Pu24 DNA (Fig. 1a). Since G14 is not involved in the formation of the major *MET*-G4 (Fig. 1c), we introduced a G14-to-T14 mutation and prepared Pu25m1T DNA. This modification substantially improved the NMR spectral quality by eliminating the small satellite-like peaks (Fig. 1d). Meanwhile, the pattern of imino proton peaks in Pu25m1T remained consistent with that of Pu24 DNA (Fig. 1d), suggesting the formation of an identical G4 topology. Furthermore, ECD experiments demonstrated that Pu24, Pu25T, and Pu25m1T DNAs all adopt parallel G4 topologies, as evidenced by a conspicuous maximum ellipticity at 264 nm and a minimum at 242 nm (Fig. 1e and Supplementary Table 1). Additionally, Pu24, Pu25T, and Pu25m1T exhibited similar high *T*_m_ values (over 66 °C) in a 50 mM K^+^-containing solution, suggesting a strong potential for *MET*-G4 formation in cells for gene regulation (Fig. 1a). Taken together, we selected Pu25m1T for further structural determination.

### NMR solution structure determination of the major *MET* proximal promoter G-quadruplex

**Fig. 2:**
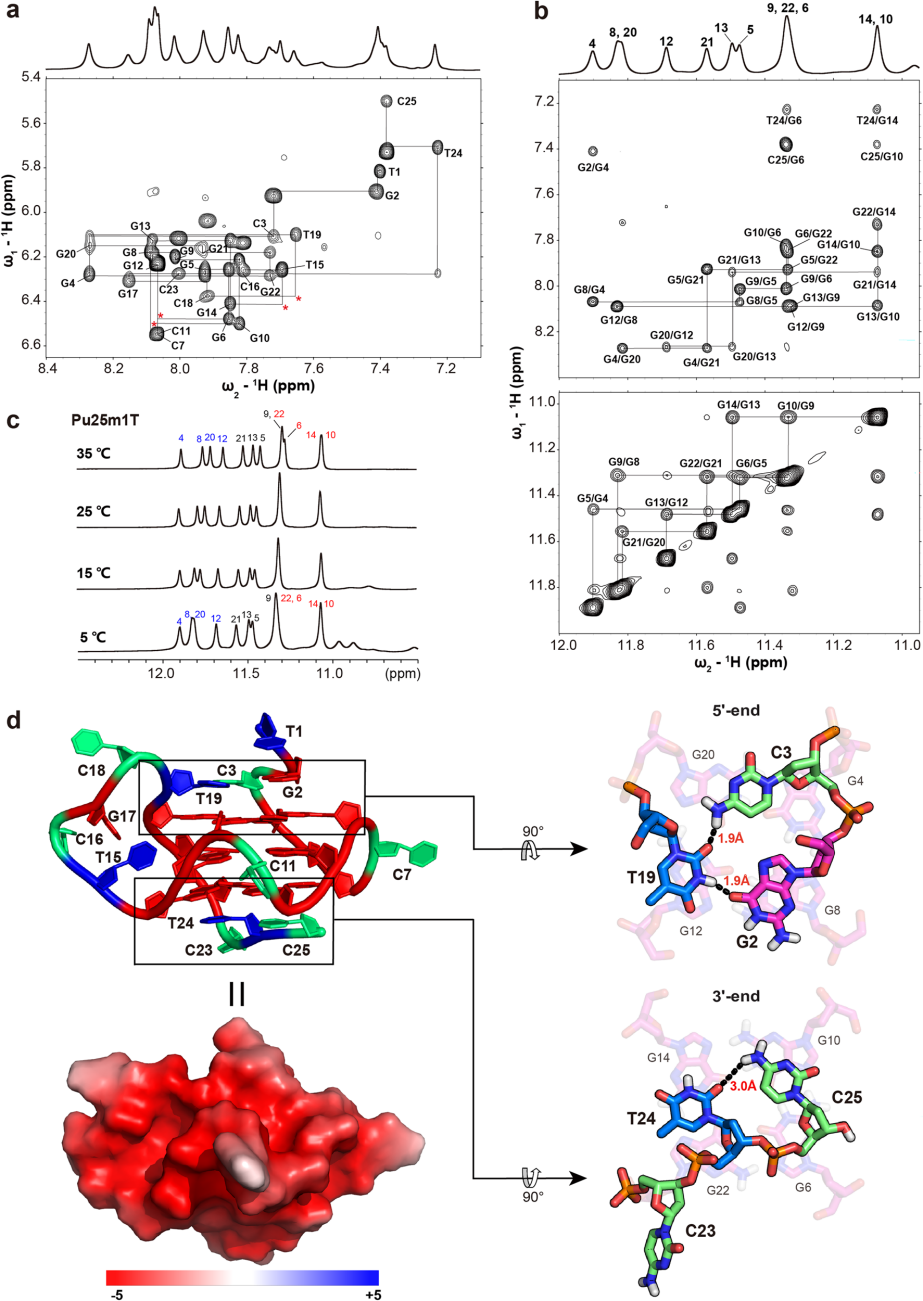
High-resolution NMR Solution Structure Determination of the *MET* Proximal Promoter G-quadruplex. **a**, **b**, H1′-H6/H8 region, H1-H1 and H1-H8 regions derived from 2D-NOESY spectra of Pu25m1T DNA with sequential assignment pathway. Missing connectivities are denoted with red asterisks. **c** ^1^H NMR spectra of Pu25m1T DNA with assignment at varying temperatures. The G-tetrad imino proton signals at the 5′-end (blue), middle (black), and 3′-end (red) are indicated. Conditions: 1.51 mM DNA, pH 7.0, 25 mM K^+^. **d** Cartoon representation and electrostatic potential energy map of a refined *MET-*G4 with partial residue numbering (PDB ID: 9JI9). 5′-end and 3′-end top views of *MET-*G4 are shown. Magenta, guanine; green, cytosine; blue, thymine. Source data are provided as a Source Data file.

To determine the solution structure of major *MET*-G4, we collected a set of 1D and 2D NMR spectra for Pu25m1T DNA, including NOESY, HSQC, and DQF-COSY at varying temperatures and mixing times (Fig. 2a–c, Supplementary Fig. 1, Supplementary Fig. 2, and Supplementary Fig. 3). Following well-established protocols, we successfully assigned proton peaks corresponding to imino, aromatic, and sugar resonances of Pu25m1T DNA^39^. The H1′-H6/H8 NOE cross-peaks exhibited moderate intensities, and the C6/C8 chemical signals were downfield-shifted, indicating that all residues adopted anti-glycosidic torsion angles characteristic of a parallel-stranded G4 structure (Fig. 2a and Supplementary Tables 2–5)^39^. The core of three G-tetrads was determined as G4-G8-G12-G20, G5-G9-G13-G21, and G6-G10-G14-G22 based on H1-H1 and H1-H8 NOE cross-peaks (Fig. 2b and Supplementary Fig. 4a). Additionally, solvent exchange experiments confirmed the folding topology of *MET*-G4, as the guanine imino protons in the central G-tetrad (G5, G9, G13, and G21) exhibited greater protection compared to those in the outer G-tetrads (Supplementary Fig. 4b). The high-resolution NMR solution structures of major *MET*-G4 were then determined using molecular dynamics (MD) simulations, incorporating distance information obtained from NOESY spectra (Table 1). A total of 522 NOE-derived distances, 48 H-bond restraints, and 25 torsion-angle restraints guided the MD process. The resulting fifteen lowest-energy structures exhibited good convergence (Supplementary Fig. 4c), with a heavy atom root-mean-square deviation (RMSD) value of 0.38 ± 0.14 Å for the G-tetrad core and 0.53 ± 0.17 Å for all residues, respectively.

**Table 1:** NMR restraints and structural statistics for the *MET*-G4

	*MET*-G4
NOE-Based Distance Restraints	
Total	688
Intra-residue	357
Inter-residue	
Sequential	221
Long-range	110
Other Restraints	
Hydrogen bonds restraints	48
Torsion angles restraints	25
G-tetrad planarity restraints	48
Structural Statistics	
Pairwise heavy atom RMSD (Å)	
G-tetrad core	0.38 ± 0.14
All residues	0.53 ± 0.17
Restraint violations (Å)	
Max. NOE restraint violation	0.11
Mean NOE restraint violation	0.001 ± 0.008

The obtained NMR solution structure of the major *MET*-G4 confirmed its parallel G4 topology (Fig. 2d). The three-G-tetrad core of *MET*-G4 is sandwiched between the G2·C3·T19 triad at the 5′-end and the T24·T25 pair at the 3′-end (Fig. 2d and Supplementary Fig. 4d, e). The G2·C3·T19 triad consists of two flanking residues, G2 and G3, along with a loop-recruiting residue, T19. This triad is stabilized by two potential hydrogen bonds and capped by T1 (Fig. 2d and Supplementary Fig. 4f). In contrast, the T24·T25 pair consists solely of flanking residues and exhibits weaker hydrogen bonding compared to the G2·C3·T19 triad. Notably, residue C23 bridges the G-tetrad core and the 3′-terminal T24·C25 pair (Supplementary Table 5), forming a unique capping conformation not previously reported. Furthermore, the remaining four-residue loop fragment T15-C16-G17-C18 adopts a well-defined conformation, enhancing the overall compactness of the *MET*-G4 and minimizing solvent exposure (Fig. 2d and Table 1). The stacking interaction between C16 and T17 arises from strong π-π interactions, while T15 resides within the groove formed by residues C16-T17 and the *MET*-G4 core. Additionally, C18 protrudes from the main scaffold of *MET*-G4, exhibiting relative flexibility and solvent exposure (Fig. 2d). Overall, the solution structure of major *MET*-G4 is well-characterized, featuring distinct loop and 3′-end capping conformations that may facilitate specific protein interactions for diverse biological functions.

### *MET*-G4 recruits the cellular LRPPRC protein for *MET* upregulation

**Fig. 3:**
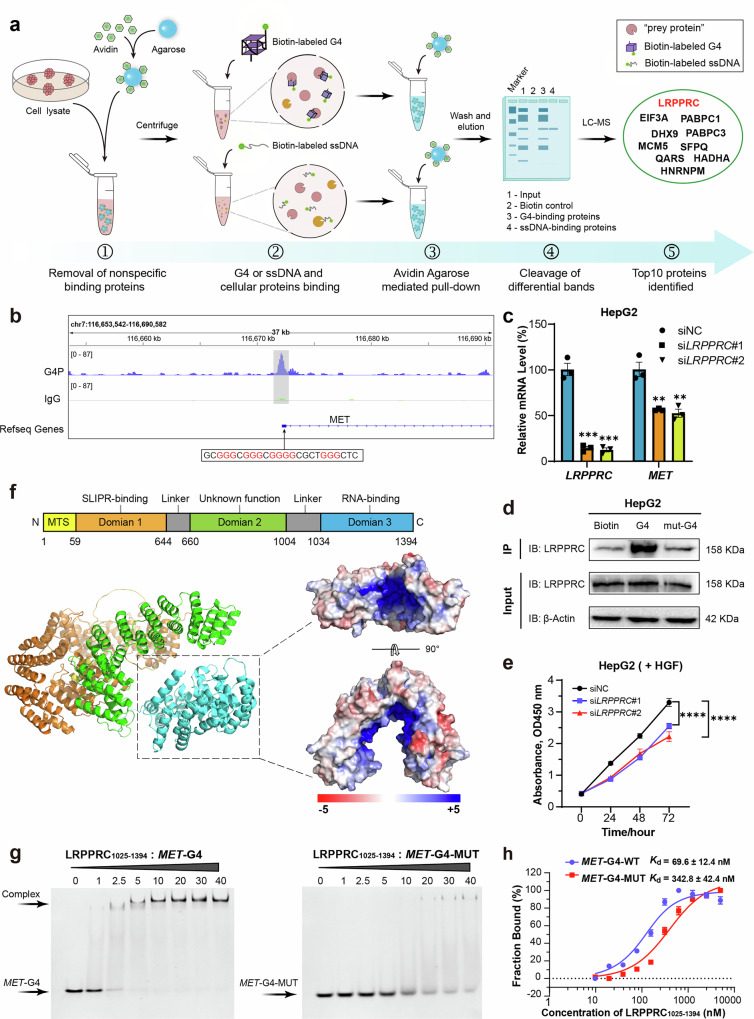
Cellular LRPPRC Protein is Recruited by *MET*-G4. **a** Schematic diagram of G4-mediated affinity pull-down assay. Created in BioRender. Li, Y. (2026) https://BioRender.com/vydkuwb. **b** Genomic CUT&Tag binding profiles (IGV tracks) for G4P in HepG2 cells. **c** *MET* mRNA expression levels in HepG2 cells with or without LRPPRC knockdown. Data means ± S.E.M. from three independent experiments. siNC vs. si*LRPPRC*#1, ***p = 0.0003, **p = 0.005; siNC vs. si*LRPPRC*#2, ***p = 0.0002, **p = 0.006. Statistical analysis was performed using a two-tailed *t*-test. **d** Pull-down combined with western blotting assay was carried out to examine the interaction between LRPPRC and *MET*-G4 or mutant-G4 in HepG2 cells. The experiment was repeated three times independently with similar results; a representative image is shown. **e** Cell proliferation was assessed using the CCK-8 assay in HepG2 cells with or without LRPPRC knockdown at the indicated time points in the presence of 40 ng/mL HGF. Data are presented as mean ± SEM. *n* = 3, Two-way ANOVA. ****p = 0.000003 (siNC vs. si*LRPPRC*#1); ****p < 0.000001 (siNC vs. si*LRPPRC*#2). **f** Domain architecture of full-length LRPPRC and its AlphaFold 3.0 predicted structure. The electrostatic potential surface views of the C-terminal region of LRPPRC (1025-1394) are shown. **g** EMSA gel results of LRPPRC_1025-1394_ binding to FAM-labeled Pu25m1T (*MET*-G4) or Pu25m1T-MUT (*MET*-G4-MUT) DNA. Condition: 0.1 μM FAM-labeled DNA, pH 8.0, 100 mM K^+^. The experiment was repeated three times independently with similar results; a representative image is shown. **h** The fraction bound activity (the normalized fluorescence difference between the LRPPRC bound-state and unbound-state of FAM-labeled DNA from MST measurements) is plotted against the concentration of LRPPRC_1025-1394_ protein. A curve fit yields the dissociation constant (*K*_d_) value of each DNA. Data are presented as mean ± SD. *n* = 3 independent experiments. For some points, the error bars are shorter than the height of the symbol and are not shown. Source data are provided as a Source Data file.

We hypothesized that the *MET*-G4 structure could serve as a unique binding site for specific transcription factors, thereby driving *MET* oncogene overexpression. To this end, we employed an integrated approach, combining G4-based affinity pull-down with mass spectrometry (MS) analysis, in HepG2 cells (Fig. 3a)^40^. We initially validated HepG2 cells as a suitable model: western blotting confirmed moderate basal MET protein expression and siRNA-mediated *MET* knockdown significantly inhibited cell proliferation, especially under HGF stimulation, demonstrating functional *MET* dependency (Supplementary Fig. 5a–e). Complementing this, G4-CUT&Tag experiments in HepG2 cells revealed a significant enrichment of G4 signals at the *MET* proximal promoter region (Fig. 3b)^37^. Guided by these findings, a biotin-labeled *MET*-G4-forming sequence was used as bait to capture proteins binding to *MET*-G4 in HepG2 cells. In contrast, a biotin-labeled *MET*-G4-MUT sequence served as a control bait to exclude non-specific DNA-binding proteins. The formation of *MET*-G4 and non-G4 structures was confirmed by ECD spectra (Supplementary Fig. 6a). After incubation with HepG2 cell lysate, the biotin-labeled sequences were immobilized on avidin agarose beads for protein enrichment. Subsequently, the enriched proteins were separated by SDS-PAGE gel electrophoresis and subjected to LC-MS/MS analysis (Supplementary Fig. 6b). Based on MS/MS count values, well-known G4-binding proteins such as DHX9^41^, DDX3X^42^, and DDX21^43^ were enriched, validating our approach for identifying G4-binding proteins. For the top 10 functional protein candidates, we performed siRNA-mediated knockdown experiments and analyzed their effects on *MET* expression. Notably, except for DHX9, the G4-binding activities of these 10 proteins remain largely uncharacterized. The RT-qPCR analysis revealed that the knockdown of LRPPRC, DHX9, EIF3A, HADHA, and SFPQ reduced *MET* mRNA levels in HepG2 cells, whereas PABPC3 knockdown had an opposite effect (Fig. 3c and Supplementary Fig. 6c). Given its highest MS/MS count value and significant impact on *MET* downregulation, we selected LRPPRC for further investigation. We further conducted an *MET*-G4-based pull-down assay coupled with western blotting and demonstrated that LRPPRC specifically and preferentially binds to the *MET*-G4 structure, whereas its interaction with the mutated control (*MET*-G4-MUT) is markedly diminished (Fig. 3d).

LRPPRC knockdown in HepG2 cells reduces *MET* mRNA levels to ~30% of control within 24 h, accompanied by a corresponding and significant decrease in MET protein expression (Supplementary Fig. 6d, e). Conversely, LRPPRC overexpression markedly increased both MET mRNA and protein levels (Supplementary Fig. 7a, b). To confirm the generality of this regulatory axis in HCC, we extended our analysis to HCCLM3 cells, a highly metastatic HCC cell line with *MET* amplification and constitutively elevated *MET* expression (Supplementary Fig. 5a)^44^. Strikingly, *MET* knockdown in HCCLM3 cells significantly suppressed proliferation, even under HGF stimulation (Supplementary Fig. 7c–f). Moreover, LRPPRC knockdown in HCCLM3 cells recapitulated the phenotype observed in HepG2 cells, with a marked reduction of both MET mRNA and protein levels (Supplementary Fig. 7g, h). Taken together, LRPPRC knockdown significantly impaired cell proliferation and silenced *MET* expression in two genetically distinct HCC cell lines (Fig. 3e and Supplementary Fig. 7i), establishing LRPPRC as a bona fide upstream regulator of *MET* expression and a functional essential driver of HCC growth.

### The 1025-1394 Domain of LRPPRC is Responsible for Strong *MET*-G4-binding

LRPPRC, a well-known RNA-binding protein present in both the nucleus and mitochondria, plays critical roles in regulating gene transcription, post-transcription, and translation^45^. Importantly, LRPPRC is overexpressed in several cancer types, including HCC^46^, breast cancer^47^, and lung adenocarcinoma^48^, and its elevated expression is closely associated with poor patient survival outcomes^49^. Based on our findings, it is of great interest to investigate whether LRPPRC directly interacts with oncogene promoter G4 structures to activate oncogene expression. To this end, we purified three truncated LRPPRC fragments, namely LRPPRC_53-648_, LRPPRC_650-1024_, and LRPPRC_1025-1394_ (Supplementary Fig. 8a), and evaluated their direct interaction with *MET*-G4 using EMSA experiments. These fragments were designed based on the AlphaFold 3.0-predicted apo form structure of LRPPRC (Fig. 3f), which revealed three major domains connected by two flexible loops^50^. The EMSA results demonstrated that neither LRPPRC_53-648_ nor LRPPRC_650-1024_ exhibited detectable binding activity to the long-flanking *MET*-G4 (Pu36 DNA, Supplementary Fig. 8b). In contrast, LRPPRC_1025-1394_ showed significant binding activity toward the long-flanking *MET*-G4 DNA (Supplementary Fig. 8c, left). At the same time, weaker interaction was observed between LRPPRC_1025-1394_ and the mutated long-flanking *MET*-G4-MUT sequence (Pu36-MUT, Supplementary Fig. 8c, right). Moreover, LRPPRC_1025-1394_ displayed similar specific binding affinities for two *MET*-G4 variants (Pu25m1T and Pu36 DNAs with different flanking lengths, Fig. 3g and Supplementary Fig. 8c), suggesting that flanking length has minimal impact on the binding affinity of LRPPRC_1025-1394_ to the major *MET*-G4.

To investigate whether LRPPRC binding induces structural changes in *MET*-G4, we conducted ECD experiments. The ECD spectra of *MET*-G4 in the presence and absence of the LRPPRC_1025-1394_ fragment were nearly identical (Supplementary Fig. 8d), indicating that LRPPRC binding does not significantly alter the conformation of *MET*-G4. Additionally, Microscale Thermophoresis (MST) experiments were performed to quantify the binding affinity of LRPPRC_1025-1394_ to *MET*-G4 using a FAM-labeled Pu25m1T DNA (Fig. 3h and Supplementary Table 1). The determined *K*_d_ value of the LRPPRC_1025-1394_ fragment to *MET*-G4 was 69.6 nM. In contrast, the binding affinity to the *MET*-G4-MUT sequence was markedly reduced, with a *K*_d_ value of 342.8 nM. Overall, these results demonstrated that the 1025-1394 domain of LRPPRC is responsible for the specific recognition of the major *MET*-G4 structure.

To characterize the binding selectivity of the LRPPRC_1025-1394_ for different types of G4 structures, a competitive EMSA experiment was conducted (Supplementary Fig. 8e). Four representative G4 topologies, parallel (*MYC*-G4)^51^, antiparallel (Bom17-G4)^52^, and two hybrids (*Tel-hybrid1*-G4 and *Tel-hybrid2*-G4)^53,54^, were used, and their folding topologies were validated by ECD spectroscopy (Supplementary Fig. 9). As shown in Supplementary Fig. 8e, incubation of FAM-labeled *MET*-G4 with LRPPRC_1025-1394_ produced a clear upward band shift (lane 2) relative to the probe-free control (lane 1), confirming complex formation. Upon addition of excess unlabeled competitor DNA, the intensity of the shifted band progressively decreased, indicating that the labeled probe was being competitively displaced from its binding site. Lanes 3, 4, 6, and 7, which contained unlabeled *MET*-G4, *MYC*-G4, *Tel-hybrid1*-G4, and *Tel-hybrid2*-G4, respectively, showed strong competition with FAM-labeled *MET*-G4, revealing that LRPPRC_1025-1394_ has high binding activities for these G4s. In contrast, Bom17-G4 (lane 5) showed minimal competition, indicating significantly weaker binding of LRPPRC_1025-1394_ to the antiparallel G4 topology. Taken together, these data reveal that LRPPRC_1025-1394_ exhibits selective binding affinity for parallel and hybrid G4 structures, with significantly weaker interaction toward antiparallel G4s.

### Visualization of the binding model between the LRPPRC_1025-1394_ domain and the *MET*-G4

**Fig. 4:**
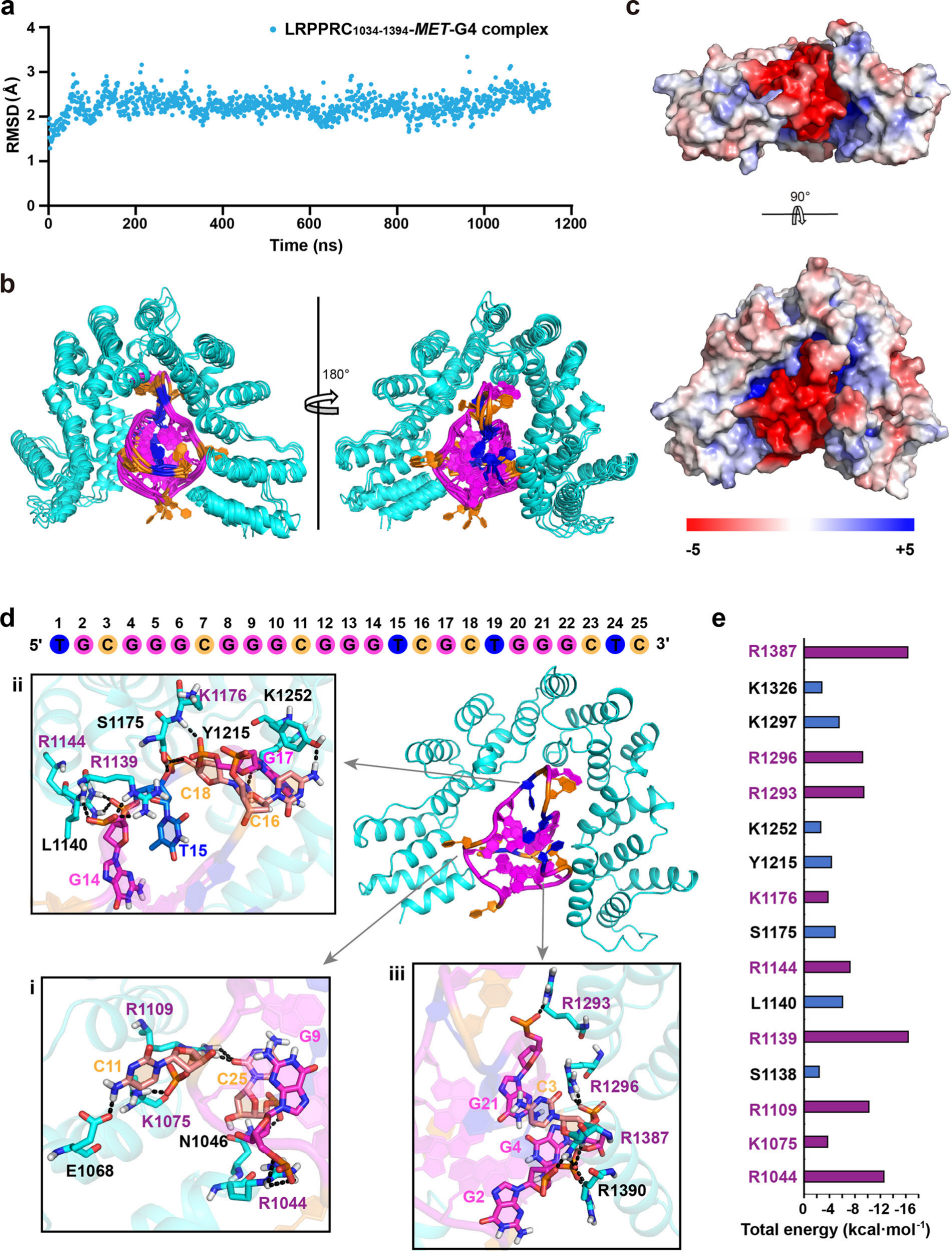
Visualization of the Binding Model of LRPPRC’s 1034-1394 Domain to *MET*-G4. **a** Tracing of RMSD values of the last 1150 ns MD run for all atoms from unrestrained 1250 ns MD simulation of the LRPPRC_1034-1394_−*MET*-G4 complex. **b** Superposition of four representative clustering structures derived from the MD clustering analysis. **c** Electrostatic potential surface views of LRPPRC_1034-1394_−*MET*-G4 complex. **d** Details of the specific contacts between LRPPRC_1034-1394_ and *MET*-G4. Thymine (T, blue), guanine (G, magenta), cytosine (C, orange). Contact residues are listed. **e** Top 16 amino acid residue energy contributions calculated by MMPBSA. Single-letter abbreviations for the amino acid residues referenced throughout the figures **d**, **e** are as follows: R, Arg; K, Lys; Y, Tyr; S, Ser; L, Leu; E, Glu; N, Asn. Source data are provided as a Source Data file.

Since the structural information of the LRPPRC_1025-1394_ domain remains unknown, we utilized AlphaFold 3.0 to predict its structure^50^. The predicted structure revealed that the residues 1025-1033 form a flexible linker region, which was excluded from further analysis (Fig. 3f and Supplementary Fig. 10). Subsequently, molecular docking between the LRPPRC_1034-1394_ domain and the determined *MET*-G4 structure was carried out using HADDOCK 2.4^55^. From the docking results, the complex structure with the lowest binding energy was selected and subjected to unrestrained MD simulations using the Amber package. Over a total of 1250 ns simulations, the RMSD values for all heavy atoms were 2.47 ± 0.42 Å, revealing that the overall LRPPRC_1034-1394_−*MET*-G4 complex structure is stable (Fig. 4a). Cluster analysis of MD trajectories using a hierarchical agglomerative (bottom-up) algorithm yielded four predominant conformational classes (Fig. 4b–d and Supplementary Fig. 11). Notably, the overall structures of the LRPPRC_1034-1394_−*MET*-G4 complexes remained largely similar across all four classes, with the only primary differences observed in the loop conformations of *MET*-G4 (Fig. 4b and Supplementary Fig. 11). The complex structure analysis showed that *MET*-G4 is positioned within the groove of the LRPPRC_1034-1394_ domain, where it forms extensive interactions with LRPPRC, stabilized by both polar and electrostatic contacts (Fig. 4c and Supplementary Fig. 11). Through MM/PBSA calculations, we analyzed the binding energy contributions of individual amino acids and nucleotides, and identified key residues involved in the binding interactions between LRPPRC_1034-1394_ domain and *MET*-G4 (Fig.4e). Specifically, R1044 and K1075 form salt bridges with the phosphate backbone at G9 and C11 of *MET*-G4, while E1068 and R1109 establish hydrogen bonds with bases of G11 and C25 (Fig. 4d, ⅰ). The five-nucleotide loop of *MET*-G4 is located centrally within the groove of LRPPRC_1034-1394_, where it engages in multiple polar interactions with residues R1139, R1144, K1176, and Y1215 in LRPPRC (Fig. 4d, ⅱ). Moreover, extensive polar interactions are observed between R1293, R1296, and R1387 in LRPPRC and G21, G4, and C3 in *MET*-G4, respectively (Fig. 4d, ⅲ). Based on these findings, we performed mutation analysis on the LRPPRC_1034-1394_ fragment. Three mutation variants, mutation-1 (R1044A, K1075A, R1109A), mutation-2 (R1139A, R1144A, K1176A) and mutation-3 (R1293A, R1296A, R1387A), were generated according to MM/PBSA calculation results combined with complex structural analysis (Supplementary Fig. 12a). Subsequently, EMSA gel experiments showed that the binding affinity of mutants 1, 2, and 3 to *MET*-G4 was significantly reduced, with mutant 1 exhibiting slightly stronger binding activity than mutants 2 and 3 (Supplementary Fig. 12b–e). Importantly, none of the three mutation variants were able to form stable complexes with *MET*-G4, indicating that the mutated amino acids are crucial for the specific recognition of *MET*-G4. Taken together, these data suggested that the formation of the LRPPRC_1034-1394_−*MET*-G4 interaction complex is governed by both conformation-based recognition and electrostatic interactions, thereby uncovering a previously unknown mechanism of G4-protein interaction.

### Screening of *MET*-G4-binding Ligands from an In-house Natural Product Library

**Fig. 5:**
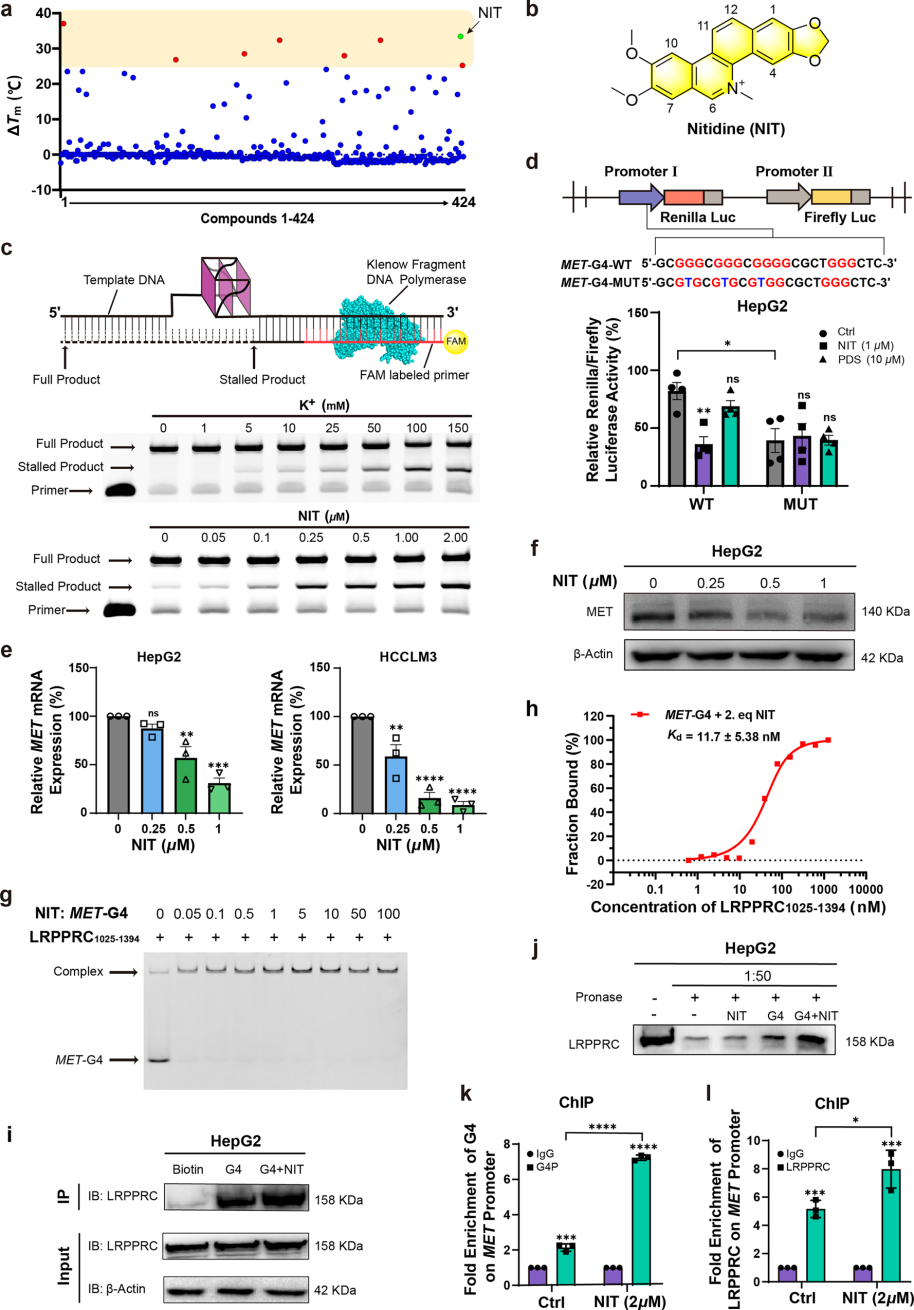
Nitidine effectively suppresses *MET* expression by promoting the formation of a stable LRPPRC-NIT-*MET*-G4 ternary complex. **a** Thermal stabilization values (Δ*T*_m_) of *MET*-G4 induced by various natural small molecules as determined by a FRET-melting assay. Compounds selected for further investigation are highlighted within a yellow box, with nitidine indicated by a green circle. Conditions: 0.2 μM labeled DNA, 10 μM compound, pH 7.0, 10 mM K^+^. **b** Chemical structure of nitidine with numbering. **c** Schematic illustration of a DNA polymerase stop assay: A FAM-labeled primer hybridizes to a DNA template containing the wild-type human *MET*-G4 forming sequence, followed by extension using Klenow Fragment (exo-) DNA polymerase. The resulting products, comprising both stalled and full-length products, are labeled. The experiment was repeated three times independently with similar results; a representative image is shown. **d** Schematic diagram illustrating the construction of the luciferase reporter plasmids containing the *MET* promoter. The core G4-forming sequences and the corresponding mutant sequences were listed (top). Relative luciferase activities of wild-type (wt) and mutant (mut) *MET* promoters treated with NIT or PDS in HepG2 cells were shown (bottom). The data represent mean ± S.E.M. of results from four independent experiments. *p = 0.015, **p = 0.0033, ns: not significant, two-tailed *t*-test. **e** RT-qPCR analysis of *MET* mRNA levels in HepG2 and HCCLM3 cells treated with nitidine for 48 h. The data represent mean ± S.E.M. of results from three independent experiments. ns: not significant, **p = 0.005, ***p = 0.0002 (in HepG2); **p = 0.008, ****p < 0.0001 (in HCCLM3). Statistical significances were analyzed using one-way ANOVA. **f** Representative western blotting analysis of MET protein expression in HepG2 cells treated with nitidine for 48 h is shown. Three independent experiments were performed with similar results. **g** EMSA gel data showing the formation of LRPPRC_1025-1394_-NIT-*MET*-G4 ternary complex. Condition: 1 μM LRPPRC_1025-1394_, 0.1 μM FAM-labeled DNA, pH 8.0, 100 mM K^+^. The experiment was repeated three times independently with similar results; a representative image is shown. **h** The fraction bound activity is plotted against the concentration of LRPPRC_1025-1394_ protein in the presence of nitidine. Experiments were run in triplicate. Data are presented as mean ± SD. For some points, the error bars are shorter than the height of the symbol and are not shown. The calculated *K*_d_ value is shown. **i** Representative pull-down combined with western blotting analysis of the interaction between LRPPRC and *MET*-G4 in HepG2 cells treated with or without nitidine is shown. Three independent experiments were performed with similar results. **j** Drug Affinity Responsive Target Stability (DARTS) analysis of nitidine, *MET*-G4, and nitidine-*MET*-G4 complex binding to LRPPRC. Interactions were assessed by monitoring LRPPRC stability through western blotting detection. The experiment was repeated three times independently with similar results; a representative image is shown. **k** and **l** ChIP assays were used to evaluate the enrichment of LRPPRC protein and G4P in the *MET* promoter with and without 2 μM nitidine treatment in HepG2 cells, respectively. The data represent mean ± S.E.M. of results from three independent experiments. ***p = 0.0007 (**k**), ****p = 0.0000002 (**k**), ****p = 0.000005 (**k**), ***p = 0.0003 (**l**), ***p = 0.0008 (**l**), *p = 0.0298 (**l**). Statistical significances were analyzed using a two-tailed *t*-test. Source data are provided as a Source Data file.

With the determined LRPPRC−*MET*-G4 interaction complex system, our next objective is to identify effective ligands that could interfere with the LRPPRC−*MET*-G4 interaction complex, thereby downregulating *MET*. Subsequently, we conducted a FRET-melting assay using dual-labeled *MET*-G4 DNA with 5′-TAMRA and 3′-FAM to screen potential *MET*-G4 stabilizers (Supplementary Fig. 13a). At room temperature, the folded form of *MET*-G4 was predominant, resulting in quenched FAM fluorescence. However, as the temperature increased, the *MET*-G4 unfolded into an extended single-strand form, exhibiting high FAM fluorescence. Compounds that stabilize the *MET*-G4 can increase the *T*_m_ value of the *MET*-G4, making the assay suitable for ligand screening. Using the FRET-melting assay, we screened potential *MET-*G4 stabilizers from our in-house natural product library, consisting of 424 compounds (Fig. 5a). As a result, 8 compounds increased the *T*_m_ values of *MET*-G4 by more than 25 °C, which were defined as hit compounds for further analysis.

To determine the impact of 8 compounds on the *MET* oncogene transcription levels, we performed an RT-qPCR analysis in HepG2 cancer cells. The results showed that nitidine (NIT) exhibited a significant reduction of approximately 60% in *MET* mRNA levels at a concentration of 1 μM, whereas the other seven compounds did not show strong effects under the same conditions (Supplementary Fig. 13b). Therefore, nitidine was selected as the lead compound for further investigation (Fig. 5b).

### Nitidine strongly binds and stabilizes *MET*-G4 within an extended DNA context

To gain further insight into the binding activity between nitidine and *MET*-G4, we conducted a series of biophysical experiments. The ^1^H NMR titration experiments revealed that upon the addition of nitidine, the 12 imino protons characteristic of *MET*-G4 experienced upfield shifts and broadening even at high ligand concentrations (Supplementary Fig. 13c and Supplementary Fig. 14). This demonstrated that the binding of nitidine to *MET*-G4 occurred in a medium-to-fast exchange rate on the NMR time scale and involved several coexisting binding models. Furthermore, the presence of a small proportion of DMSO in the samples was proved to have no effect on NMR spectra quality (Supplementary Fig. 15).

Subsequently, ECD spectra analysis showed that the parallel topology of *MET*-G4 was maintained when it forms a complex with nitidine. An increase in the *T*_m_ value by 26 °C for *MET*-G4 was observed in the presence of 2 equivalents of nitidine, comparable with the FRET-melting result (Supplementary Fig. 16a and Fig. 5a). The dissociation constant (*K*_d_) between nitidine and *MET*-G4 was determined through a fluorescence-based experiment (Supplementary Fig. 16b). Additionally, EMSA gel data revealed that both free *MET*-G4 and its complex with nitidine existed in monomeric G4 states (Supplementary Fig. 16c). Overall, these findings demonstrated that nitidine is a potent *MET*-G4 binder. Moreover, nitidine exhibits strong binding affinity for diverse G4 structures, as evidenced by substantial thermal stabilization (Δ*T*_m_ up to 26 °C) and tight binding (K_d_ values in the submicromolar to micromolar range) across parallel, antiparallel, and hybrid G4 topologies (Supplementary Fig. 17, 18).

To assess the potential of nitidine in stabilizing *MET*-G4 within an extended DNA context that mimics genomic DNA, we performed a DNA polymerase stop assay using a DNA template containing the wild-type *MET*-G4 forming motif (Fig. 5c). The presence of a G4 secondary structure on the template strand impedes the efficiency of the Klenow fragment (exo-) DNA polymerase in synthesizing the complementary strand DNA, which is sensitive to K^+^ and G4-ligand concentrations^56^. As shown in Fig. 5c, increasing the K^+^ concentration in the buffer resulted in noticeable stalled products, indicating the formation of *MET*-G4 in the extended DNA context. Moreover, the addition of nitidine significantly increased the amount of stalled products in a dose-dependent manner in 5 mM K^+^-containing solutions, demonstrating its ability to stabilize *MET*-G4 structure in long DNA contexts and thereby inhibit DNA polymerase. Collectively, these findings provide evidence for the potential formation of *MET*-G4 within genomic DNA and suggest that small molecules can target *MET*-G4 to modulate gene expression, including replication and transcriptional regulation.

### Nitidine suppresses *MET* expression via an *MET*-G4-dependent mechanism

To ascertain the potential role of *MET*-G4 in transcriptional regulation, we performed a dual-luciferase reporter assay using HepG2 cells (Fig. 5d). The *MET* proximal promoter region spanning from -265 to 40 bp was cloned into the pSiCHECK-2 vector upstream of the Renilla luciferase reporter gene as the *MET*-G4-WT construct. Additionally, a control construct, *MET*-G4-MUT, was generated by introducing G-to-T mutations into the *MET*-G4-forming region, which abolished its ability to form *MET*-G4. Both constructs were transfected into HepG2 cells, and firefly luciferase signals served as an internal control. The results showed that the luciferase activity of the *MET-*G4-WT construct was significantly higher than that of the *MET-*G4-MUT construct (Fig. 5d), suggesting that *MET-*G4 functions as a transcriptional enhancer. Furthermore, treatment with nitidine significantly suppressed the luciferase activity of the *MET-G4*-WT construct but did not affect the *MET-*G4-MUT construct, suggesting that nitidine stabilizes the *MET*-G4 for gene expression inhibition. Additionally, both constructs showed unchanged luciferase activity upon exposure to PDS, a well-known G4 stabilizer, implying that PDS does not target *MET*-G4.

To further assess the impact of two compounds on *MET* oncogene expression, we quantified MET mRNA and protein levels in HepG2 and HCCLM3 hepatocellular carcinoma cells after compound treatment. The treatment concentrations were determined based on their IC_50_ values (Supplementary Fig. 19a, b). The results showed that nitidine dose-dependently downregulated MET mRNA and protein levels in both cancer cells, while PDS treatment did not affect MET expression (Fig. 5e, f, Supplementary Fig. 19c–e). To investigate MET’s functional role in nitidine’s anticancer activity, we employed HGF-stimulated HepG2 cells, a model with an active *MET* signaling pathway^57^. In this system, nitidine suppressed cell viability more potently upon HGF stimulation than under non-stimulated conditions (Supplementary Fig. 20a). Both MET knockdown and nitidine treatment alone significantly suppressed cell proliferation; critically, their combination yielded a synergistic antiproliferative effect (Supplementary Fig. 20b). This synergistic effect was consistently observed in HCCLM3 cells (Supplementary Fig. 20b), indicating that nitidine’s antiproliferative activity stems not only from *MET* signaling inhibition but also from complementary, *MET*-independent pathways, aligning with its established polypharmacological profile^58^. Moreover, LRPPRC knockdown, whether applied alone or in combination with nitidine, produced comparable inhibitory effects on proliferation and MET protein inhibition in both HGF-stimulated HepG2 and HCCLM3 cells (Supplementary Fig. 20c, d). Taken together, these data support an *MET*-G4-dependent mechanism underlying nitidine-induced *MET* downregulation. Importantly, nitidine exhibited reduced cytotoxicity in normal human hepatocytes (THLE-2 and WRL68 cells), with IC₅₀ values significantly higher than those in cancer cells (Supplementary Fig. 20e). Furthermore, nitidine treatment induced significantly apoptosis and G2/M phase arrest in HepG2 and HCCLM3 cells (Supplementary Figs. 21–23), a characteristic phenomenon commonly observed in G4-targeted small molecules^56^.

We next investigated nitidine’s anticancer activity in two *MET*-dependent lung cancer cell lines, EBC-1 and H1993, both harboring genomic *MET* amplification and exhibiting consequent MET protein overexpression (Supplementary Fig. 24a)^44^. As a result, nitidine significantly suppressed both MET mRNA and protein levels in a concentration-dependent manner (Supplementary Fig. 24b–d), whereas capmatinib, a clinically approved MET tyrosine kinase inhibitor^44^, failed to reduce *MET* expression levels consistently across doses or cell lines (Supplementary Fig. 24b–d). This contrast aligns with their distinct mechanisms: capmatinib selectively inhibits MET phosphorylation and downstream signaling without affecting total MET abundance, while nitidine mainly suppresses *MET* transcription, thereby reducing overall MET expression. Moreover, nitidine exhibited reduced anticancer potency in MET-low expression H460 cells (Supplementary Fig. 24e). Taken together, these findings demonstrated nitidine downregulates *MET* expression likely through a *MET*-G4-mediated mechanism in *MET*-dependent cancer cell lines.

### Nitidine functions as a molecular glue to promote the formation of a stable LRPPRC−NIT−*MET*-G4 ternary complex

We have demonstrated that nitidine downregulates *MET* oncogene expression via an *MET*-G4-mediated mechanism, with LRPPRC being a major *MET*-G4-binding protein. To ascertain whether nitidine interferes with the interaction between LRPPRC and *MET*-G4, we conducted a series of biophysical experiments. Firstly, EMSA gel analysis revealed that nitidine significantly increased the amount of LRPPRC−*MET*-G4 interaction complexes (Fig. 5g), indicating the formation of stable LRPPRC−NIT−*MET*-G4 ternary complexes. Subsequently, a six-fold decrease in the *K*_d_ value between LRPPRC and *MET*-G4 was observed upon nitidine addition (Figs. 3h and Fig. 5h), suggesting that nitidine stabilized the LRPPRC−*MET*-G4 interaction complex. Moreover, the *MET*-G4-based pull-down and western blotting analyses showed that nitidine enhanced the enrichment of the LRPPRC−*MET*-G4 interaction complex (Fig. 5i), providing further evidence for the formation of LRPPRC−NIT−*MET*-G4 ternary complexes. Furthermore, the drug affinity responsive target stability (DARTS) assays showed that the NIT−*MET*-G4 complex significantly stabilizes endogenous LRPPRC protein, whereas individual nitidine or *MET*-G4 exhibited only weak effects (Fig. 5j). Additionally, the bio-layer interferometry (BLI) assays demonstrated that nitidine has only negligible binding activity to the LRPPRC_1034-1394_ fragment (Supplementary Fig. 25a). Taken together, these findings demonstrated that nitidine acts as a molecular glue to promote the formation of a stable LRPPRC−NIT−*MET*-G4 ternary complex.

### Nitidine stabilized the LRPPRC−*MET*-G4 interaction complex in cancer cells

Based on the above findings, we hypothesized that nitidine binds to *MET*-G4, thereby facilitating the recruitment of LRPPRC and promoting the formation of a stable LRPPRC−NIT−*MET*-G4 ternary complex. This interaction may alter the structure and function of LRPPRC, ultimately leading to *MET* downregulation. To test this hypothesis, we performed ChIP experiments using either a G4- or LRPPRC-specific peptide/antibody^35,37^.

We utilized G4-specific peptide G4P for G4-ChIP experiments and confirmed its binding activity to *MET*-G4 in the presence of nitidine (Supplementary Fig. 25b–e)^37^. Meanwhile, nitidine treatment significantly reduced *MET* mRNA levels at 6 h post-treatment, whereas LRPPRC protein levels remained unchanged (Supplementary Fig. 25f, g). Subsequently, G4-ChIP-qPCR analysis showed significant enrichment of the *MET*-G4-associated promoter sequence, thereby confirming the presence of *MET*-G4 within the *MET* promoter region in human cancer cells. Notably, nitidine treatment resulted in a four-fold increase in this enrichment, providing evidence of its role in stabilizing *MET*-G4 within cancer cells (Fig. 5k). LRPPRC-ChIP-qPCR data revealed that LRPPRC was enriched in the same region where the *MET*-G4 forms, suggesting an interaction between LRPPRC and *MET*-G4 in cancer cells (Fig. 5l). Remarkably, nitidine treatment significantly enhanced LRPPRC occupancy on the *MET* promoter, supporting the formation of the LRPPRC−NIT−*MET*-G4 ternary complex in cancer cells (Fig. 5l). Moreover, we performed an immunofluorescence (IF) assay using a Cy5-labeled wild-type *MET*-G4-forming probe (G4-WT) and a mutated one (G4-MUT), a *MET*-G4-deficent control sequence, to visualize *MET*-G4 formation in cells and assess its interaction with nitidine and the LRPPRC protein (Supplementary Fig. 26a, b). These probes were transfected into HEK293T cells, either unmodified or with LRPPRC overexpression. The successful overexpression of LRPPRC in HEK293T cells was confirmed by RT-qPCR and western blotting (Supplementary Fig. 26c, d). To specifically detect intracellular *MET*-G4 formation, cells were co-stained with a FAM-conjugated G4-specific antibody (FAM-G4P). As a result, we observed robust colocalization between the Cy5-labeled G4-WT probe and FAM-G4P, which was increased significantly upon nitidine treatment. In contrast, the G4-MUT probe showed negligible colocalization with FAM-G4P, both with or without nitidine treatment, confirming that an intact G4 structure is essential for recognition (Supplementary Fig. 26a, b). Notably, LRPPRC overexpression further enhanced G4-WT/FAM-G4P colocalization signals, especially with nitidine treatment, demonstrating that LRPPRC engages functional *MET*-G4 structures and that nitidine potentiates this interaction (Supplementary Fig. 26a, b). Overall, these findings demonstrated that nitidine stabilized the LRPPRC−*MET*-G4 interaction complex within human cancer cells, suggesting a biomolecular target for cancer therapy.

### Nitidine inhibits tumor progression in vivo by downregulating *MET* expression

**Fig. 6:**
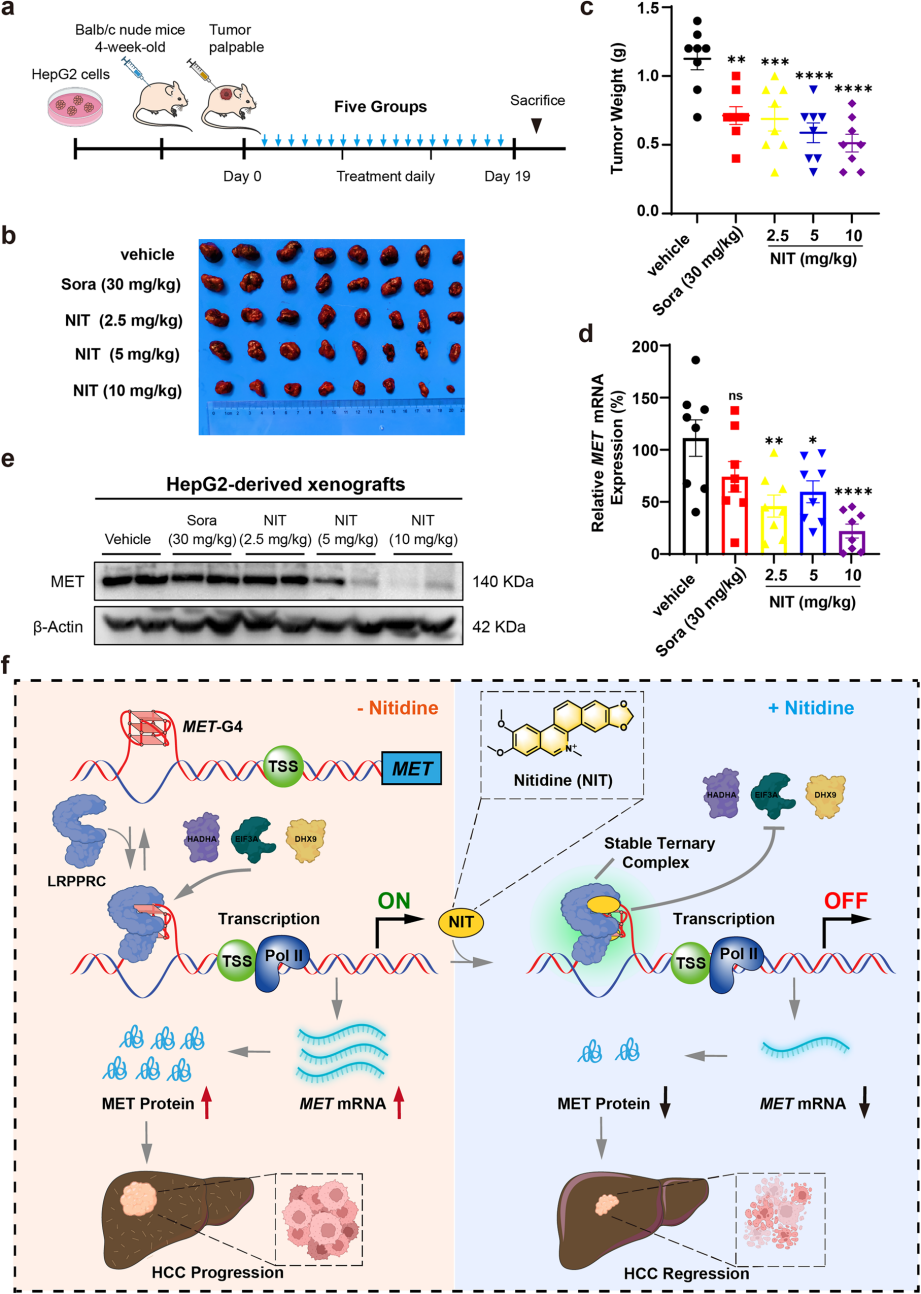
Nitidine inhibits HCC progression in vivo by downregulating *MET* expression. **a** Schematic diagram of HepG2-derived xenograft mouse model. Created in BioRender. Li, Y. (2026) https://BioRender.com/8q4p3q6. **b** and **c** Nude mice bearing HepG2-derived xenografts were orally administered sorafenib (30 mg/kg/day) or intraperitoneally administered nitidine (2.5, 5, or 10 mg/kg/day), *n* = 8 biologically independent mice. **b** Representative images of excised tumors and **c** corresponding tumor weights were shown. Data are presented as mean ± SEM. **p = 0.0014, ***p = 0.0007, ****p < 0.0001, ns: not significant, one-way ANOVA. **d** Relative mRNA expression levels of *MET* in HepG2-derived xenografts following sorafenib or nitidine treatment. *n* = 8 biologically independent samples. Data are presented as mean ± SEM. *p = 0.0217, **p = 0.0028, ****p < 0.0001, ns: not significant, one-way ANOVA. **e** Protein expression levels of MET in HepG2-derived xenografts following sorafenib or nitidine treatment. **f** The proposed epigenetic regulatory mechanism of the LRPPRC−*MET*-G4 interaction complex: Nitidine stabilizes the LRPPRC−*MET*-G4 interaction complex and facilitates the formation of a stable LRPPRC−NIT−*MET*-G4 ternary complex. This ternary complex alters the structure and function of LRPPRC, thereby reducing the transcription of the *MET* oncogene and ultimately suppressing HCC progression in mice. Created in BioRender. Li, Y. (2026) https://BioRender.com/5fl1po3. Source data are provided as a Source Data file.

To further investigate the therapeutic potential of nitidine in targeting the LRPPRC−*MET*-G4 interaction complex for cancer therapy, we conducted in vivo experiments using HepG2-derived xenograft models (Fig. 6a). Sorafenib, an FDA-approved first-line drug for advanced liver cancer treatment, was used as a positive control^59^. Compared to both vehicle and sorafenib-treated groups, nitidine treatment significantly inhibited tumor growth in a dose-dependent manner (Fig. 6b), as evidenced by reduced tumor volume and weight (Fig. 6c and Supplementary Fig. 27a). Moreover, nitidine treatment significantly decreased both MET mRNA and protein levels in HepG2-derived xenografts (Fig. 6d, e). During the treatment period, no significant changes in body weight were observed, suggesting no major systemic toxicity (Supplementary Fig. 27b). Additionally, serum ALT and AST levels remained largely unchanged at the end of the treatment (Supplementary Fig. 27c, d), supporting the safety profile of nitidine. Hematoxylin and eosin (H&E) staining and immunohistochemical (IHC) analysis further validated the tumor inhibition effect of nitidine by downregulating MET expression (Supplementary Fig. 27e).

To assess nitidine’s efficacy in *MET*-amplified lung adenocarcinoma, we extended our evaluation to H1993-derived xenografts (Supplementary Fig. 28a). Herein, nitidine again significantly inhibited tumor growth and suppressed MET expression level (Supplementary Fig. 28b–f), achieving comparable antitumor activity compared to the clinically approved MET inhibitor of capmatinib. H&E staining and IHC analysis further validated the tumor inhibition effect of nitidine by downregulating MET expression (Supplementary Fig. 28g). Notably, while all treatments were well tolerated during the initial 13 days, progressive body weight loss emerged after day 13 in the nitidine group, suggesting a narrow therapeutic window that warrants dose optimization or intermittent dosing strategies in future studies (Supplementary Fig. 28h). Nevertheless, H&E analysis of major organs (heart, liver, spleen, lung, and kidney) revealed no significant pathological alterations, indicating the absence of overt organ toxicity at the histological level (Supplementary Fig. 28i). Collectively, these in vivo and in vitro data demonstrate that nitidine exerts robust, G4-based antitumor effects across distinct types of cancer models by disrupting the LRPPRC–*MET*-G4 regulatory axis, offering a therapeutic strategy for MET-driven tumors.

## Discussion

The current study has uncovered several findings. We elucidated the high-resolution NMR solution structure of the human *MET* oncogene proximal promoter G4 (*MET*-G4), which provides a foundation for designing *MET*-G4-targeted ligands and other technological applications. The architecture of loop and flanking residues plays a pivotal role in defining the specific *MET*-G4 conformations, expanding the pool of known G4 structures. Notably, we identified LRPPRC as the key *MET-*G4-binding protein, and this interaction plays a crucial role in the epigenetic upregulation of the *MET* oncogene. Moreover, we identified the 1034-1394 domain of LRPPRC as the critical region mediating high-affinity binding to *MET*-G4 and integrated AlphaFold 3.0, molecular docking, and Amber MD simulations to build a structurally refined LRPPRC−*MET*-G4 complex model. Furthermore, we discovered the natural alkaloid nitidine as a potent molecular glue that effectively stabilizes the LRPPRC−*MET*-G4 interaction complex, thereby silencing the *MET* signaling pathway. Unlike conventional molecular glues, which typically stabilize protein-protein interactions^60,61^, nitidine represents an example of a small molecule that stabilizes a G4–protein interface. This G4–protein stabilization mechanism opens a promising avenues for targeted anticancer therapeutics.

Beyond these findings, this study has several limitations that warrant careful consideration. Our work primarily focuses on the LRPPRC−*MET*-G4 interaction, whereas G4-based pull-down and siRNA-mediated knockdown experiments identified additional *MET*-G4−binding proteins, including EIF3A, DHX9, and HADHA, all implicated in *MET* signaling regulation (Fig. 6f). The functional interplay among these proteins (e.g., cooperative or competitive binding, hierarchical assembly, or allosteric crosstalk), their collective influence on *MET* transcription, and how nitidine modulates this regulatory network remain unclear. Addressing these questions is essential to map the broader landscape of *MET*-G4−mediated gene control. Second, although nitidine robustly stabilizes the LRPPRC−*MET*-G4 complex across multiple *MET*-dependent cancer cell lines, resulting in coordinated downregulation of both MET mRNA and protein, it lacks sufficient selectivity to function as a “pure” molecular glue specific to this interface. Its polypharmacological profile complicates mechanistic interpretation^62^, as off-target contributions cannot be excluded. Nevertheless, nitidine’s preferential cytotoxicity in *MET*-addicted cancer cells strongly supports the biological relevance and therapeutic potential of pharmacologically targeting the LRPPRC−*MET*-G4 interface. Importantly, nitidine is not intended as a clinical candidate; rather, it serves as a chemical probe to establish this previously unexplored, targetable node. Third, high-resolution structures of the ternary LRPPRC–NIT–*MET*-G4 and binary *MET*-G4–NIT complexes remain undetermined. NMR titration of nitidine into *MET*-G4 induced pronounced broadening and substantial chemical shift perturbations in the imino proton signals (Supplementary Fig. 13c), indicating dynamic, multi-modal binding with rapid-to-medium exchange among several distinct conformations, rendering the high-resolution structural determination of the complexes currently infeasible. Notably, structurally related natural alkaloids, berberine and coptisine, have shown specific binding to parallel G4s, including *KRAS*-G4, *MYC*-G4, and *PDGFR-β*-G4^63–65^. Given their shared isoquinoline scaffold and cationic planar core, nitidine is predicted to bind *MET*-G4 primarily via π–π stacking and electrostatic interactions at the 5′- and 3′-terminal G-tetrads. However, its unique ring-fused pattern and extended aromatic conjugation system enhance overall binding affinity, yet at the cost of reduced specificity for parallel G4s, including *MET*-G4, compared to berberine and coptisine (Supplementary Fig. 29). To advance therapeutic targeting of LRPPRC−*MET*-G4 interaction, future efforts should prioritize two key directions: (i) high-resolution structural characterization of the Ligand−*MET*-G4 and LRPPRC−Ligand−*MET*-G4 complexes using integrated NMR, X-ray, and cryo-EM approaches; and (ii) rational design of next-generation molecular glues targeting the LRPPRC−*MET*-G4 interaction complex, achieved either through scaffold hopping from the isoquinoline core or via strategic side-chain derivatization, to simultaneously improve potency, selectivity, and drug-like properties.

G4s have long been considered effective anticancer targets, particularly as alternative strategies for addressing undruggable and resistance-associated protein targets^61,66^. However, the development of effective G4-targeting drugs faces significant challenges, mainly due to the issues of selectivity. G4s share a similar G-tetrad core, a defining characteristic of this class of nucleic acid structures. However, this structural similarity complicates the design of drugs that selectively target individual G4s^33^. Efforts have been made to develop inhibitors that interfere with G4-protein interactions, whereas the intrinsic targets remain individual G4s^67^. Our current studies have identified a rare G4-protein stabilizer that promotes the formation of a unique LRPPRC−NIT−*MET*-G4 ternary complex. This finding provides potential avenues for G4-targeting drug development, possibly addressing the selectivity problem by shifting the focus to G4-protein complexes rather than G4 structures alone. We believe that targeting the LRPPRC−*MET*-G4 interaction complex with molecular glues holds promise for developing effective anticancer drugs, potentially accelerating the market entry of the G4-targeted drugs.

The characteristic function of LRPPRC in mitochondrial biology is well- documented^68^. However, its role in the nucleus remains poorly understood, despite its detectable presence in this compartment alongside predominant mitochondrial localization^69^. LRPPRC has been shown to form a complex with SLIRP, functioning as a universal RNA chaperone that binds to mitochondrial mRNAs^70^. Notably, SLIRP has been shown to bind to DNA G4s derived from human telomeres and oncogene promoters^31^. In this study, we identified a nuclear function of LRPPRC. Specifically, the LRPPRC protein functions as an epigenetic regulator of the *MET* signaling pathway. By directly binding to the *MET*-G4, LRPPRC may facilitate the recruitment of essential transcription factors or chromatin proteins, thereby contributing to the epigenetic upregulation of the *MET* oncogene (Fig. 6f)^25,71^.

In conclusion, our findings demonstrated that the formation of the LRPPRC−*MET*-G4 interaction complex is critical for *MET* oncogene upregulation. Moreover, small molecules like nitidine can induce the formation of stable LRPPRC−NIT−*MET*-G4 ternary complexes. This interaction may help to alter the structure and function of LRPPRC, ultimately leading to *MET* downregulation and the death of cancer cells. Collectively, this study establishes the LRPPRC−*MET*-G4 interaction complex as a promising biomolecular target for future anticancer drug development.

## Methods

### Ethics statement

Our study complied with all relevant ethical regulations. Research involving animals was approved by the University Committee on Use and Care of Animals of the China Pharmaceutical University (CPU, approval No.YSL-202512002).

### Sample preparation and cell culture

All DNA oligonucleotides (both labeled and unlabeled) were purchased from Sangon Biotech (Shanghai) Co., Ltd with PAGE or HPLC purification processes. The DNA was dissolved in a buffer containing 37.5 mM KCl, 12.5 mM K_2_HPO_4_/KH_2_PO_4_ (pH 7.0), and 10% D_2_O/90% H_2_O. The DNA concentrations were measured at 260 nm using a UV spectrometer. The plasmids were constructed by Saisuofei (Wuxi) Co., Ltd. All plasmid constructs were verified by Sanger sequencing. Plasmids are available from the corresponding author upon reasonable request.

Nitidine (CAS NO. 13063-04-2) was purchased from Chengdu PuSi Biotechnology Co., Ltd., and Pyridostatin (CAS NO. 1085412-37-8) was purchased from Selleck (China). Both compounds were stored in DMSO-*d*_*6*_ at a final concentration of 40 mM. Sorafenib and capmatinib were purchased from MedChemExpress (China). All cells obtained from Shanghai Cell Bank of the Chinese Academy of Sciences were cultured in DMEM or 1640 supplemented with 10% fetal bovine serum (FBS) and 1% penicillin-streptomycin solution at 37 °C in a humidified incubator with 5% CO_2_. All cell lines were authenticated by short tandem repeat (STR) profiling and were routinely tested for mycoplasma contamination.

### Cell growth and viability assay

For the analysis of cell growth, HepG2 cells were seeded in 96-well plates at a density of 3000 cells per well and subsequently transfected with either si*LRPPRC* or a scrambled control. Cell growth was monitored at 0-, 24-, 48-, and 72-h post-transfection. For cell viability assays, HepG2 and HCCLM3 cells were seeded at a density of 8000 cells per well in 96-well plates and treated with varying concentrations of NIT or PDS for 48 h. Cell viability was assessed by measuring the absorbance at 450 nm with the use of CCK-8 reagent.

### DMS footprinting

The oligonucleotide was 3′-end-labeled with FAM and purified by HPLC. The methylation was initiated by treatment with 4% (vol/vol) dimethyl sulfate (DMS) for 7 min at room temperature in a total volume of 200 μL, and the reaction was quenched immediately by adding 70 μL of stop buffer (1.3 M NaOAc, 2.7 M *β*-mercaptoethanol, 3 mg/mL sperm DNA) per sample. Following phenol/chloroform extraction and ethanol precipitation, the DNA was resuspended in 100 μL of 10% (vol/vol) piperidine aqueous solution and incubated at 90 °C for 30 min to cleave methylated residues. The samples were then subjected to chloroform extraction and ethanol precipitation. Subsequently, the precipitated DNA was dissolved in 80% (vol/vol) deionized formamide in water, denatured at 95 °C for 5 min, and loaded onto a 20% denaturing polyacrylamide gel in the 1 × TTE running buffer. Reaction products were visualized on a GelDoc-Go Imaging System (Bio-Rad, USA) and digitized using the ImageQuant 5.2 software.

### NMR spectroscopy

All NMR experiments were performed using an unlabeled oligonucleotide sample at natural isotopic abundance (without ¹³C or ¹⁵N enrichment). For the 2D NOESY, COSY, and HSQC experiments, the DNA sample was prepared at a concentration of 1.51 mM in a 25 mM K^+^-containing buffer (pH 7.0), with 90% H₂O/10% D₂O as the solvent. NMR spectra were collected using a Bruker AV-600 spectrometer equipped with a QCI Cryoprobe. The w5 water suppression was applied to all experiments, unless otherwise indicated. NOESY experiments were conducted at 5, 15, 25, and 35 °C, with mixing times of 80, 300, 350, and 400 ms, respectively, and NS = 32 (32 scans per t₁ increment). The data matrix consisted of 4096 complex points in TD (F2) and 480 in TD (F1). The DQF-COSY spectrum was acquired at 25 °C, while ^1^H-^13^C HSQC spectra were obtained at both 25 °C and 35 °C. Chemical shift calibration was indirectly performed for ^13^C relative to DSS and directly for ^1^H based on the water signal relative to DSS. NMR spectral processing and analysis were performed using Topspin 3.5 (Bruker) and Sparky (UCSF) software, respectively.

### Structure calculation

The structures of *MET*-G4 (Pu25m1T DNA) were determined using Xplor-NIH and the Amber 20 package incorporating the NOE distance information^72,73^. NOE-based distance restraints were categorized as strong (2.9 ± 1.1 Å), medium (4.0 ± 1.5 Å), weak (5.5 ± 1.5 Å), and very weak (6.0 ± 1.5 Å), as determined from NOESY spectra acquired with mixing times of 80 and 350 ms. Ambiguous resonances were designated as 5.0 ± 2.0 Å. Exchangeable protons were assigned as medium (4.0 ± 1.2 Å), weak (5.0 ± 1.2 Å), and very weak (6.0 ± 1.2 Å). A total of 48 hydrogen bond restraints were applied for the 12 tetrad guanines. Glycosidic torsion angles were constrained to 170°–310° and 200°–280° for anti-conformations in loop regions and within the G-tetrad, respectively. The Amber OL15 force field was used for DNA. Initially, 100 starting structures of Pu25m1T DNA were generated using Xplor-NIH, followed by simulated annealing using the sander module of the Amber 20 package. Subsequently, the 20 lowest-energy structures underwent molecular dynamics calculations using the pmemd module of the Amber 20 package in the presence of K^+^ cations and TIP3P water. The last 500 ps of the trajectories were averaged and energy-minimized for 500 steps in vacuum after removing water molecules and cations. The final ensemble and deposition comprised the 15 lowest energy structures, analyzed and visualized using VMD and PyMOL software^74,75^.

### Protein-G4 docking with HADDOCK2.4 web server

The structural model of LRPPRC_1034-1394_ was predicted using AlphaFold 3.0, while the solution NMR structure of the *MET*-G4 was determined experimentally in our lab. Protein-G4 docking was performed using the HADDOCK2.4 web server (EASY mode) with default parameters^55^. Residues 1034-1328 of LRPPRC were defined as active residues, and all residues of the *MET*-G4 were treated as active. Docking yielded 12 clusters, and the top-ranked model with the lowest binding-energy structure was selected for subsequent molecular dynamics simulation.

### Unrestrained molecular dynamics (MD) Simulation

The top-ranked LRPPRC_1034-1394_−*MET*-G4 complex structure from HADDOCK2.4 docking was neutralized with K^+^ cations, two of which were explicitly positioned between adjacent G-tetrads. The system was solvated in a 10.0 Å truncated octahedral box of TIP3P water using tleap, and 150 mM K⁺/Cl⁻ ions were added to mimic physiological ionic strength. The Amber OL15 and ff19SB force fields were used for DNA and protein, respectively. The initial equilibration protocol consisted of two energy minimization steps: 500 steps of steepest descent minimization followed by conjugate gradient minimization, both performed while applying positional restraints (force constant = 25 kcal·mol⁻¹·Å⁻²) to the DNA backbone atoms. Subsequently, the system was gradually heated from 100 K to 300 K over 20 ps under NVT conditions. Under constant pressure and temperature control of 300 K, the final MD simulations were run using PMEMD CUDA Amber 20 for 1250 ns^76^. Snapshots were taken every five picoseconds. No restraints were applied during the whole MD simulations. Trajectory analysis (last 1150 ns) employed VMD and PyMOL for structural visualization and RMSD calculation (all-atom RMSD relative to the top model). Binding free energy contributions per residue (amino acid and nucleotide) were calculated using MM/PBSA. Hierarchical agglomerative (bottom-up) clustering identified dominant conformational states, and representative average structures from each major cluster were used for final structural interpretation and presentation.

### Circular dichroism (CD)

CD experiments were performed on a Jasco-1500 spectropolarimeter (Jasco Inc., Japan). DNA samples were prepared at a final concentration of 20 μM in a buffer containing 37.5 mM K_2_HPO_4_/KH_2_PO_4_ and 11.5 mM KCl (pH 7.0). Prior to analysis, the DNA was annealed by heating to 95 °C for 5 min followed by gradual cooling to room temperature. Nitidine was titrated at specified concentrations and incubated with DNA for 3 h at room temperature. CD spectra were collected using a 1 mm path length quartz cuvette at 25 °C. The algorithm of means-movement was adopted for smoothing, and the baseline correction was applied by subtracting the buffer. For CD melting, samples were heated from 25 °C to 95 °C with a heating rate of 2 °C/min, and the CD ellipticity at 264 nm was continuously recorded. The melting temperature (*T*_m_) was then obtained at the intersection between the median of the fitted baselines and the melting curve.

For protein-DNA binding, DNA samples were annealed by heating at 95 °C for 5 min followed by gradual cooling to 25 °C at a rate of 1 °C/min. The annealed *MET*-G4 DNA was incubated with an equimolar concentration of the purified LRPPRC_1025-1394_ protein in a binding buffer (20 mM Tris-HCl [pH 7.5], 100 mM KCl, 2 mM DTT, 1 mM EDTA, 2 mM MgCl_2_) on ice for 1 h. The final concentration of DNA was 20 μM. CD spectra were subsequently acquired as described above. The CD spectrum for the LRPPRC protein itself was then subtracted from the composite CD spectra of the *MET*-G4 DNA-protein complexes.

### DNA polymerase stop assay

The 5′-end FAM labeled primer (5′-FAM-TAATACGACTCACTATAGCAATTGC) was mixed with template DNA(5′-TAGTGCTGCCTGCGGGCGGGCGGGGCGCTGGGCTCAAGATAGCTGCACGCAATTGCTATAGTGAGTCGTATTA-3′) at a 1.2:1 equivalent. The mixtures were annealed by heating to 95 °C for 5 min then gradually cooling to room temperature in a heating block. Where specified, Nitidine at various concentrations were added. Primer extension was conducted for 3 min in a 50 μL reaction buffer containing 0.12 μM DNA mixtures, 50 μM dNTP, 0.5 U/μL Klenow Fragment (exo-) DNA polymerase (Thermo Fisher Scientific), 5 mM K^+^ (pH 7.0), and 10 mM MgCl_2_ at 37 °C. The reaction was terminated by adding 1/20 volume of 10 mM EDTA-Na_2_ solution. After phenol chloroform extraction and ethanol precipitation, the DNA products were resuspended in 80% (vol/vol) deionized formamide in water, denatured at 95 °C for 5 min and then electrophoresed on a 12% denaturing polyacrylamide gel. Visualization of DNA fragments was performed on GelDoc Go imaging system (Bio-Rad, USA). Image processing and quantification were carried out by Image Lab 6.1 and Imagequant 5.2 software, respectively.

### FRET-melting assay

DNA oligonucleotides for FRET-melting assay were dual-labeled with FAM (6-carboxyfluorescein) at the 5ʹ-end and TAMRA (6-carboxytetramethylrhodamine) at the 3ʹ-end and dissolved in 10 mM K^+^-containing solution. DNA annealing was performed by heating to 95 °C for 5 min followed by gradual cooling to 25 °C at a rate of 1 °C /min. Subsequently, natural compounds were diluted to a final concentration of 10 μM and incubated with 0.2 μM DNA for 3 h at room temperature. Fluorescence melting curves were determined by using a LightCycler 480 Real-Time PCR System (Roche, Switzerland). After a equilibration step at 25 °C for 5 min, a stepwise increase of 0.5 °C every minute was performed to reach 95 °C. Fluorescence signals were recorded with the detection format of mono color hydrolysis. Melting curves were normalized to a scale of 0-1, and the melting temperature (*T*_m_) was defined as the midpoint of the transition curve, corresponding to the temperature at which the normalized fluorescence intensity reached 0.5.

### Electrophoretic mobility-shift assays (EMSA)

DNA samples were dissolved in a 50 mM K^+^-containing buffer and annealed by heating to 95 °C for 5 min then gradually colling down to room temperature in a heating block. Native polyacrylamide gels (16% acrylamide, acrylamide: bisacrylamide 29:1) were cast with dimensions of 10 × 7 cm (1.0 mm thickness) supplemented with 12.5 mM KCl. Each well was loaded with 5 μL of 100 μM DNA mixed with 3 μL of 60% (vol/vol) glycerol. Electrophoresis was conducted in 1 × TBE running buffer at a constant voltage of 45 V. DNA bands were visualized using UV light at 254 nm.

For protein-DNA binding, 100 nM FAM-labeled DNA was incubated with different concentrations of protein in a binding buffer containing 20 mM Tris-HCl (pH 7.5), 100 mM KCl, 2 mM DTT, 1 mM EDTA, and 2 mM MgCl_2_ for 1 h. The samples were then loaded onto an 8% native polyacrylamide gel in 1 × TBE buffer at 4 °C. Electrophoresis was carried out at 4 °C with a constant voltage of 100 V for 50 min. Gels were imaged with GelDoc Go imaging system (Bio-Rad, USA).

For the competitive electrophoretic mobility shift assay (EMSA), binding reactions were performed by incubating 100 nM FAM-labeled DNA, 10 μM unlabeled competitor DNA (*MET*-G4, *MYC*-G4, Bom17-G4, *Tel-hybrid1*-G4, or *Tel-hybrid2*-G4), and 2 μM LRPPRC_1025-1394_ protein in a buffer containing 20 mM Tris-HCl (pH 7.5), 100 mM KCl, 2 mM DTT, 1 mM EDTA, and 2 mM MgCl_2_. The mixture was kept at room temperature for 1 h. Subsequently, the samples were loaded onto an 8% native polyacrylamide gel prepared in 1 × TBE buffer. Electrophoresis was conducted at 4 °C under a constant voltage of 100 V for 50 min. Finally, the gels were visualized using a GelDoc Go imaging system (Bio-Rad, USA).

### Fluorescence measurement

Fluorescence measurements were performed on an RF6000 spectrofluorometer using a 1 cm path length quartz cuvette. Emission spectra (505–600 nm) were recorded with excitation at 492 nm. Experiments employed 50 nM FAM-labeled DNA in 50 mM K^+^ containing solution and NIT at the desired concentration was then added. Fluorescence spectra were collected after 2 min incubation at each time. The *K*_d_ value was derived by nonlinear regression in GraphPad Prism 9: *F* = *F*_min_ + (*F*_max_ – *F*_min_) [(2*D*_T_ + *C*_T_ + *K*_d_) – ((2*D*_T_ + *C*_T_ + *K*_d_)^2^ – (8*D*_T_*C*_T_))^1/2^]/(2*C*_T_), where F represents the ligand-induced fluorescence intensity, C_T_ is the ligand concentration, and D_T_ is the DNA concentration.

### RNA extraction and RT-qPCR assay

Total RNA from HepG2, HCCLM3 cells or HepG2-derived xenografts treated with varying concentrations of Nitidine were extracted using an RNA extraction kit (Shanghaiyishan, China) as described by the manufacturer. RNA purity and concentration were determined by a NanoDrop spectrophotometer (Thermo Scientific, USA). cDNA was synthesized with 1 μg total RNA using HiScript® II Q Select RT SuperMix (Vazyme Biotech, China). Quantitative PCR amplifications were then performed using ChamQ Universal SYBR qPCR Master Mix (Vazyme Biotech, China) according to the manufacturer’s instructions on a LightCycler 480 Real-Time PCR System (Roche). GAPDH expression level was used as an endogenous control for data normalization. The specificity of primer sequences (Table S1) was confirmed by melting curve analysis. Relative gene expression was calculated using the ΔΔCt method.

### Pull-down

Total protein in HepG2 cells was subjected to DNA pull-down assays by biotin-*MET*-G4 (wild-type G-quadruplex-forming sequence), biotin-*MET*-MUT-G4 (mutant control), and biotin-only (negative control). The DNA-bound protein complexes were separated by 10% SDS-PAGE and visualized by Coomassie Brilliant Blue staining. Differential protein bands were excised for mass spectrometry analysis.

### Mass spectrometric (MS) protein identification

Mass spectrometry analysis was carried out via protein reduction alkylation, enzymatic hydrolysis, and peptide desalting with the assistance of Beijing Novogene Co., Ltd. (Beijing, China). In brief, the gel band was cut into pieces and Coomassie brilliant blue (CBB) dye was removed with 50% acetonitrile (ACN)/50 mM ammonium bicarbonate. Gel pieces were dehydrated with 100% ACN and reconstituted in 25 mM ammonium bicarbonate containing 12.5 ng/μL sequencing-grade trypsin (Promega, V5113) to digest proteins at 37 °C overnight. The tryptic peptides were extracted from the gel pieces with 50 % ACN/0.1 % TFA and lyophilized by vacuum centrifugation.

LC-MS/MS analysis were performed on an EASY-nLC 1000 system (Thermo Fisher Scientific, Waltham, MA) connected to an Orbitrap Fusion mass spectrometer (Thermo Fisher Scientific, San Jose, CA) equipped with an online nano-electrospray ion source. The peptide was resuspended with 10 μL solvent A (water with 0.1% formic acid) and loaded onto the trap column (Thermo Scientific Acclaim PepMap C18, 100 μm × 2 cm) with a flow of 10 μL/min for 3 min and subsequently separated on the analytical column (Acclaim PepMap C18, 75 μm × 25 cm) with a linear gradient. The gradient started from 2% B (90% acetonitrile, 0.1% formic acid in water) to 35% B in 75 min, 35% to 60% in 10 min, 60% to 100% in 5 min. The column flow rate was maintained at 300 nL/min and column temperature was maintained at 40 °C.

The Orbitrap Fusion mass spectrometer was operated in the data-dependent mode to switch automatically between MS and MS/MS acquisition. Survey full-scan MS spectra (m/z 350-1600) were acquired in Orbitrap with a mass resolution of 120000, the AGC target was set to 1000000, and the maximum injection time was 50 ms (millisecond). Precursor ions with charge states 2 + , 3 + , and 4+ were sequentially fragmented by higher energy collisional dissociation (HCD) with a normalized collision energy (NCE) of 30% to generate fragment ions (MS/MS spectra). MS/MS acquisition was performed in top speed mode with 3 s cycle time in Orbitrap with the resolution of 15000, the maximum injection time was 100 ms, the AGC target was set to 100000, and the isolation window was 1.6 m/z. In all cases, one microscan was recorded using dynamic exclusion of 30 s.

The raw mass files generated by the Orbitrap Fusion were processed using MaxQuant software (version 2.0.2.0, https://www.maxquant.org/) for protein identification. MS and MS/MS spectra were searched using the Andromeda search engine against the *human* Uniprot database (Proteome ID: UP000005640, 20371 entries). The parameters for database search were set as follows: (1) The minimum required peptide length was 7 amino acids. (2) Trypsin cleavage specificity was applied with up to two missed cleavages allowed. (3) Acetyl (Protein N-term) and oxidation (M) were set as variable modifications. (4) The mass tolerance for precursor and fragment ions were set 20 ppm and 0.5 Da, respectively. (5) The false discovery rate (FDR) was set to 1% at both the peptide and protein levels. The MaxQuant-outputted files was uploaded into Perseus software (version 1.6.15.0, https://www.maxquant.org/perseus/) to perform analysis.

### Microscale thermophoresis (MST)

MST experiments were performed on a Monolith NT.115 instrument (NanoTemper Technologies). The FAM-labeled DNA was dissolved in a 100 mM K^+^-containing buffer (pH 7.5) and annealed, followed by gradual cooling to 25 °C at a rate of 1 °C /min. To determine the *K*_d_ values, 100 nM 5*ʹ*-FAM-labeled DNA was incubated with increasing concentrations of LRPPRC_1025-1394_ protein for 1 h at 25 °C. The MST data was then collected at 20% blue LED excitation power and high MST-power. Normalized fluorescence values at a given time were obtained. Data was analyzed and *K*_d_ values were generated using the Nano Temper analysis software. Graphs were further plotted in GraphPad Prism 9.0 for visualization.

### Bio-layer interferometry (BLI) analysis

Binding kinetics between nitidine and LRPPRC_1025-1394_ were quantified via BLI using Octet RED96 (ForteBio). Serial dilutions of NIT (1.5625-100 μM) in kinetic buffer (1 × PBS, 0.01% DMSO) were prepared for dose-response profiling. Recombinant His-tagged LRPPRC_1025-1394_ was immobilized onto Ni-NTA biosensor tips (ForteBio, Menlo Park, CA), with protein-free buffer controls (PBS + 0.01% DMSO) establishing baseline drift correction. Assays were performed according to a standard protocol with a total volume of 200 μL per well in 96-well black plates at 30 °C. Raw binding curves were processed through the Octet Data Analysis software (Sartorius) using a double reference subtraction method.

### Drug affinity responsive target stability (DARTS)

HepG2 cells were lysed in ice-cold NP-40 buffer (Sigma-Aldrich). Total protein extracts were subjected to three cycles of ultrasonic disruption (30% amplitude, 3 s pulse/4 s interval) followed by centrifugation at 18,000 × *g* for 10 min at 4 °C. Proteins were quantified via BCA assay, with equivalent aliquots (100 μg/sample) from three biological replicates processed under parallel conditions. Protein samples were pre-incubated for 1 h at 37 °C with: DMSO control, 2 μM nitidine, 1 μM *MET*-G4, NIT-stabilized *MET*-G4 complex (2 μM NIT + 1 μM *MET*-G4). Pronase was added simultaneously to all samples and incubated at 37 °C for 30 min, then the protease inhibitor cocktail was added to stop reactions, and SDS-PAGE was carried out to analyze the protein stability.

### DNA pull-down and western blotting

3*ʹ*-biotinylated oligonucleotides were purchased from Sangon Biotech (Shanghai) with HPLC purification service. The G4-forming oligonucleotide was folded by heating at 95 °C for 5 min in a buffer containing 10 mM Tris (pH 7.5) and 100 mM KCl, followed by gradual cooling to 25 °C at a rate of 1 °C /min. Successful folding was confirmed by circular dichroism (CD) spectroscopy using a Jasco-1500 spectropolarimeter (Jasco Inc., Japan).

Total cellular proteins were extracted from HepG2 cells lysed with RIPA lysis buffer supplemented with 1% PMSF and centrifuged at 12,000 × *g* for 10 min. Lysates were clarified by centrifugation at 12,000 × *g* for 10 min at 4 °C, and protein concentrations were determined using a bicinchoninic acid (BCA) assay kit (Beyotime Biotechnology, China).

For DNA pull-down experiments, 1 mg of total protein was incubated with biotin, biotin-*MET*-G4, or biotin-*MET*-MUT-G4 overnight at 4 °C, and then the complexes were incubated with Avidin Agarose for 4 h at 4 °C with gentle rotation. After five washes with ice-cold phosphate-buffered saline (PBS), bound proteins were eluted by boiling in 2× SDS loading buffer at 95 °C for 10 min.

For western blotting analysis, equal amounts of total proteins or DNA-protein complexes were separated by SDS-PAGE gels alongside a pre-stained protein ladder (Yamei Tricolor Prestained Protein Marker, 10–250 kDa, Cat# WJ103) and then transferred to PVDF membranes (Millipore, USA). Then the membranes were blocked with 5% skim milk for 2 h at room temperature and immunoblotted with primary antibodies overnight at 4 °C, followed by incubating with an appropriate (HRP)-conjugated secondary antibody for 1 h at room temperature. Protein bands were visualized using enhanced chemiluminescence (ECL) reagents (Vazyme Biotech, China). Antibody details are provided in Table S6.

### Dual-luciferase reporter assay

HepG2 cells were seeded at a density of 2 × 10^5^ cells per well in 12-well plates and transfected with WT- or MUT-*MET*-G4 luciferase reporter plasmids using Lipofectamine 3000 (Thermo Fisher Scientific, USA) according to the manufacturer’s protocol. Subsequently, the cells were collected using cell lysis buffer and subjected to analysis for firefly and renilla luciferase activities using the dual-luciferase reporter assay kit (Vazyme, DL101). Relative luciferase activity was calculated as the ratio of Renilla luciferase activity to firefly luciferase activity.

### Protein expression and purification

The coding sequence (CDS) of human LRPPRC was obtained from the National Center for Biotechnology Information (NCBI) database. The successfully constructed recombinant expression vectors were transformed into *E. coli* BL21(DE3) competent cells. Positive colonies were selected and cultured in Luria-Bertani (LB) liquid medium with the corresponding antibiotic at 37 °C. When OD_600_ reached approximately 0.6, the culture was cooled to 16 °C, and the protein expression was induced with 0.5 mM isopropyl 1-thio-*β*-D-galactopyranoside (IPTG). After 16 h of induction, cells were harvested and lysed. After centrifugation at 12,000 × *g* for 30 min at 4 °C, the lysate supernatant was collected. Proteins were purified and enriched using BeyoGold™ His-Tag Purification Resin (Beyotime, China) following the manufacturer’s recommended procedures. Further purification was performed using an ÄKTA pure protein purification system (Cytiva, USA) with sequential ion-exchange chromatography and size-exclusion chromatography. Protein purity was assessed by SDS-PAGE, and concentrations were determined using a bicinchoninic acid (BCA) assay kit (Beyotime Biotechnology, China).

### G4-CUT&Tag

The cleavage under targets and tagmentation (CUT&Tag) assay was performed according to the manufacturer’s protocol (Hunan Ruoyu Biotech, China). In brief, harvested HepG2 cells were immobilized on concanavalin A (ConA)-conjugated magnetic beads. The beads were then incubated with a G4P (250 nM) for 2 h at room temperature. After washing, an anti-FLAG primary antibody (F1804, Sigma-Aldrich; 1:100 dilution) was added and incubated for 1 h at room temperature. Subsequently, a secondary antibody [goat anti-mouse IgG (AP124, Sigma-Aldrich); 1:100 dilution] was applied and incubated for 2 h at 25 °C with gentle shaking. Following antibody binding, DNA was cleaved and released. The released DNA fragments were purified, amplified, and further purified according to the kit instructions. Each experiment was performed in duplicate.

For sequencing data analysis, reads were aligned to the human reference genome (hg38) using Bowtie2 (v2.3.5.1) with the--fast-local parameter. Coverage tracks in bigWig format were generated with deepTools (v3.3.2) bamCoverage using the following settings: --binSize 50 --extendReads --ignoreDuplicates.

### Chromatin immunoprecipitation (ChIP)

HepG2 Cells were cultured in DMEM medium with or without 2 μM nitidine for 6 h before cross-linking. Approximately 3 × 10^6^ cells were cross-linked with 1% (vol/vol) formaldehyde at room temperature for 10 min with gentle rotation. The reaction was quenched by adding glycine to a final concentration of 125 mM and incubating at 25 °C for 5 min with rotation. After washing with cold PBS twice, the cells were resuspended in cell lysis buffer (5 mM PIPES, pH 8.0, 85 mM KCl, 0.5% NP-40, protease inhibitors cocktail) at 4 °C for 10 min. After centrifugation at 1500 × *g* at 4 °C for 5 min, the pellet was resuspended in nuclear lysis buffer (50 mM Tris-HCl, pH 8.1, 1% SDS, 10 mM EDTA, protease inhibitor cocktail) at 4 °C for 30 min. The Chromatin was sheared to a size range of 200–500 bp by sonication at 4 °C with the following parameters: 30% power output, 10 s on and 10 s off for a total duration of 10 min. The shearing size of the chromatin was usually 200–500 bp, and the shearing efficiency was verified by 1% agarose gel electrophoresis. After centrifugation at 12,000 × *g* at 4 °C for 10 min, the supernatant was collected and incubated with 3 μg anti-LRPPRC antibody (Abcam, ab259927) at 4 °C overnight with rotation. Subsequently, the mixture was added to Protein A/G Magnetic Beads (MCE, HY-K0202) and incubated at 4 °C for 4 h. After washing with ice-cold PBS for 5 times, cross-links were reversed by adding 8 μL 5 M NaCl and incubating at 65 °C for 4 h. RNA was removed with RNase A at 37 °C for 30 min. DNA was subsequently eluted from the beads with 100 mM NaHCO_3_ and 1% SDS at 68 °C for 2 h.

For G4 ChIP, chromatin was incubated with 3 μg Biotin-G4P at 16 °C for 2 h. To the mixture were added Streptavidin Magnetic Beads (Beyotime Biotechnology, P2151), and the mixture was incubated at 16 °C for 1 h with rotation. After washing with washing buffer (10 mM Tris–HCl, pH 8.0, 0.1% Tween-20, 100 mM KCl) for 5 times, DNA was eluted from the beads with TE buffer at 37 °C for 1 h. Cross-links were subsequently reversed b at 65 °C for 2 h, and RNA was removed with RNase A at 37 °C for 30 min. Finally, the DNA was purified using FastPure Gel DNA Extraction Kit (Vazyme, DC301) according to the manufacturer’s protocol.

### Knockdown, overexpression, and transfection

Knockdown of LRPPRC or MET was achieved via transient transfection with small interfering RNA (siRNA) using Lipofectamine 3000 (Thermo Fisher Scientific, USA) in Opti-MEM for 24 to 48 h according to the manufacturer’s instructions. The sequences of siRNA are listed in Table S1. A non-targeting scrambled siRNA was used as a negative control.

For LRPPRC overexpression, the coding sequence (CDS) of human LRPPRC was obtained from the NCBI database and cloned into the expression vector pcDNA3.4. Overexpression was subsequently performed via transient transfection, as described in the previous section. In each transfection experiment using a 6-well plate, 2 µg of plasmid DNA was used.

### Immunofluorescence

LRPPRC overexpression was induced as described. Following overnight seeding and adherence, DNA transfection was carried out using Cy5-labeled oligonucleotides with Lipofectamine 3000 Transfection Reagent in Opti-MEM medium for 6 h, after which the medium was aspirated. For nitidine treatment, cells were further incubated with the compound at specified concentrations for 24 h. Subsequently, cells were fixed with 4% paraformaldehyde for 10 min at room temperature, followed by PBS rinsing and permeabilization in 1% Triton X-100/PBS at 37 °C for 15 min. After additional PBS washes, samples were blocked with 3% BSA/PBS at 37 °C for 60 min and then incubated with FAM-G4P overnight at 4 °C. Following this, nuclei were stained with DAPI for 10 min. After three final PBS washes, digital images were acquired using an FV4000 laser scanning confocal microscope (Olympus) equipped with a 60× objective, and analyzed with ImageJ software.

### Cell cycle assays

HepG2 and HCCLM3 cells were seeded at a density of 3 × 10^5^ cells per well in 6-well plates and treated with varying concentrations of nitidine for 24 h, then cells were fixed with 75% ethanol for 12 h at 4 °C and incubated with 500 μL Propidium Iodide (PI) for 30 min in the dark, the cell cycle distribution was determined using a flow cytometry (BD Biosciences, USA) with excitation at 488 nm and emission detection at 617 nm.

### Cell apoptosis assays

HepG2 and HCCLM3 cells were seeded at a density of 3 × 10^5^ cells per well in 6-well plates and exposed to varying concentrations of nitidine for 48 h. Subsequently, the cells were incubated with 10 μL Annexin-V conjugated FITC and 5 μL PI for 15 min in the dark. Apoptosis was determined using flow cytometry (BD Biosciences, USA).

### Animal experiments

4-week-old male Balb/c nude mice were subcutaneously injected with 4 × 10^6^ HepG2 cells or 5 × 10^6^ H1993 cells into the flank region. Once the tumor volume reached approximately 100 mm^3^, the mice were randomly allocated into different treatment groups, including vehicle control, sorafenib (30 mg/kg/day), capmatinib (10 mg/kg/day) and nitidine at different doses (2.5 mg/kg/day, 5 mg/kg/day, and 10 mg/kg/day). Following 19 consecutive days of treatment, the blood samples of mice were collected for biochemistry analysis. Subsequently, the mice were euthanized, and tumor tissues were harvested for further analysis of gene and protein expression, as well as histological examination.

### Serum biochemistry

Serum was isolated from blood samples by centrifugation at 4 °C for 10 min. The serum levels of alanine aminotransferase (ALT) and aspartate aminotransferase (AST) were quantified using standard enzymic procedures according to the manufacturer’s instructions.

### Histology and Immunohistochemical (IHC) analysis

Tumor tissues were fixed in paraformaldehyde solution and embedded in paraffin. Tissue sections were stained with haematoxylin-eosin (H&E) to assess tumor morphology and pathological damage. The Ki67 staining was used to evaluate the proliferation index of tumor cells, and the IHC staining of MET was used to determine MET protein expression levels.

### Statistical analysis

All data are presented as the mean ± SEM values and analyzed by GraphPad Prism. Statistical significances were analyzed using Student’s *t*-test, one-way analysis of variance (ANOVA), or two-way analysis of variance (ANOVA). *p*-values are provided in the figure legends.

### Reporting summary

Further information on research design is available in the Nature Portfolio Reporting Summary linked to this article.

## Supplementary information

Supplementary Information

Reporting Summary

Transparent Peer Review file

## Source data

Source Data

## Data Availability

The data underlying this article are available in the Protein Data Bank (PDB), and can be accessed with ID 9JI9 10.2210/pdb9JI9/pdb. MS data deposited in the PRIDE database with accession PXD068846. CUT&Tag data have been deposited in the Gene Expression Omnibus (GEO) under accession GSE330177. All other data are available in the article or Supplementary information. Source data are provided with this paper.

## Code availability

No custom code was generated in this study.
