# Supplementary material for: Molecular glue that stabilizes the LRPPRC−MET-G4 interaction complex to drive MET downregulation
Source: Nat Commun. 2026 Jun 4;17:7184. doi: 10.1038/s41467-026-73806-6 (PMC13396614; doi:10.1038/s41467-026-73806-6)
Supplement: Supplementary file 1 — Supplementary Information [file 41467_2026_73806_MOESM1_ESM.pdf]

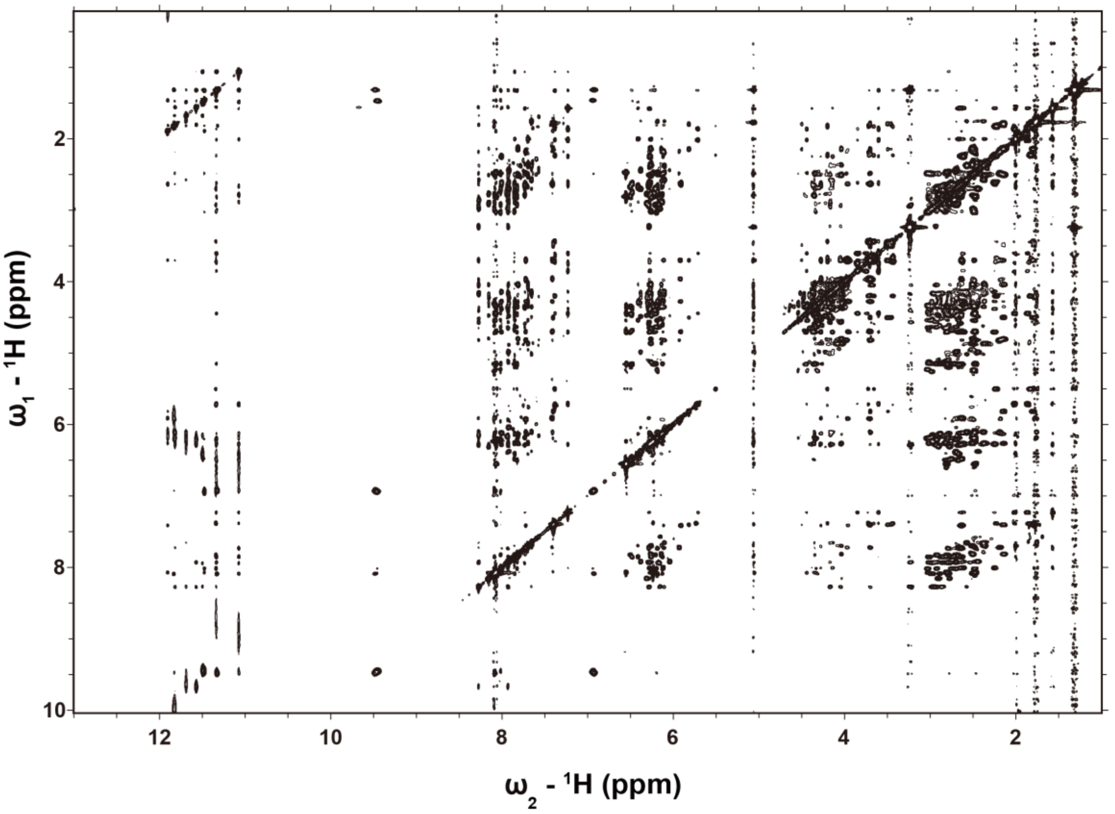

Supplementary Figure 1 | Full 2D-NOESY spectrum of pu25m1T. The H1-H1 region (10-12 ppm) is shown as a fold-back spectrum. Conditions: 1.51 mM DNA, pH 7.0, 25 mM K^+, 15 °C.

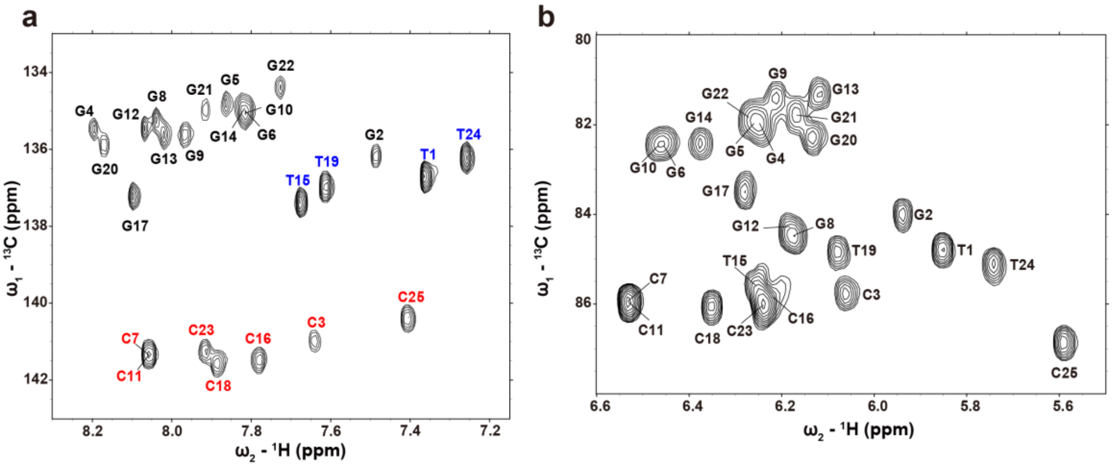

Supplementary Figure 2 | ^{1}H-^{13}C HSQC resonance assignments. **a**, H8-C8 cross-peaks for guanine residues (labeled in black), H6-C6 contacts for cytosine residues (labeled in red), and H6-C6 contacts for thymine residues (labeled in blue) with assignments of Pu25m1T DNA by ^{1}H-^{13}C HSQC experiments. **b**, H1′-C1′ cross-peaks for all bases with assignments of Pu25m1T DNA by ^{1}H- ^{13}C HSQC experiments. Conditions: 1.51 mM DNA, pH 7.0, 25 mM K^{+}, 25 °C. Source data are provided as a Source Data file.

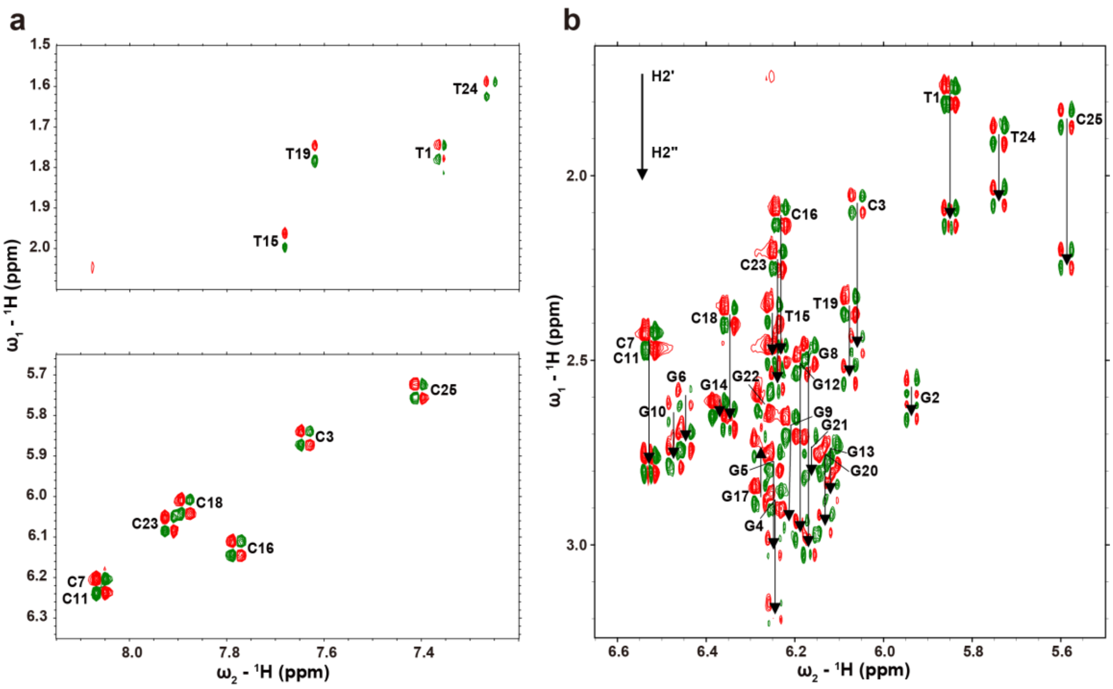

Supplementary Figure 3 | DQF-COSY resonance assignments. a, The thymine H6-methyl (top) and cytosine H5-H6 (bottom) cross-peaks with assignments of Pu25m1T DNA by DQF-COSY experiments. **b**, The H1′-H2′ and H1′-H2″ intraresidual cross-peaks for all bases with assignments of Pu25m1T DNA by DQF-COSY experiments. The assignments of H2′/H2″ are indicated by the direction of the arrows. Conditions: 1.51 mM DNA, pH 7.0, 25 mM K^+, 25 °C. Source data are provided as a Source Data file.

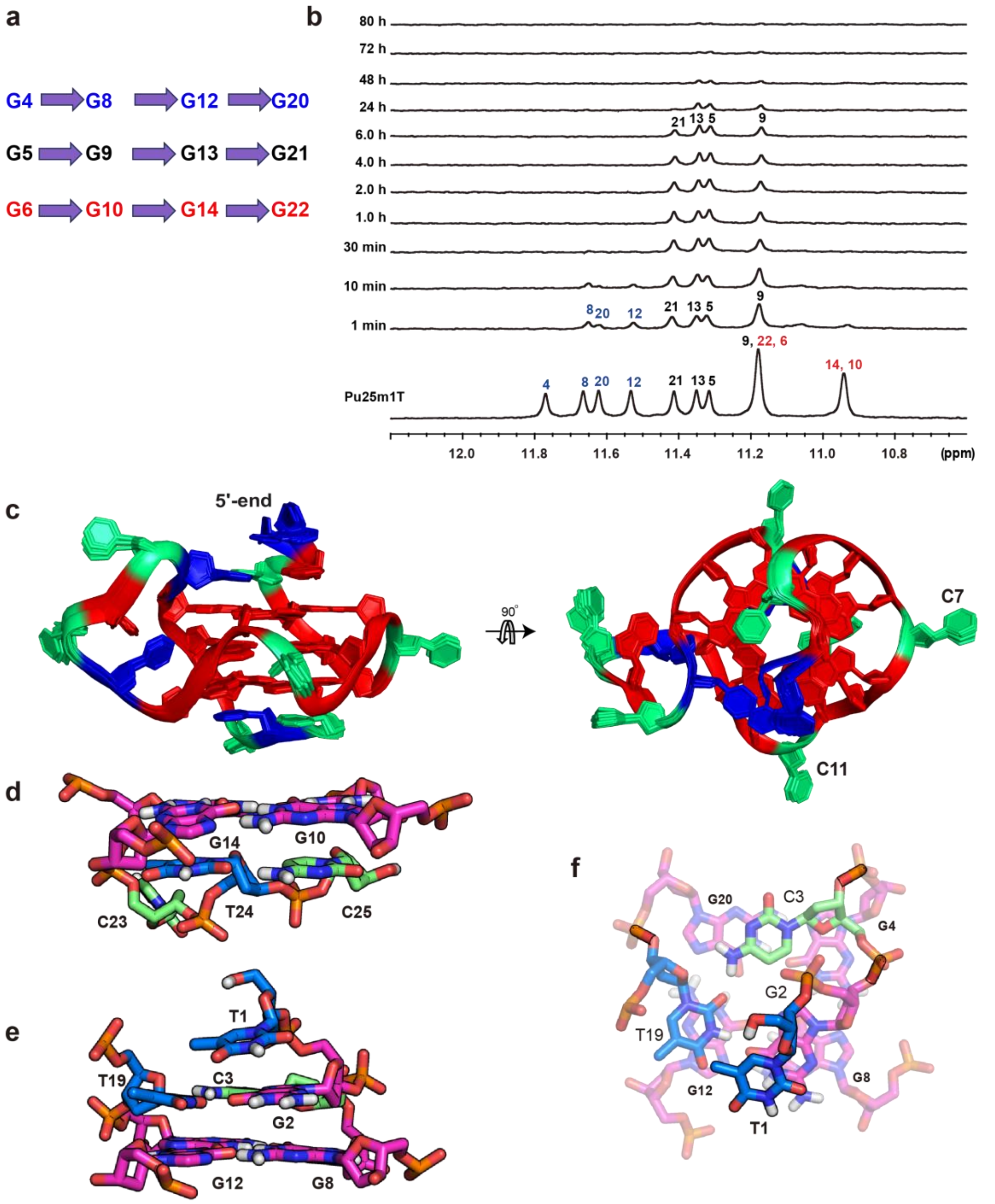

Supplementary Figure 4 | G-tetrads core determination and high-resolution NMR solution structure of the *MET* proximal promoter G-quadruplex. **a**, The assigned three G-tetrad planes of *MET*-G4 by NMR experiments. **b**, Solvent exchange experiments of Pu25m1T DNA. The imino proton spectra of Pu25m1T DNA in H_2O (bottom) and subsequent dissolution of the sample in D_2O at the indicated time points are presented. Conditions: 150 μM DNA, pH 7.0, 50 mM K^+ solution, 25 °C. **c**, Superposition of the 15 lowest-energy NMR-derived structures of *MET*-G4, shown in side view (left) and top view (right). **d** and **e**, Side views of the 3′-end and 5′-end capping structure of *MET*-G4. **f**, Top view of the 5′-end capping structure of *MET*-G4. Magenta, guanine; green, cytosine; blue, thymine. Source data are provided as a Source Data file.

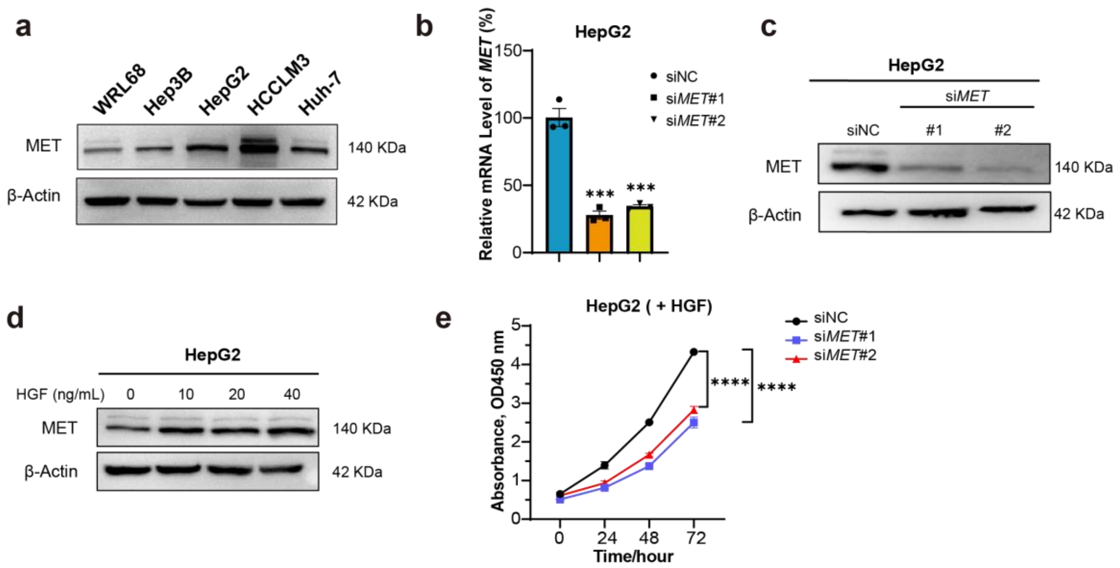

Supplementary Figure 5 | HepG2 cells as a suitable model with functional MET dependency for HGF-induced proliferation. a, MET protein expression levels in WRL68, Hep3B, HepG2, HCCLM3, Huh-7 cells. Three independent experiments were performed with similar results; a representative image is shown. **b** and **c**, MET mRNA and protein expression levels in HepG2 cells with or without *MET* knockdown. RT-qPCR data: Data are presented as mean ± S.E.M. from three independent experiments. Statistical analysis was performed using a two-tailed *t*-test. ***$p = 0.0006$, ***$p = 0.0006$. **d**, The MET protein levels were measured in HepG2 cells stimulated with or without HGF. The experiment was repeated three times independently with similar results; representative images are shown. **e**, Cell proliferation was assessed using the CCK-8 assay in 40 ng/mL HGF-stimulated HepG2 cells with or without *MET* knockdown at the indicated time points. Two-way ANOVA. ****$p < 0.000001$. Source data are provided as a Source Data file.

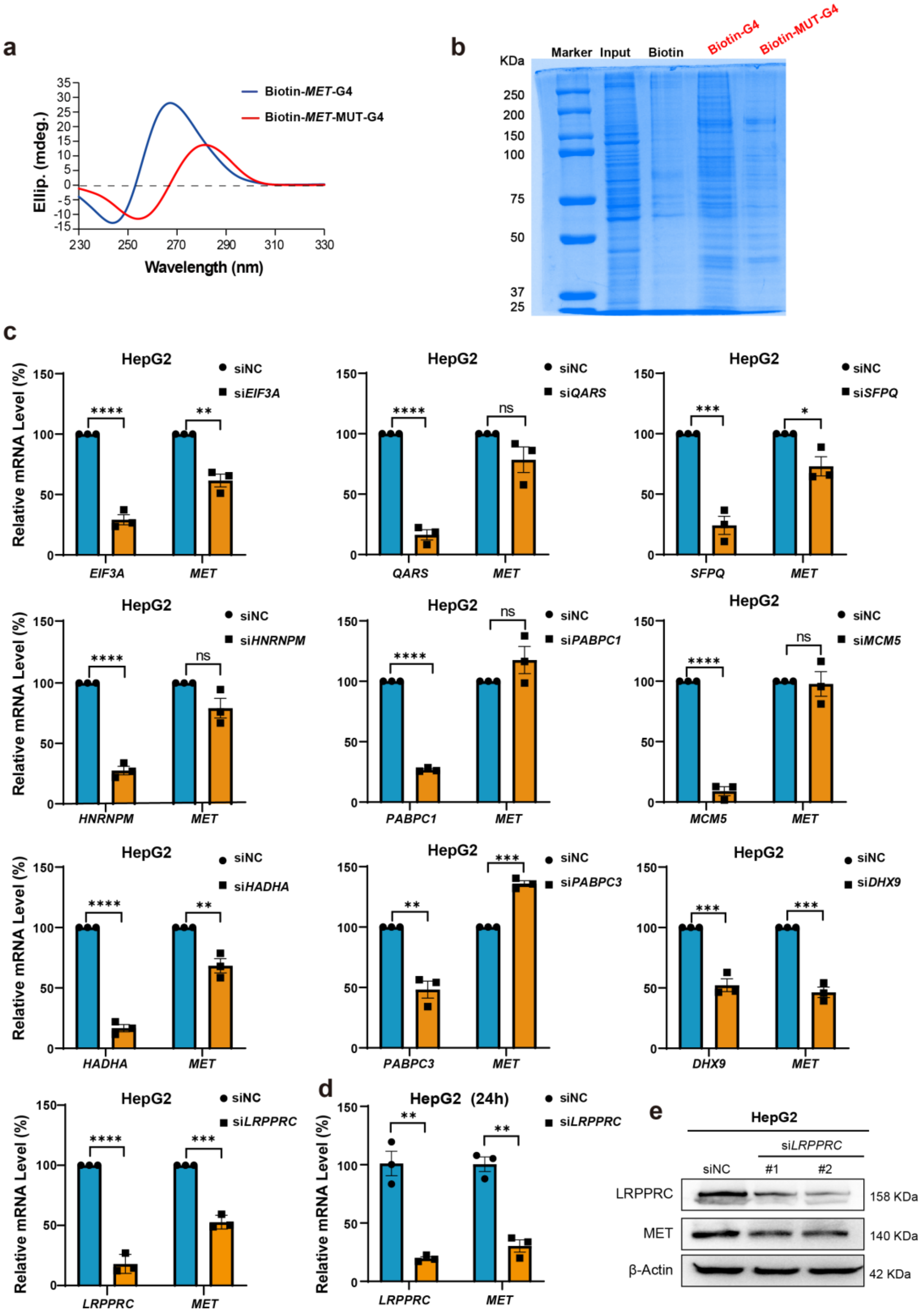

Supplementary Figure 6 | Identification of cellular proteins with potential *MET*-G4 binding activity. a, ECD spectra of the biotin-labeled *MET*-G4 and *MET*-MUT-G4, respectively. Conditions: 100 µM DNA, pH 7.0, 100 mM K^+. **b**, Differential protein bands pulled down from HepG2 cell lysates using biotin-labeled *MET*-G4 (Biotin-G4) and Biotin-MUT-G4 were identified by SDS-PAGE followed by Coomassie blue staining. **c**, The mRNA expression levels of *MET* were measured in HepG2 cells transfected with or without small interfering RNA (siRNA) targeting *EIF3A* (*****p* = 0.000072, ***p* =

0.002), *QARS* (****p = 0.000039, ns: not significant), *SFPQ* (***p = 0.0005, *p = 0.0274), *HNRNPM* (****p = 0.00003, ns: not significant), *PABPC1* (****p = 0.00000017, ns: not significant), *MCM5* (****p = 0.000016, ns: not significant), *HADHA* (****p = 0.00001, **p = 0.0057), *PABPC3* (**p = 0.0019, ***p = 0.0001), *DHX9* (***p = 0.0009, ***p = 0.0002), or *LRPPRC* (****p = 0.000058, ***p = 0.0001) after 48 h. Data are presented as mean ± S.E.M. from three independent experiments. Data were analyzed by a two-tailed *t*-test. **d**, The mRNA expression levels of *MET* were measured in HepG2 cells with or without LRPPRC knockdown (**p = 0.0018, **p = 0.001) for 24 h. **e**, The protein expression levels of MET were measured in HepG2 cells with or without LRPPRC knockdown. Three independent experiments were performed with similar results; representative images are shown. Source data are provided as a Source Data file.

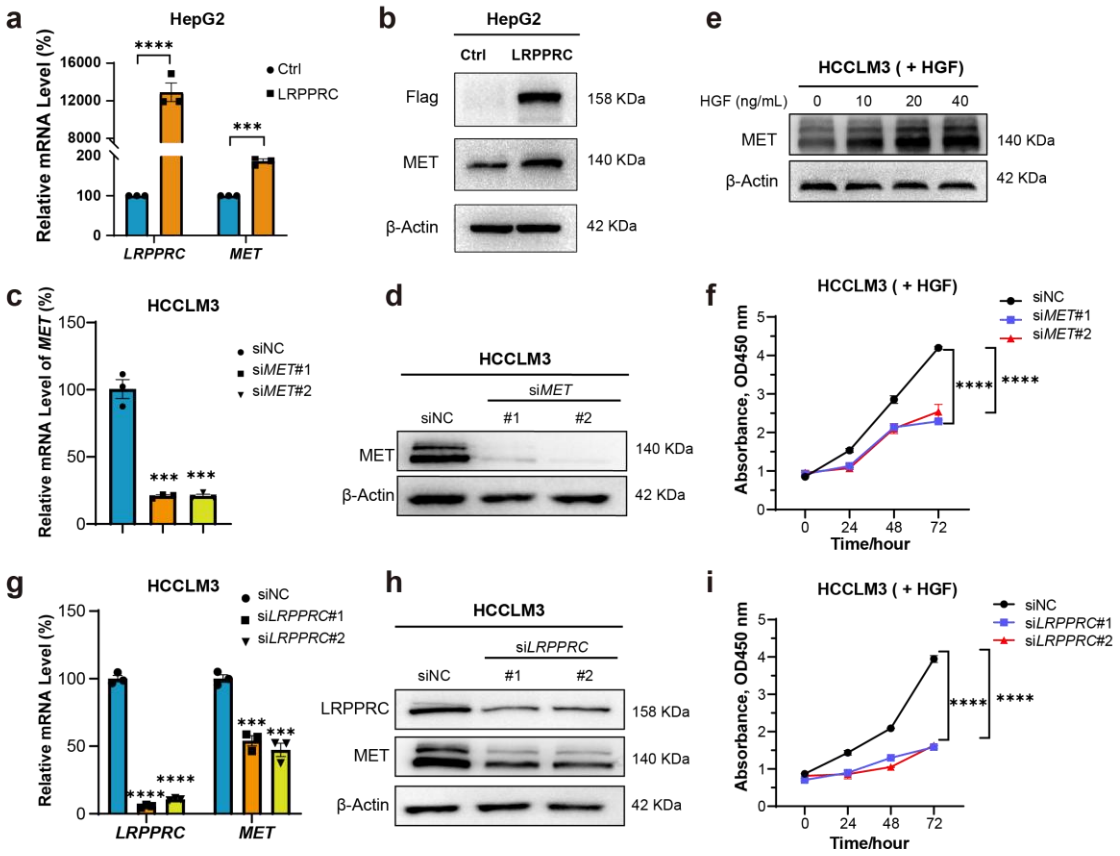

Supplementary Figure 7 | LRPPRC modulates MET expression. a and **b**, MET mRNA and protein levels in HepG2 cells with or without LRPPRC overexpression. RT-qPCR data are presented as mean ± S.E.M. from three independent experiments. Statistical analysis was performed using a two-tailed *t*-test. ****$p < 0.0001$, ***$p = 0.0001$. **c** and **d**, MET mRNA and protein expression levels in HCCLM3 cells with or without *MET* knockdown. RT-qPCR data are presented as mean ± S.E.M. from three independent experiments. Statistical analysis was performed using a two-tailed *t*-test. ***$p = 0.0004$. **e**, MET protein levels in HCCLM3 cells stimulated with or without HGF. The experiment was repeated three times independently with similar results; representative images are shown. **f**, Cell proliferation was assessed using CCK-8 assay in 40 ng/mL HGF-stimulated HCCLM3 cells with or without *MET* knockdown at the indicated time points. Two-way ANOVA. ****$p < 0.000001$. **g** and **h**, MET mRNA and protein expression levels in HCCLM3 cells with or without *LRPPRC* knockdown. RT-qPCR data are presented as mean ± S.E.M. from three independent experiments. Statistical analysis was performed using a two-tailed *t*-test. ***$p = 0.0006$, ***$p = 0.0007$, ****$p < 0.0001$. The mature MET chain is the lower band (~140 kDa) in the representative western blotting images. **i**, Cell proliferation was assessed using CCK-8 assay in 40 ng/mL HGF-stimulated HCCLM3 cells with or without *LRPPRC* knockdown at the indicated time points. $n = 3$, Two-way ANOVA. ****$p < 0.000001$. Source data are provided as a Source Data file.

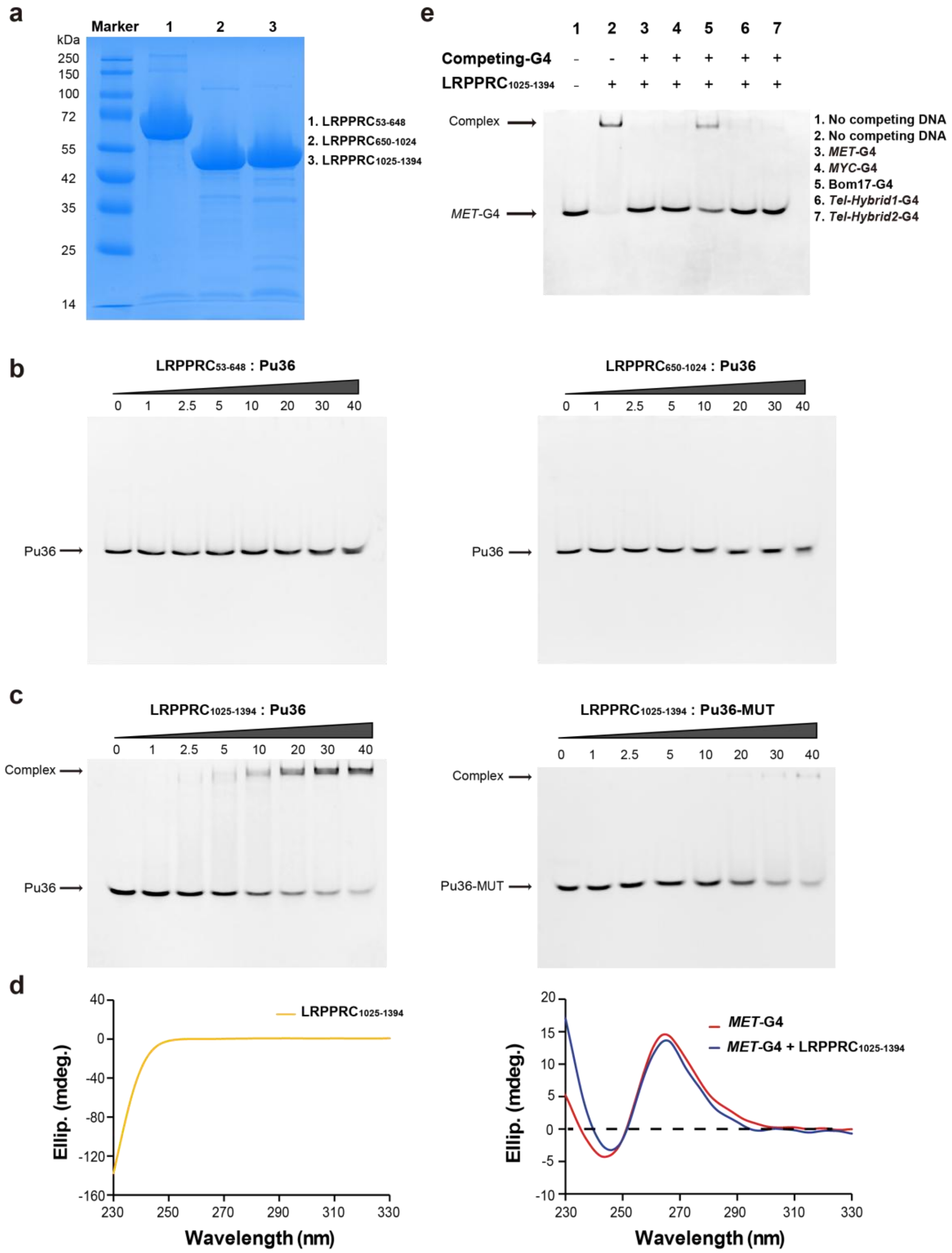

Supplementary Figure 8 | The 1025-1394 Domain of LRPPRC is capable of *MET*-G4 recognition. **a**, Coomassie blue-stained SDS-PAGE analysis of purified LRPPRC truncated fragments: $LRPPRC_{53-648}$, $LRPPRC_{650-1024}$, and $LRPPRC_{1025-1394}$. **b**, Binding of $LRPPRC_{53-648}$ and $LRPPRC_{650-1024}$ to Pu36 DNA. **c**, Binding of $LRPPRC_{1025-1394}$ to Pu36 or Pu36-MUT DNA. Condition: 0.1 μM FAM-labeled DNA, and the corresponding ratios of protein to DNA are labeled. **d**, ECD spectrum of $LRPPRC_{1025-1394}$ protein (left). Comparison of ECD spectra of *MET*-G4 in the presence or absence of $LRPPRC_{1025-1394}$ protein (right). The ECD spectra of the *MET*-G4 in the presence of $LRPPRC_{1025-1394}$ protein were obtained by subtracting the ECD spectrum of the $LRPPRC_{1025-1394}$ protein from the composite spectra of the protein-

G4 DNA complexes. **e**, Competition electrophoretic mobility shift assay (EMSA) of label-free *MET*-G4, *MYC*-G4, Bom17-G4, *Tel-hybrid1*-G4, and *Tel-hybrid2*-G4 binding to $LRPPRC_{1025\text{-}1394}$ protein. [FAM-*MET*-G4] = 0.1 μM, [$LRPPRC_{1025\text{-}1394}$] = 2 μM, competing [oligo] = 10 μM. The experiment was repeated three times independently with similar results; representative images are shown. Source data are provided as a Source Data file.

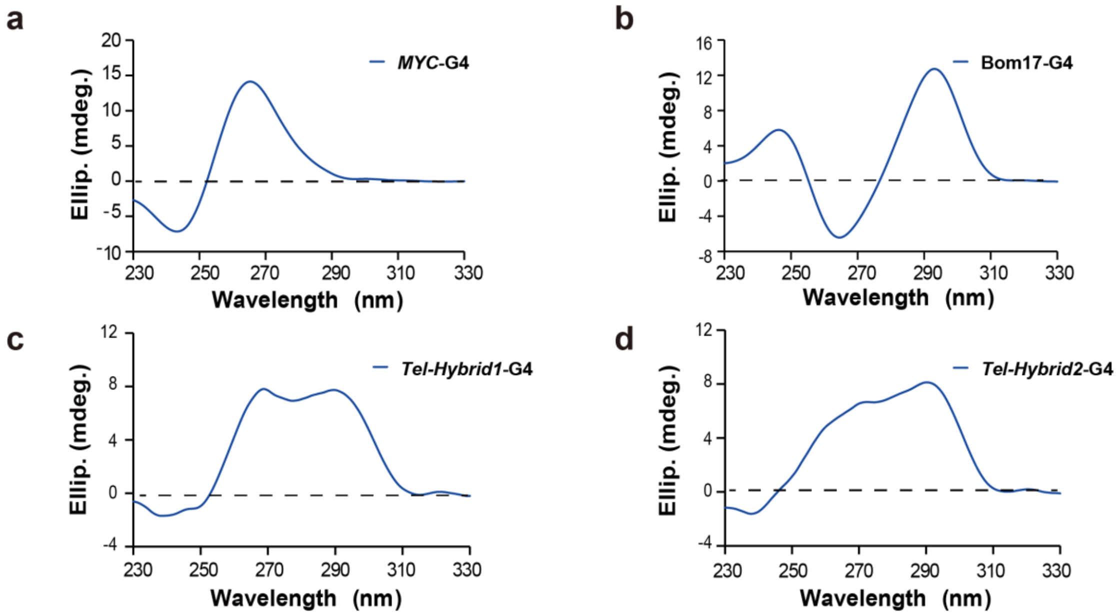

Supplementary Figure 9 | ECD spectra of *MYC*-G4, Bom17-G4, *Tel-hybrid1*-G4 and *Tel-hybrid2*-G4. Conditions: 20 μM DNA, pH 7.0, 50 mM K^+. Source data are provided as a Source Data file.

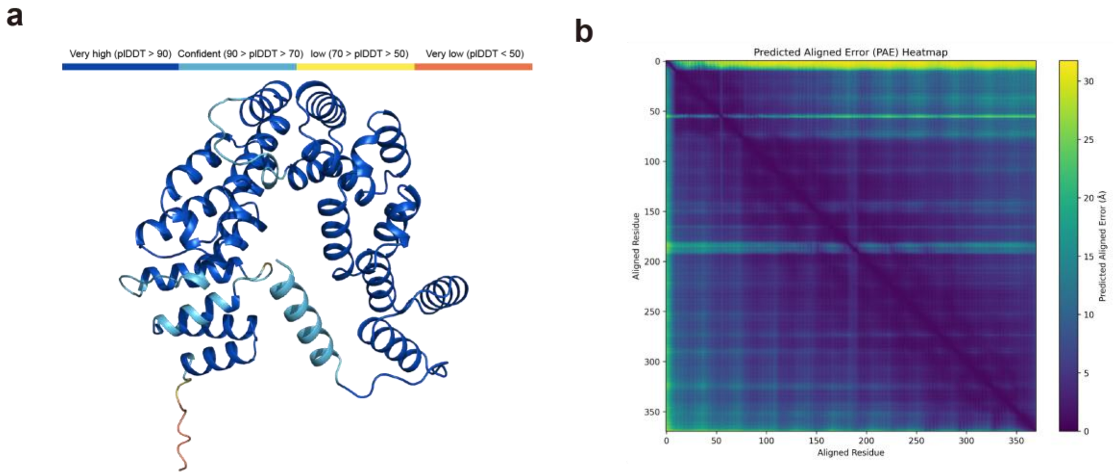

Supplementary Figure 10 | AlphaFold 3-predicted structure of LRPPRC$_{1025\text{-}1394}$ and its confidence metrics. a, The predicted three-dimensional model of the LRPPRC$_{1025–1394}$ generated by the AlphaFold Server (powered by AlphaFold 3) is shown. The model is coloured by the per-residue pLDDT (predicted local distance difference test) confidence score using the official AlphaFold colour scheme: dark blue (pLDDT > 90, very high confidence), light blue (90 > pLDDT > 70, high confidence), yellow (70 > pLDDT > 50, low confidence), and orange (pLDDT < 50, very low confidence). The colour key is indicated above. **b,** The Predicted Aligned Error (PAE) plot for the same model. Each cell (i, j) shows the expected positional error (in Å) when aligning residue i to residue j. Lower PAE values (dark blue) indicate higher relative confidence in the spatial arrangement between two residues. The plot is coloured using the alphafold palette.

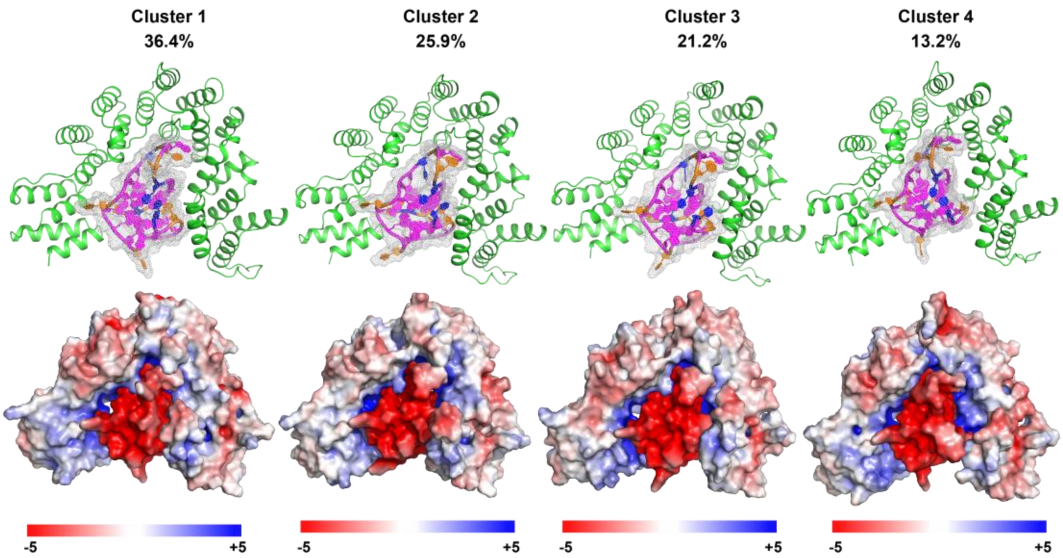

Supplementary Figure 11 | Four representative clustering structures derived from the MD clustering analysis. Cartoon representation (top) and electrostatic potential energy map (bottom) of four representative clustering structures.

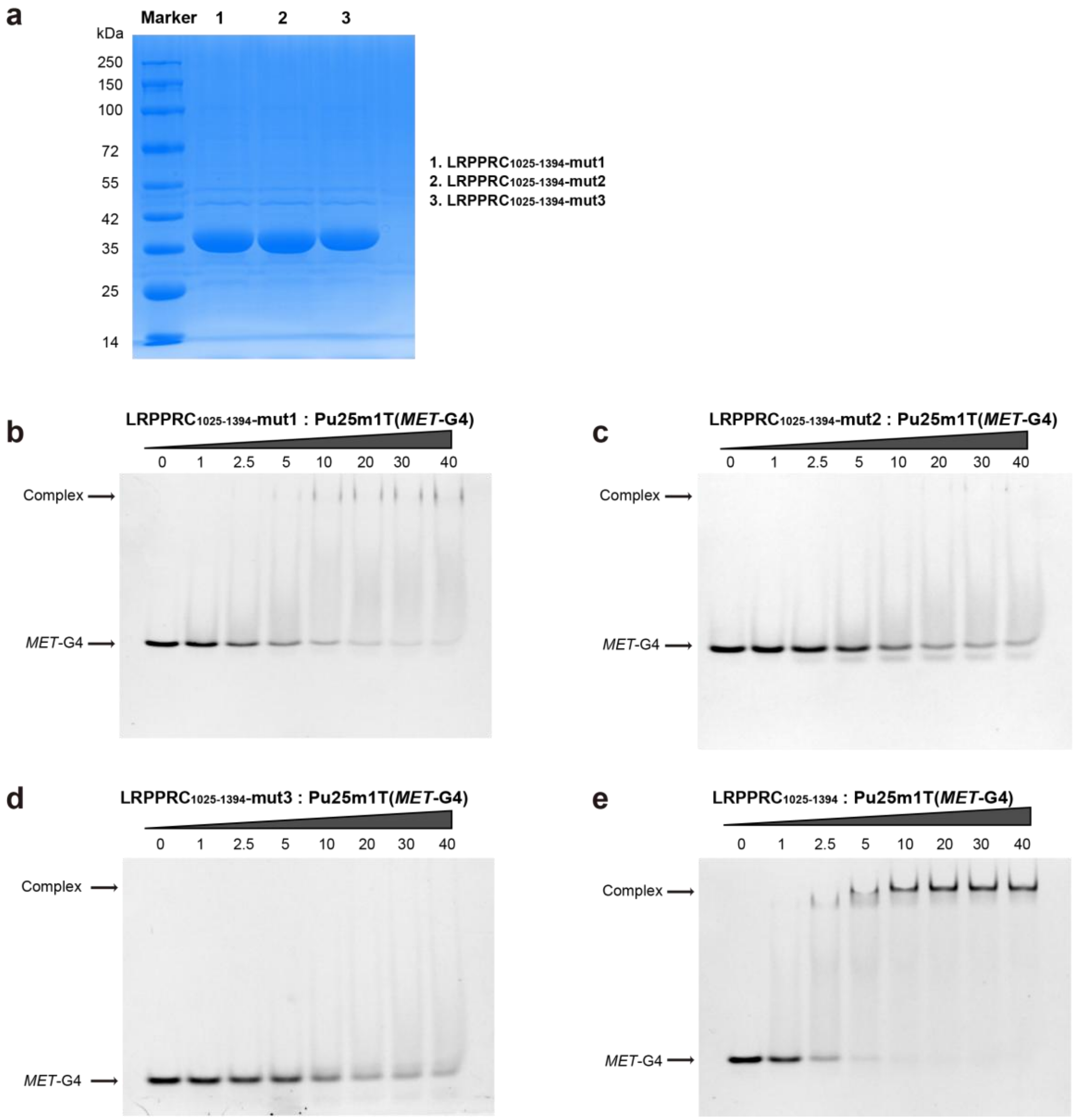

Supplementary Figure 12 | The binding ability analysis of the mutant LRPPRC$_{1025\text{-}1394}$ protein to *MET*-G4. a, Coomassie blue-stained SDS-PAGE analysis of three purified LRPPRC mutation fragments. **b**-**e**, Binding of LRPPRC$_{1025\text{-}1394}$-mut1, LRPPRC$_{1025\text{-}1394}$-mut2, LRPPRC$_{1025\text{-}1394}$-mut3 and LRPPRC$_{1025\text{-}1394}$ to Pu25m1T (*MET*-G4 DNA). Condition: 0.1 μM FAM-labeled DNA, and the corresponding ratios of protein to DNA are labeled. The experiment was repeated three times independently with similar results; representative images are shown. Source data are provided as a Source Data file.

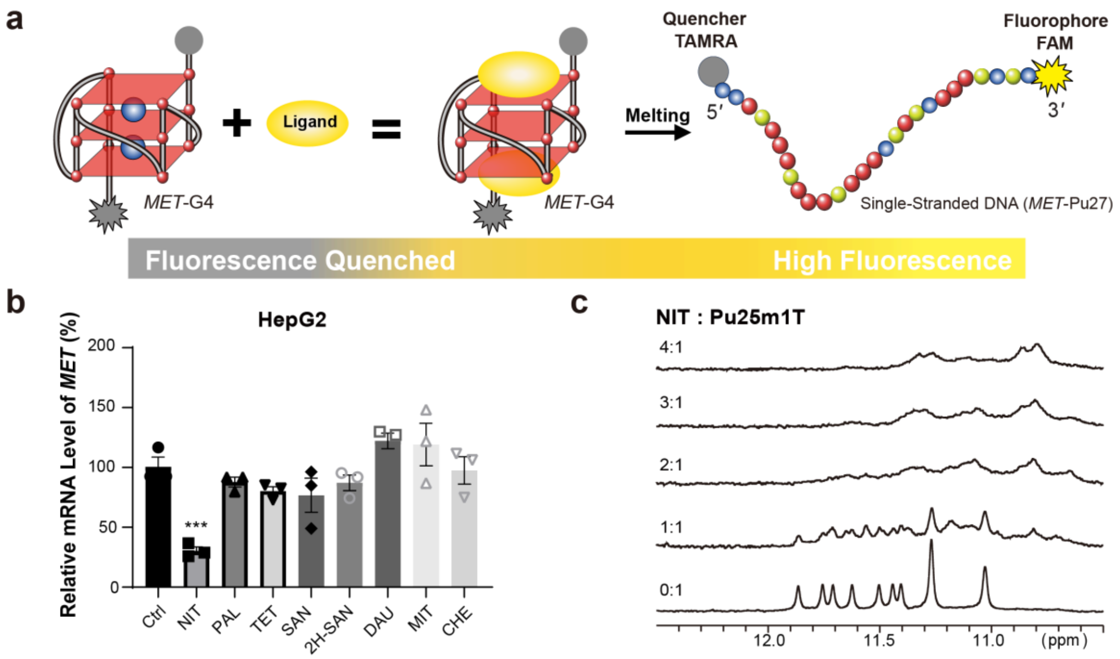

Supplementary Figure 13 | Screening of nitidine as a strong *MET*-G4 stabilizer. a, Schematic illustration of the FRET-melting assay utilized for compound screening. The FRET quenching (indicated by the black fluorophore) is induced by *MET*-G4 folding upon binding to *MET*-G4-stabilizing ligands. Fluorescence melting curves were recorded during oligonucleotide melting. **b**, RT-qPCR analysis of *MET* mRNA expression in HepG2 cells treated with eight hit compounds (1 μM) identified from FRET-melting assay (NIT CAS-number: 13063-04-2, PAL CAS-number: 10605-02-4, TET CAS-number: 518-34-3, SAN CAS-number: 5578-73-4, 2H-SAN CAS-number: 3606-45-9, DAU CAS-number: 70553-76-3, MIT CAS-number: 65271-80-9, CHE CAS-number: 3895-92-9). The data represent mean ± S.E.M. of results from three independent experiments. ****p* = 0.0005, one-way ANOVA. **c**, ^{1}H NMR spectra displaying the imino region changes of *MET*-G4 upon titration with nitidine. Conditions: 25 °C, 150 μM DNA, pH 7.0, 50 mM K^{+}. Source data are provided as a Source Data file.

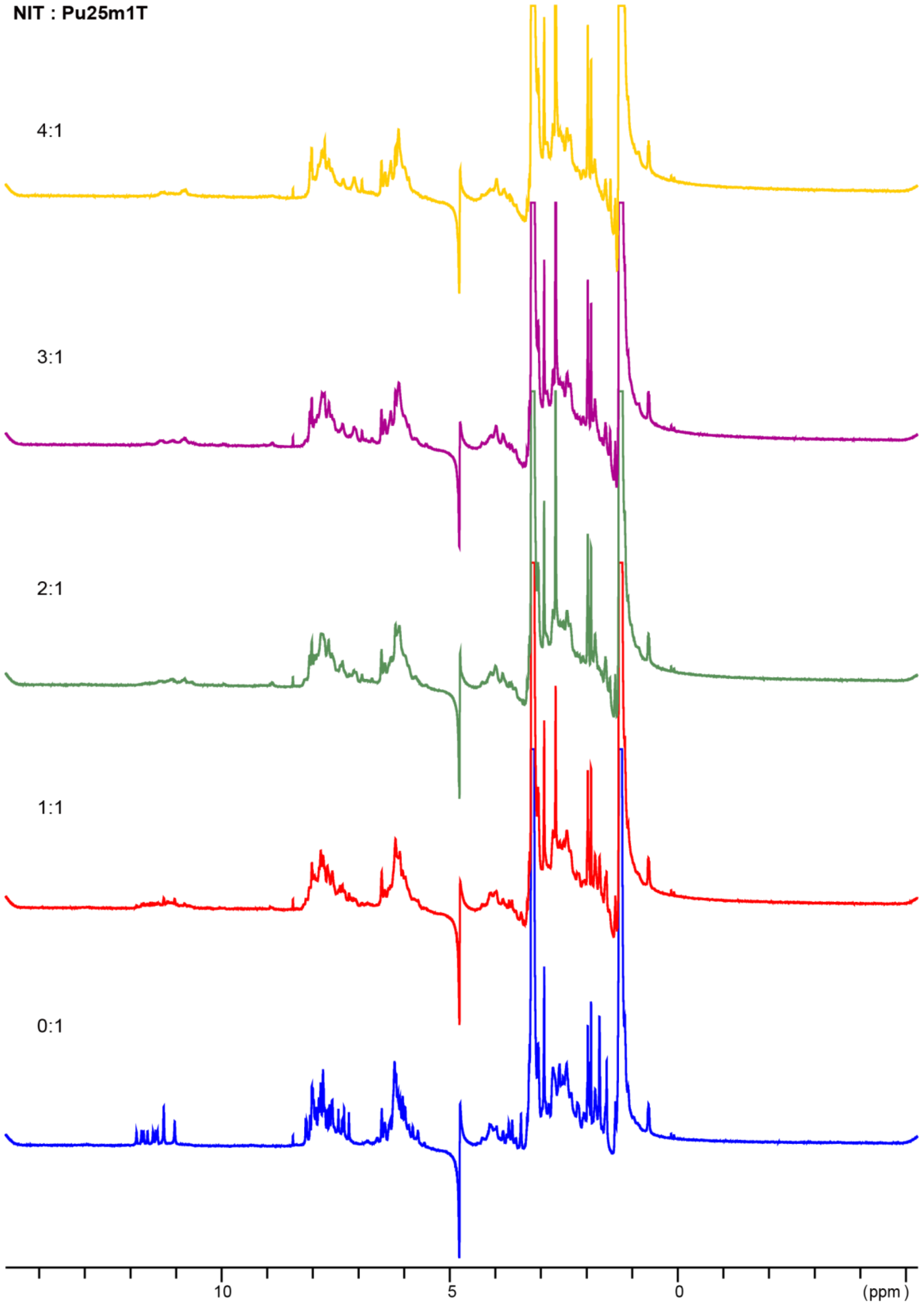

Supplementary Figure 14 | The full 1D ^{1}H NMR spectra of Pu25m1T DNA with and without nitidine, respectively. Conditions: 150 μM DNA, pH 7.0, 50 mM K^{+}, 25 °C, DMSO-d_6 < 3.5%.

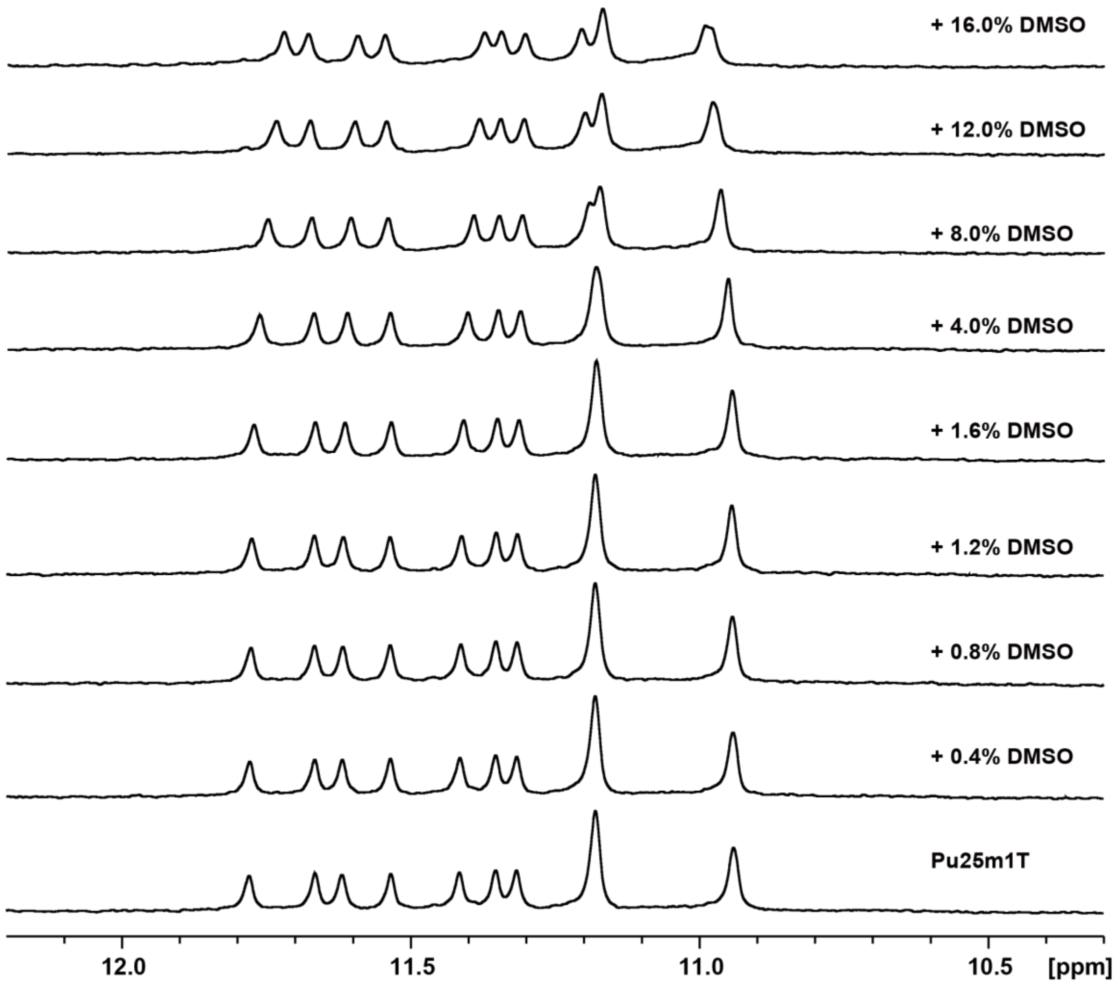

Supplementary Figure 15 | 1D ^{1}H NMR titration of *MET*-G4 DNA with increasing concentration of DMSO-*d_6*. Volume percentages of DMSO-d_6 are shown on the right side of the spectra. Conditions: 150 μM DNA, pH 7.0, 50 mM K^+, 25 °C. Source data are provided as a Source Data file.

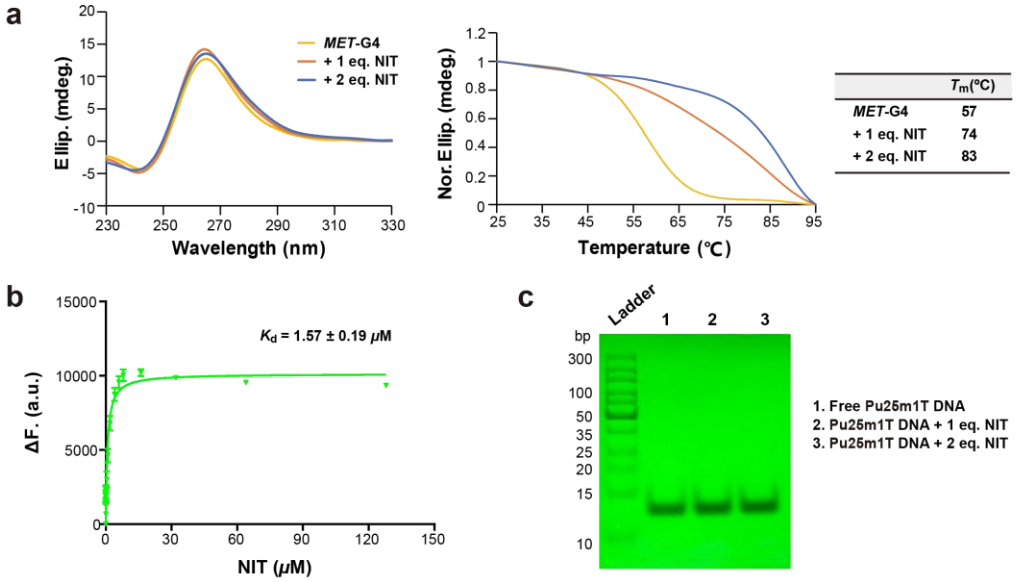

Supplementary Figure 16 | Nitidine is a strong *MET*-G4 stabilizer. a, ECD spectra and ECD thermal melting curves of *MET*-G4 and *MET*-G4 in the presence of varying concentrations of nitidine. Conditions: 20 μM DNA, pH 7.0, 10 mM K^+. **b**, Fluorescence intensity change of FAM-labeled Pu25m1T DNA (*MET*-G4) upon titration with nitidine. K_d value was determined. Conditions: 50 nM DNA, pH 7.0, 50 mM K^+ solution. The process was conducted in triplicate. **c**, Native EMSA gel of Pu25m1T, Pu25m1T + 1. eq nitidine, and Pu25m1T + 2. eq nitidine. Conditions: 100 μM DNA, pH 7.0, 2 mM K^+. Each sample contained 5 μL of 100 μM DNA. DNA bands were visualized under UV light at 254 nm. Source data are provided as a Source Data file.

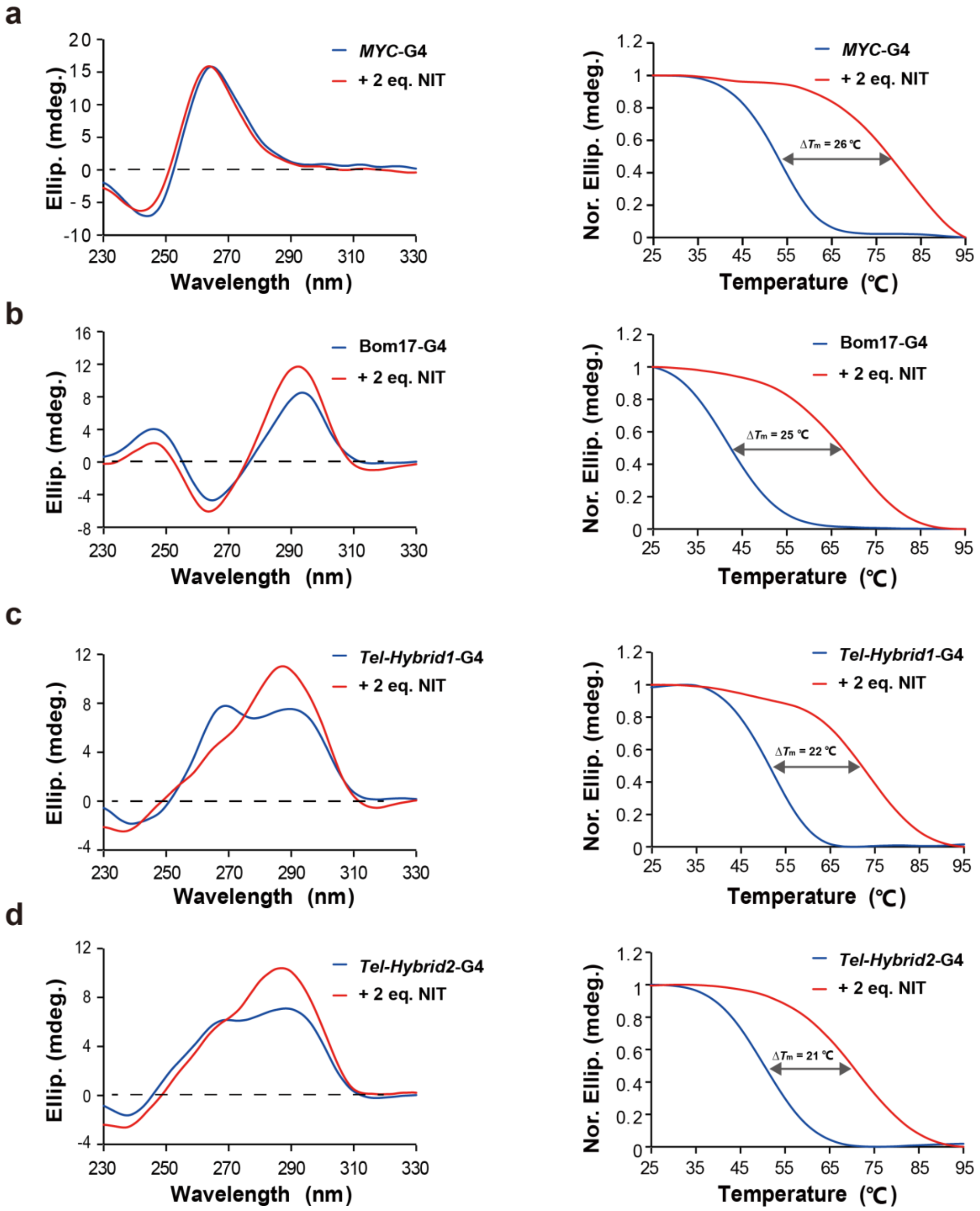

Supplementary Figure 17 | ECD spectra and ECD thermal melting curves of *MYC*-G4, Bom17-G4, *Tel-hybrid1*-G4, and *Tel-hybrid2*-G4 in the presence of 2 eq. nitidine, respectively. Conditions: 20 μM DNA, pH 7.0, 40 μM compound, 1-50 mM K^+. The changes in melting temperature (ΔT_m) were labeled. Source data are provided as a Source Data file.

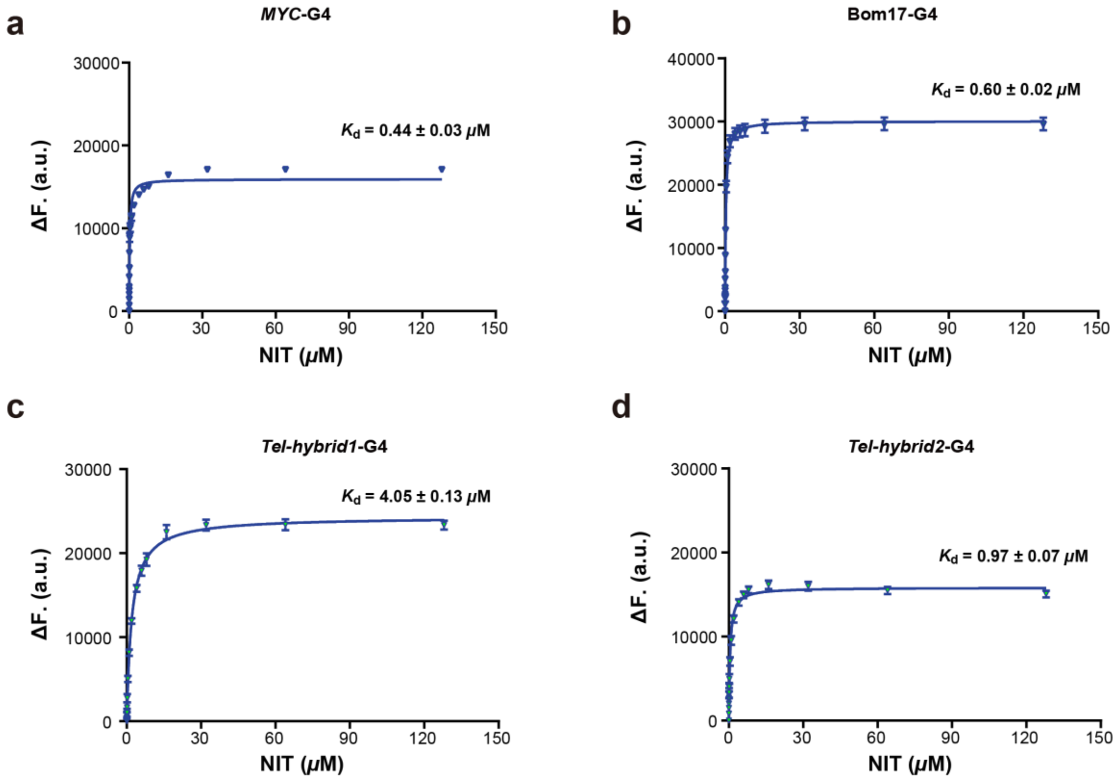

Supplementary Figure 18 | Fluorescence intensity changes of FAM-labeled *MYC*-G4, Bom17-G4, *Tel-hybrid1*-G4, and *Tel-hybrid2*-G4 upon titration with nitidine, respectively. K_d value was labeled. Conditions: 50 nM DNA, pH 7.0, 50 mM K^+ solution. The process was conducted in triplicate. Source data are provided as a Source Data file.

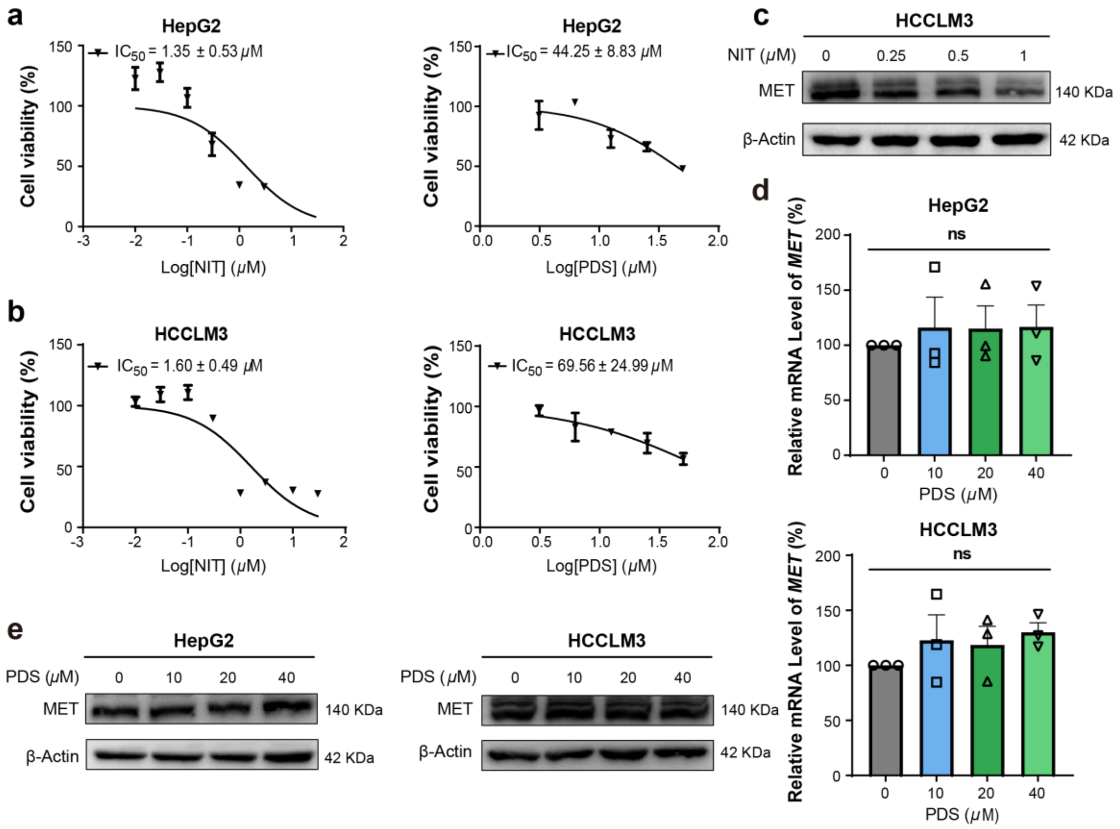

Supplementary Figure 19 | Cell viability and MET protein/mRNA expression levels after nitidine/pyridostatin treatment in HepG2/HCCLM3 cells. a and **b**, HepG2 and HCCLM3 cells were treated with various concentrations of nitidine or pyridostatin for 48 h, and the cell viability was analyzed by CCK-8 assay. **c**, Western blotting analysis of MET protein expression levels in HCCLM3 cells treated with nitidine for 48h. The experiment was repeated three times independently with similar results; representative images are shown. **d**, RT-qPCR analysis of *MET* mRNA expression levels in HepG2 and HCCLM3 cells treated with pyridostatin. Data are presented as mean ± S.E.M. from three independent experiments. ns: not significant, one-way ANOVA. **e**, Western blotting analysis of MET protein expression levels in HepG2 and HCCLM3 cells treated with pyridostatin. The experiment was repeated three times independently with similar results; representative images are shown. Source data are provided as a Source Data file.

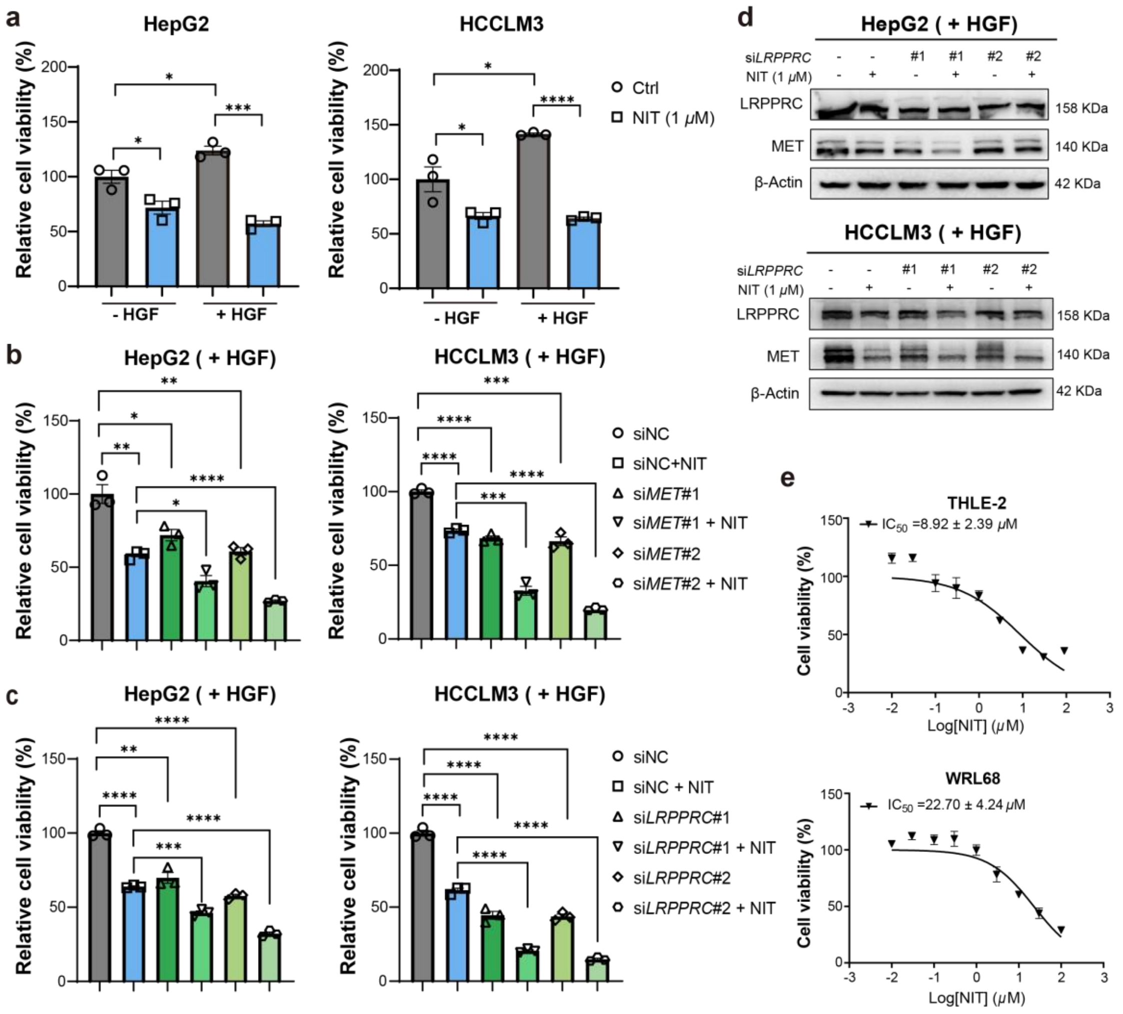

Supplementary Figure 20 | Cell viability after nitidine/si*LRPPRC*/si*MET* treatment in HepG2/HCCLM3 cells. a, Cell viability was assessed using the CCK-8 assay in 40 ng/mL HGF-stimulated HepG2 and HCCLM3 cells treated with 1 μM nitidine for 24h. Data are presented as mean ± SEM; $n = 3$, *$p = 0.028$, *$p = 0.029$, ***$p = 0.0001$ (for HepG2), *$p = 0.047$, *$p = 0.022$, ****$p = 0.0000006$ (for HCCLM3), two-tailed t-test. **b**, Cell viability was assessed using CCK-8 assay in 40 ng/mL HGF-stimulated HepG2 and HCCLM3 cells treated with si*MET* and/or 1 μM nitidine for 24h. Data are presented as mean ± S.E.M. from three independent experiments.; $n = 3$, *$p = 0.02$, *$p = 0.0129$, **$p = 0.0032$, **$p = 0.0047$, ****$p = 0.0000647$, (for HepG2); ***$p = 0.0002$, ***$p = 0.0005$, ****$p = 0.00006$, ****$p = 0.00009$, (for HCCLM3), two-tailed t-test. **c**, Cell viability was assessed using the CCK-8 assay in 40 ng/mL HGF-stimulated HepG2 and HCCLM3 cells treated with si*LRPPRC* and/or nitidine. **$p = 0.0016$, ***$p = 0.0003$, ****$p < 0.0001$, statistical analysis was performed using two-tailed t-tests. **d**, Western blotting analysis for assessing the protein expression levels of MET and LRPPRC in 40 ng/mL HGF-stimulated HepG2 and HCCLM3 cells treated with si*LRPPRC* and/or nitidine. The experiment was repeated three times independently with similar results; representative images are shown. **e**, THLE-2 and WRL68 cells were treated with various concentrations of nitidine for 48 h, the cell viability was analyzed by CCK-8 assay. Source data are provided as a Source Data file.

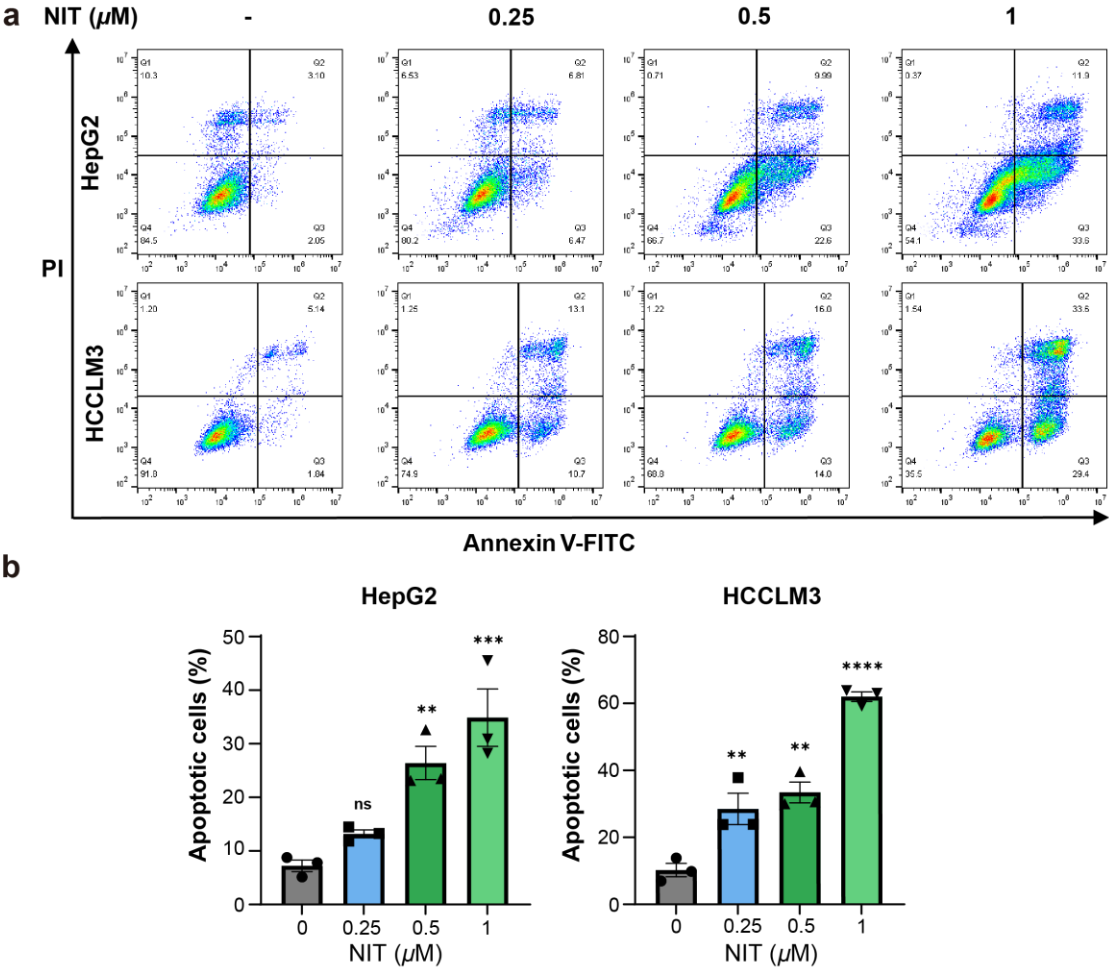

Supplementary Figure 21 | Nitidine induces apoptosis in HepG2 and HCCLM3 cells. a and **b**, The cell apoptosis was analyzed by flow cytometry in HepG2 and HCCLM3 cells treated with different concentrations of nitidine. Data are presented as mean ± S.E.M; ns: not significant, ** $p = 0.0069$, *** $p = 0.0007$ (for HepG2), ** $p = 0.0073$, ** $p = 0.0017$, ****$p < 0.0001$ (for HCCLM3), one-way ANOVA. Source data are provided as a Source Data file.

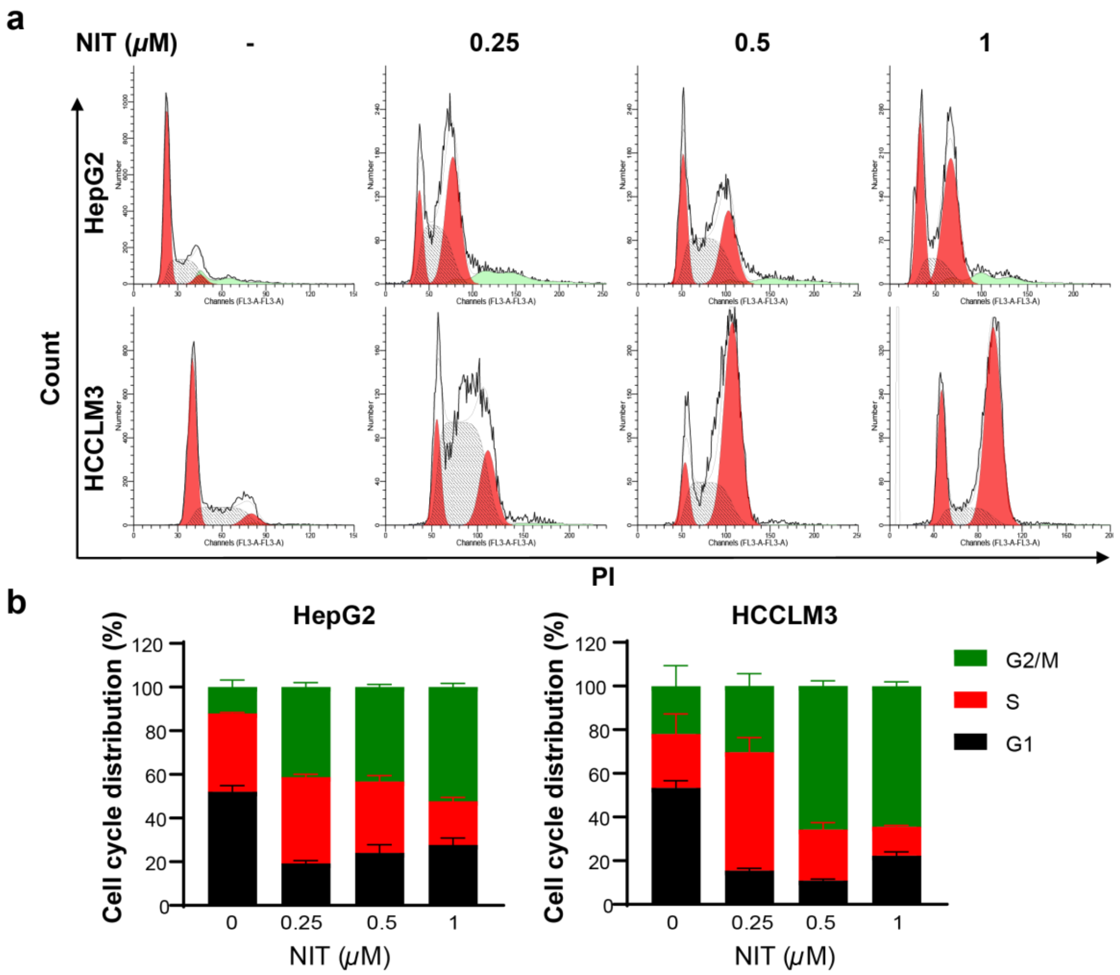

Supplementary Figure 22 | Nitidine induces cell cycle arrest in HepG2 and HCCLM3 cells. a and **b**, Cell cycle distribution in HepG2 and HCCLM3 cells treated with varying concentrations of nitidine for 24 h. Source data are provided as a Source Data file.

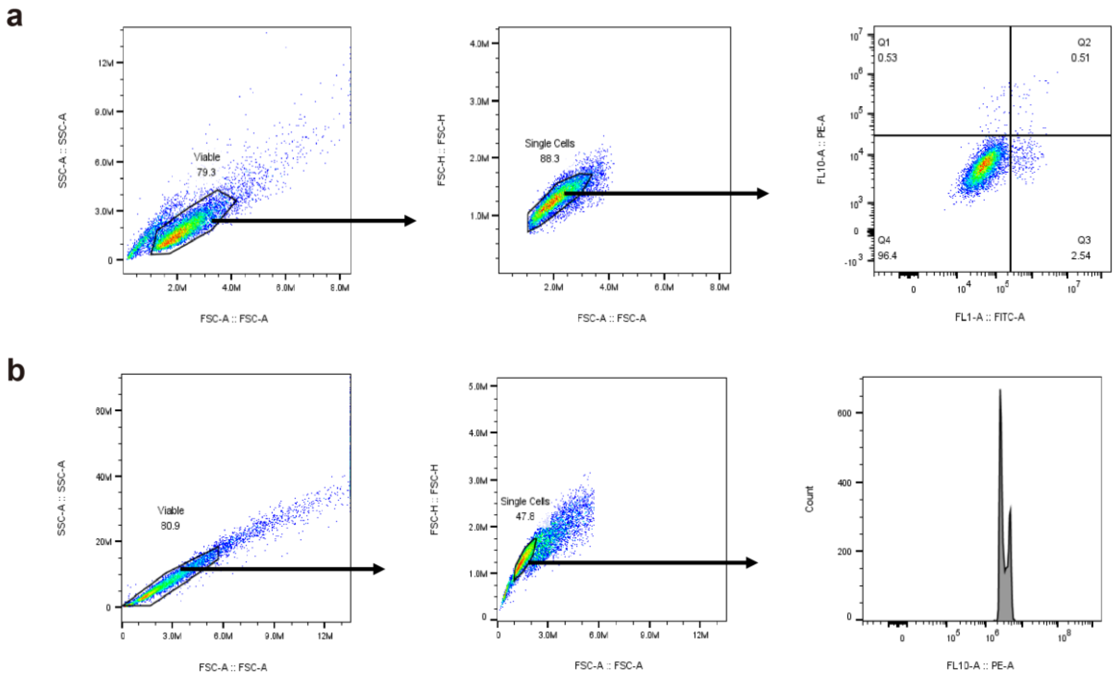

Supplementary Figure 23 | Graphical accounts for all FACS sequential gating strategies. a, An example gating scheme for flow cytometry experiments is shown. Initial FSC-Area/SSC-Area gates are drawn to remove debris and dead cells. A subsequent FSC-Area/FSC-Height gate demarcates singlet events. After gating on the single cell population, the cells were classified as live cells (FITC$^-$PI$^-$), early apoptotic cells (FITC$^+$PI$^-$), late apoptotic cells (FITC$^+$PI$^+$), and necrotic cells (FITC$^-$PI$^+$). **b**, An example gating scheme for flow cytometry experiments is shown. Initial FSC-Area/SSC-Area gates are drawn to remove debris and dead cells. A subsequent FSC-Area/FSC-Height gate demarcates singlet events. After gating on the single cell population, the cells were classified into G1, S, and G2/M phases.

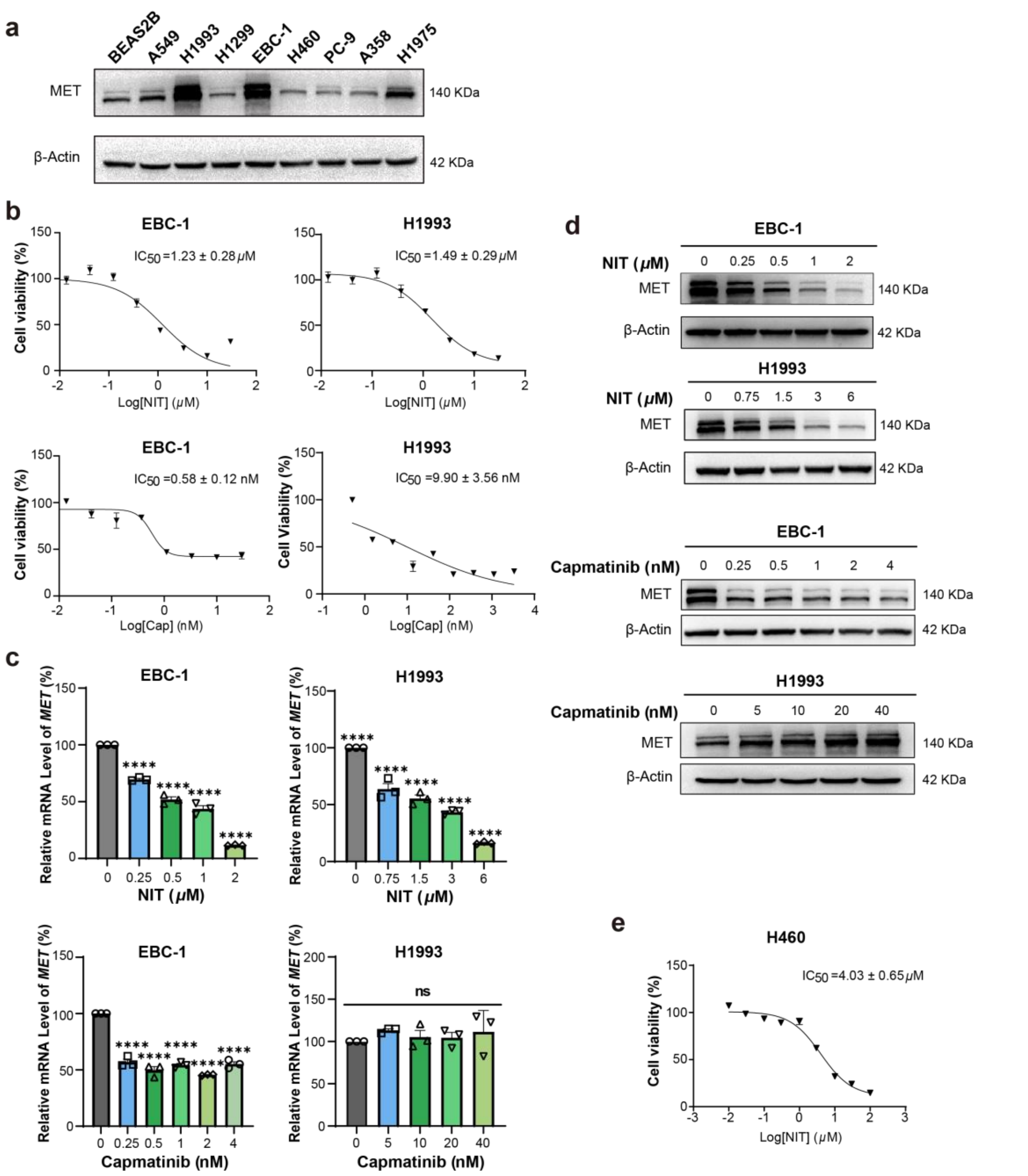

Supplementary Figure 24 | MET expression levels in NSCLC cells following nitidine or capmatinib treatment. a, Western blotting analysis of MET protein levels in BEAS2B, A549, H1993, H1299, EBC-1, H460, PC-9, A358, and H1975 cells. Three independent experiments were performed with similar results; representative images are shown. **b**, EBC-1 and H1993 cells were treated with various concentrations of nitidine or capmatinib for 48 h, and the cell viability was analyzed by CCK-8 assay. **c**, RT-qPCR analysis of *MET* mRNA levels in EBC-1 and H1993 cells treated with nitidine or capmatinib. Data are presented as mean ± S.E.M. from three independent experiments. ****$p < 0.0001$, ns: not significant, one-way ANOVA. **d**, Western blotting analysis of MET protein levels in EBC-1 and H1993 cells treated with nitidine or capmatinib for 48 h. Three independent experiments were performed with similar results; representative images are shown. **e**, H460 cells were treated with various concentrations of nitidine for 48 h, the cell viability was analyzed by CCK-8 assay. Source data are provided as a Source Data file.

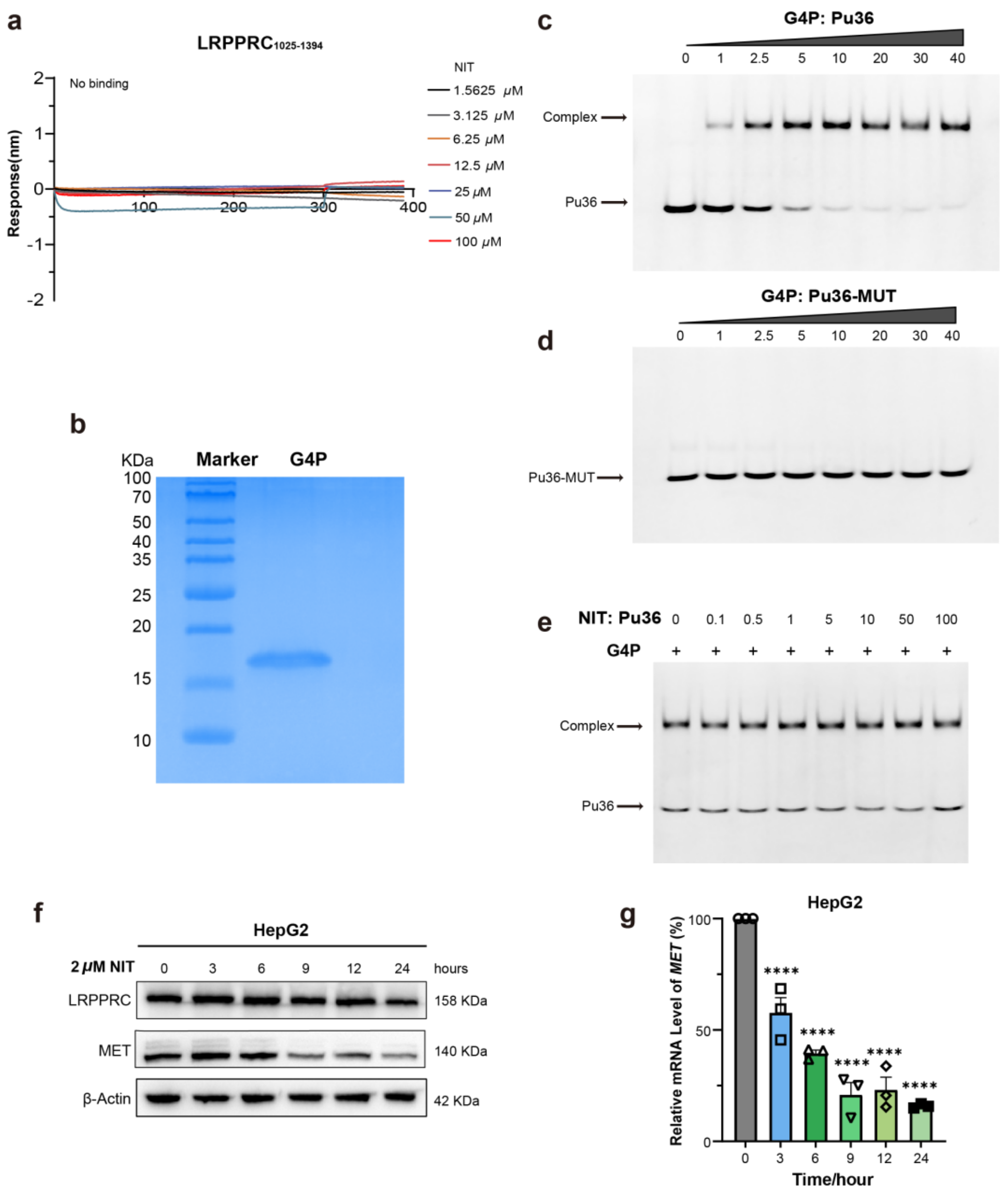

Supplementary Figure 25 | Binding activity analysis of nitidine to purified LRPPRC$_{1025\text{-}1394}$ with BLI, binding activity analysis of nitidine to Pu36-G4P complex, and time-dependent MET/LRPPRC expression levels with nitidine treatment in HepG2 cells. a, BLI analysis showing no significant binding activity of nitidine to the LRPPRC$_{1025\text{-}1394}$ protein. **b**, Coomassie blue-stained SDS-PAGE of purified G4P. **c** and **d**, EMSA gel result of G4P binds to Pu36 (36-nt *MET*-G4 forming sequence) or Pu36-MUT DNA. Conditions: 0.1 μM FAM-labeled DNA, and the corresponding ratio of protein to DNA was labeled. **e**, EMSA gel result of nitidine to Pu36-G4P complex. The ratio of G4P to Pu36 DNA was 2.5, and the ratio of nitidine to Pu36 DNA was labeled. **f**, Western blotting for assessing the protein expression levels of MET and LRPPRC in HepG2 cells treated with 2 μM nitidine at various time points. Three independent experiments were performed with similar results; representative images are shown. **g**, Relative *MET* mRNA expression levels in HepG2 cells treated with 2 μM nitidine at various time points. The data represent mean ± S.E.M. of results from three independent experiments. ****$p < 0.0001$, one-way ANOVA. Source data are provided as a Source Data file.

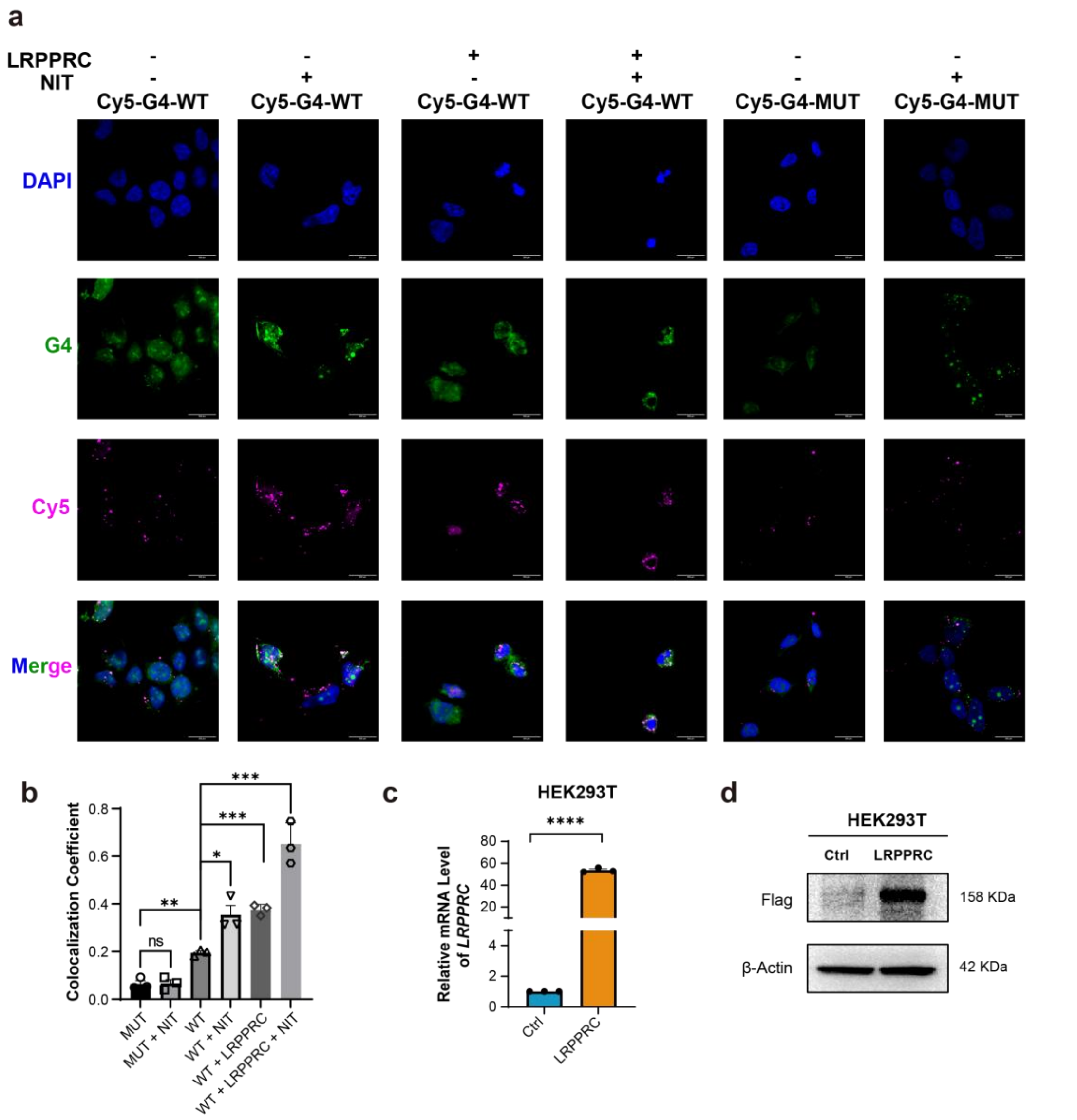

Supplementary Figure 26 | Quantitative analysis of colocalization between Cy5-oligo and FAM-G4P. a Representative immunofluorescence image showing FAM-G4P (green), Cy5-oligo (red), and their merged view in HEK293T cells. Scale bar: 200 µm. Three independent experiments were performed with similar results; representative images are shown. **b**, Quantification of the colocalization using Manders' overlap coefficient (M1), which represents the fraction of Cy5-oligo signal overlapping with FAM-G4P. Data are presented as mean ± SEM from three independent images. Two-tailed *t*-tests: ns: not significant, *p = 0.0171, **p = 0.0012, ***p = 0.003, ***p = 0.009. **c** and **d**, LRPPRC mRNA and protein levels in HEK293T cells after LRPPRC overexpression. The western bloting was repeated three times independently with similar results; representative images are shown. RT-qPCR data, means ± S.E.M., from three independent experiments. Statistical analysis was performed using a two-tailed *t*-test. ****p = 0.000001. Source data are provided as a Source Data file.

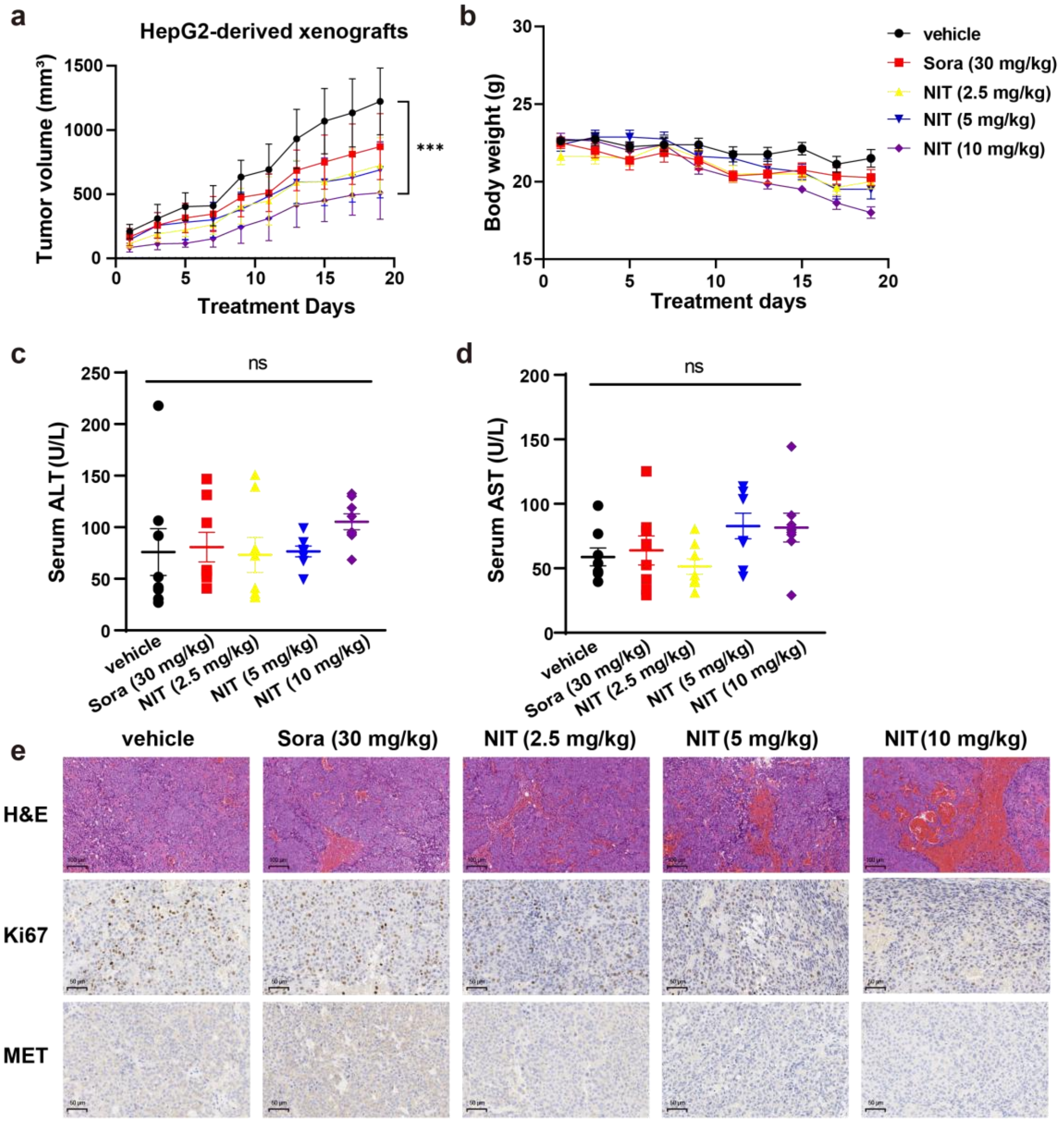

Supplementary Figure 27 | Nitidine inhibits HCC progression *in vivo*. a, The tumor growth curves in mice during treatment. ***p = 0.0001, two-way ANOVA. **b**, The body weights of mice were monitored during treatment. **c** and **d**, Serum alanine aminotransferase (ALT) and aspartate aminotransferase (AST) levels were measured. ns, not significant, one-way ANOVA. **e**, Representative images of hematoxylin and eosin (H&E) (Scale bar, 100 μm), Ki67 staining (Scale bar, 50 μm), and IHC staining of MET (Scale bar, 50 μm) in tumor tissues are shown. Source data are provided as a Source Data file.

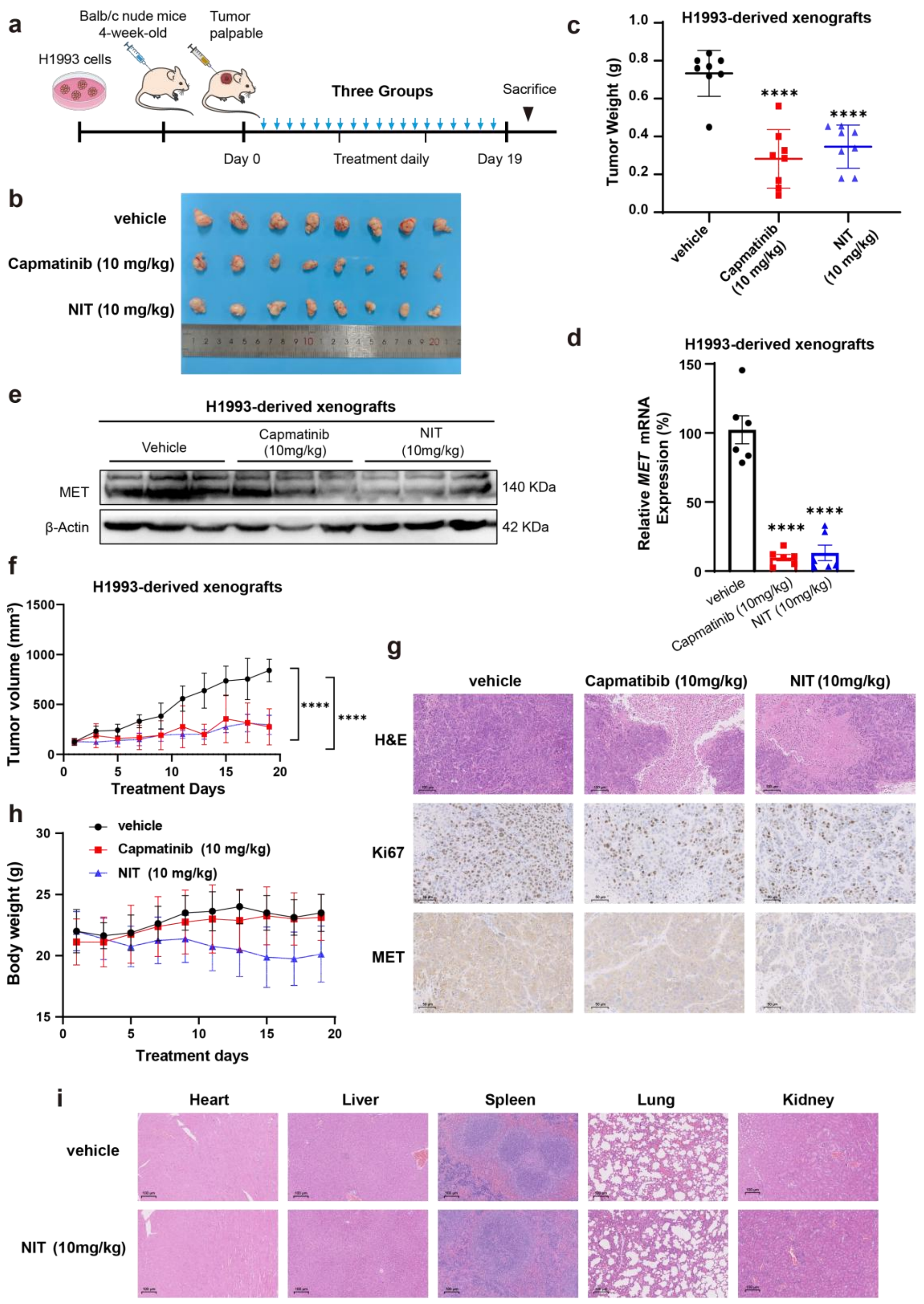

Supplementary Figure 28 | Nitidine inhibits NSCLC progression in vivo. a, Schematic diagram of H1993-derived xenograft mouse model with intraperitoneally administered with nitidine (10 mg/kg/day), $n = 8$, or orally administered with capmatinib (10 mg/kg/day), $n = 8$. Created in BioRender. Li, Y. (2026) https://BioRender.com/8q4p3q6. **b** and **c**, b) Representative image of excised tumors and c) corresponding tumor weights were shown. ****$p = 0.000014$, ****$p = 0.000012$, two-tailed t-test. **d** and **e**, Analysis of protein and mRNA levels of MET in H1993-derived xenografts following nitidine or

capmatinib treatment. ****$p = 0.000005$, ****$p = 0.000017$, two-tailed t-test. **f**, The tumor growth curves in mice during treatment. ****$p < 0.0001$, two-way ANOVA. **g**, Representative images of hematoxylin and eosin (H & E) (Scale bar, 100 μm), Ki67 staining (Scale bar, 50 μm), and IHC staining of MET (Scale bar, 50 μm) in tumor tissues were shown. **h**, The body weights of mice were monitored during treatment. **i**, Representative H&E images in the heart, liver, spleen, lung, and kidney from mice treated with vehicle, nitidine (10mg/kg) for 19 days. Scale bar, 100 μm. Source data are provided as a Source Data file.

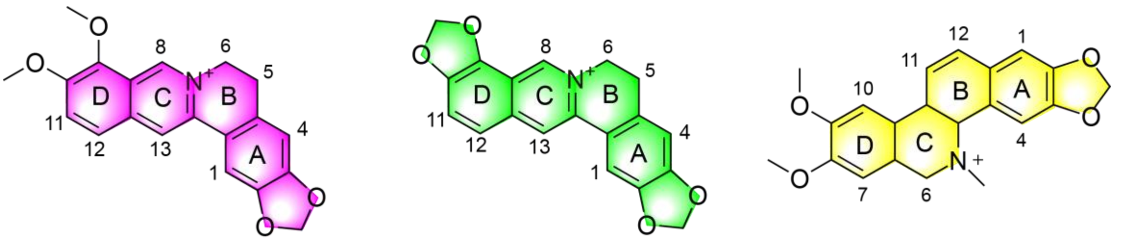

Berberine(Ber) Coptisine(Cop) Nitidine(Nit)

Supplementary Figure 29 | Chemical structure of berberine, coptisine, and nitidine with numbering.

Supplementary Table 1. The nucleotide sequences were used in this study.

Sequence Name	DNA Sequence (5′ to 3′)	Assay
Pu24	GCGGGCGGGCGGGGCGCTGGGCTC	NMR, CD
Pu25T	TGCGGGCGGGCGGGGCGCTGGGCTC	NMR, CD, EMSA
Pu25m1T (*MET*-G4)	TGCGGGCGGGCGGGTCGCTGGGCTC	NMR, CD
Pu36-wt_3′-FAM	TGCGGCCGGCGGGCGGGCGGGGCGCTGGGCTC AGCC-FAM-3′	DMS footprinting
5′-end FAM labeled primer	5′-FAM-TAATACGACTCACTATAGCAATTGC	DNA polymerase stop assay
MET-G4 containing Template	TAGTGCTGCCTGCGGGCGGGCGGGGCGCTGGG CTCAAGATAGCTGCACGCAATTGCTATAGTGA GTCGTATTA	
Pu27_TAMRA_FAM	5′-TAMRA- TTGCGGGCGGGCGGGTCGCTGGGCTCT-FAM-3′	FRET
3′-TEG-Biotin labeled *MET*-G4	TTTTTTTTTTTTTTTGCGGGCGGGCGGGGCGCT GGGCTC-TEG-Biotin-3′	Pull down
3′-TEG-Biotin labeled MUT-G4	TTTTTTTTTTTTTTTGCGTGCGTGCGTGGCGCTG TGCTC-TEG-Biotin-3′	
Pu36-wt_5′-FAM	5′-FAM- TGCGGCCGGCGGGCGGGCGGGGCGCTGGGCTC AGCC	EMSA
Pu36-mut_5′-FAM	5′-FAM- TGCGGCCGGCGTGCGTGCGTGGCGCTGTGCTC AGCC	
Pu25m1T (*MET*-G4)_5′-FAM	5′-FAM-TGCGGGCGGGCGGGTCGCTGGGCTC	EMSA, MST
MUT-G4_5′-FAM	5′-FAM-TGCGTGCGTGCGTGTCGCTGTGCTC	
MYC-G4	TGAGGGTGGGTAGGGTGGGTAA	CD, EMSA
Bom17-G4	GGTTAGGTTAGGTTAGG	
Tel-hybrid1-G4	AAAGGGTTAGGGTTAGGGTTAGGGAA	
Tel-hybrid2-G4	TTAGGGTTAGGGTTAGGGTTAGGGTT	
MYC-G4_5′-FAM	5′-FAM-TGAGGGTGGGTAGGGTGGGTAA	Fluorescence Measurement
Bom17-G4_5′-FAM	5′-FAM-GGTTAGGTTAGGTTAGG	
Tel-hybrid1-G4_5′-FAM	5′-FAM-AAAGGGTTAGGGTTAGGGTTAGGGAA	
Tel-hybrid2-G4_5′-FAM	5′-FAM-TTAGGGTTAGGGTTAGGGTTAGGGTT	
Cy5-G4-WT (*MET*-G4)	5′-Cy5-TGCGGGCGGGCGGGTCGCTGGGCTC	Immunofluorescence
Cy5-G4-MUT	5′-Cy5-TGCGTGCGTGCGTGTCGCTGTGCTC	
Primers used for RT-qPCR		

Genes	Forward sequence (5′ to 3′)	Reverse sequence (5′ to 3′)
Human		
GAPDH	GACCTGCCGTCTAGAAAAAC	TTGAAGTCAGAGGAGACCAC
MET	AGCAATGGGGAGTGTAAAGAGG	CCCAGTCTTGTACTCAGCAAC
LRPPRC	GCTCATAGGATATGGGACACACT	CCAGGAAATCAGTTGGTGAGAAT
EIF3A	GCAAAGGAGGGGTTATACCAGT	TGTCAGTACGATCCTGAGTGTC
HADHA	ATATGCCGCAATTTTACAGGGT	ACCTGCAATAAAGCAGCCTGG
SFPQ	AGCGATGTCGGTTGTTTGTTG	AGCGAACTCGAAGCTGTCTAC
HNRNPM	CTCTTAATGGACGCTGAAGGAAA	CGCTCAGACTATGCTTGTTTAGG
PABPC1	CAGGCTCACCTCACTAACCAG	GGTAGGGGTTGATTACAGGGT
MCM5	ATGTCGGGATTCGACGATCCT	CCAGGTTGTAATGCCGCTTG
QARS	CCTGTCGCTCTTCACTAGCC	GGGAGGCCAAGCCATATAACA
DHX9	CAGGAGAGAGAGTTACTGCCT	CTCTGCTGCTCGGTCATTCTG
PABPC3	ATCCTTTCGTGTAACGTGGTTT	ACATTGGGGAACTCTTTTGCC

Primers used for ChIP-qPCR		
Genes	Forward sequence (5′ to 3′)	Reverse sequence (5′ to 3′)
MET-site (-200 to +100 bp)	AGTTTCACCTTGTCGTGGGC	CACGTGTCTGTCTGCCTCG

siRNA sequences for knockdown	
Genes	siRNA sequence (5′ to 3′)
siNC	ACGUGACACGUUCGGAGAA
si*MET*#1	CAATCATACTGCTGACATA
si*MET*#2	GTCGGAGGTTCACTGCATA
si*LRPPRC*#1	GGACCGTGATTTACTGCAA
si*LRPPRC*#2	CATCGTGACTCTAGGGTTA
si*EIF3A*	CAGTTGATGGCAAATTACT
si*HADHA*	TGGTGACAAGATTTGTGAA
si*SFPQ*	CUUUCUGUUCGUAAUCUUUCA
si*HNRNPM*	GACUUGGAAGCACAGUAUU
si*PABPC1*	UCACUGGCAUGUUGUUGGA
si*MCM5*	GGGTTACCATCATGGGCAT
si*QARS*	GGTGGAACGTTACCATTTC
si*DHX9*	GAGCCAACUUGAAGGAUUA
si*PABPC3*	AGGCTTACCTCACTAACGA

Supplementary Table 2. Proton chemical shifts of *MET*-G4 (Pu25m1T) at 15 °C in pH 7.0, 25 mM K^+-containing solution.

Base	H1/H2/H5	HMe	H6/H8	H1′	H2′, H2″	H3′	H4′	H5′, H5″*
T1		1.84	7.46	5.91	1.86, 2.20	4.56	3.80	3.55, 3.55
G2			7.52	6.00	2.70, 2.70	4.79	4.32	3.87, 3.99
C3	5.97		7.75	6.16	2.21, 2.55	4.87	3.82	3.72, 4.00
G4	11.98		8.31	6.34	2.98, 3.28	5.11	4.62	4.11, 4.24
G5	11.54		7.97	6.33	2.87, 3.09	5.18	4.71	4.47, 4.47
G6	11.40		7.91	6.54	2.71, 2.82	5.22	4.73	4.23, 4.53
C7	6.30		8.14	6.62	2.54, 2.87	5.22	4.76	4.42, 4.51
G8	11.89		8.13	6.25	2.56, 3.09	5.30	4.60	4.40, 4.50
G9	11.40		8.07	6.28	2.76, 3.00	5.21	4.70	4.40, 4.40
G10	11.15		7.89	6.56	2.74, 2.86	5.23	4.77	4.41, 4.51
C11	6.30		8.14	6.62	2.54, 2.87	5.22	4.76	4.42, 4.51
G12	11.75		8.16	6.27	2.58, 3.05	5.31	4.61	4.38, 4.38
G13	11.56		8.13	6.20	2.85, 2.95	5.21	4.67	4.35, 4.42
G14	11.15		7.91	6.47	2.72, 2.72	5.11	4.59	4.34, 4.34
T15		2.07	7.76	6.33	2.44, 2.57	4.94	4.34	4.1, 4.12
C16	6.21		7.87	6.33	2.20, 2.57	4.87	4.38	4.10, 4.10
G17			8.20	6.37	2.96, 2.83	5.14	4.53	4.27, 4.27
C18	6.11		7.97	6.44	2.48, 2.75	5.00	4.54	4.35, 4.25
T19		1.83	7.71	6.16	2.43, 2.63	5.07	4.41	4.23, 4.23
G20	11.86		8.30	6.22	2.89, 3.03	5.12	4.58	4.24, 4.24
G21	11.63		8.00	6.25	2.80, 2.87	5.16	4.70	4.42, 4.42
G22	11.40		7.80	6.36	2.71, 2.72	5.13	4.66	4.42, 4.42
C23	6.17		8.04	6.34	2.31, 2.66	4.78	4.43	4.21, 4.34
T24		1.66	7.31	5.80	1.95, 2.11	4.54	3.71	3.83, 3.93
C25	5.80		7.46	5.62	1.91, 2.30	4.28	3.74	3.53, 3.53

Note: *Assignments are not stereospecific.

Supplementary Table 3. Inter-residue NOEs of the Pu25m1T 5′-end capping structure.

	G2			
T1	H8	H4′	H5′	H5″
H6	W			
H1′	W	W	W	W
H2′	M			
H2″	M			
H3′	W			

	C3					G4	G8		G12	G20
G2	H5	H6	H1′	H5′	H5″	H1	H1	H8	H1	H1
H8	M	M	W	W	W	W			VW	VW
H1′				VW	VW	W	W	W		
H2′	W	W				M		W		VW
H2″	W	W				M		W		
H3′	W	M								
H4′	W	W								
H5′	W	W								
H5″	W	W								

	G4			G8	G12	G20
C3	H1	H8	H1′	H1	H1	H1
H5	W			W	VW	M
H6		W				W
H1′		M				M
H2′		W				VW
H2″		W				VW
H3′		W				
H4′		M	M			
H5′		W	M			
H5″		W	M			

Note: M = medium intensity, red marked, W = weak intensity, VW = very weak intensity.

Supplementary Table 4. Inter-residue NOEs of the Pu25m1T 3′-end capping structure.

	G22						T24			
C23	H8	H1′	H2′	H2″	H3′	H4′	H6	Me	H5′	H5″
H5			VW	VW	W	W				
H6	W	W	M	M	W	W	VW	W		
H1′							W		W	W
H2′	W	M					M	W		
H2″	W	M					M	W		
H3′	W						M			
H4′							W			
H5′	W						W			
H5″	VW						W			

	G6	G14		G22			C25
T24	H1	H1	H1'	H1	H1'	H8	H6
H6		M		M		W	
Me		VW	VW		W	M	
H1′	W	W		M			M
H2′		VW		VW			W
H2″		VW		W			M
H3′							W
H4′		VW		M			W
H5′				W			
H5″				W			

	G6	G10	
C25	H1	H1	H8
H5	W	OL	
H6	M	W	
H1′	W	VW	W
H2′	VW		
H2″	VW		
H3′	OL		
H4′	W		
H5′	W		
H5″	W		

Note: M = medium intensity, red marked, W = weak intensity, VW = very weak intensity, OL = overlapped cross-peak.

Supplementary Table 5. Inter-residue NOEs of the Pu25m1T loop ($T_{15}C_{16}G_{17}C_{18}T_{19}$) structure.

	G13		G14								
T15	H1′	H4′	H1	H8	H1'	H2'	H2"	H3'	H4'	H5'	H5"
H6					M	W	W	W	M	W	W
Me	W	W	VW	W	M	W	W		W	W	W
H1′											
H2′											
H2″											
H3′											
H4′											
H5′					VW						
H5″					VW						

	C16				G17		G20		G21	T24
T15	H5	H6	H5′	H5"	H1′	H4′	H8	H3′	H8	Me
H6			VW	VW	W					
Me							VW	W	VW	VW
H1′		W	OL	OL		M				
H2′	W	W			W					
H2″	W	W			W					
H3′	W	W								
H4′										
H5′										
H5″										

	G17	
C16	H1′	H8
H6		W
H1′		M
H2′		W
H2″		M
H3′		W
H4′		M
H5′	W	W
H5″	W	W

Supplementary Table 5. (continued)

	C18				G20	
G17	H5	H6	H5′	H5″	H5′	H5″
H8	W	W			W	W
H1′			OL	OL	W	W
H2′	W	W				
H2″	W	W				
H3′	W	OL				
H4′	W					
H5′	W					
H5″	W					

	T19				
C18	H6	H3′	H4′	H5′	H5″
H1′	VW	W	W	M	M
H2′	W				
H2″	W				
H3′	W				
H4′	W				
H5'	W				
H5″	W				

	G12			G20
T19	H1	H1′	H8	H8
H6	VW			W
Me		M	W	
H1′	M			M
H2′	VW			M
H2″	W			M
H3′				W
H4′				W

Note: M = medium intensity, red marked, W = weak intensity, VW = very weak intensity, OL = overlapped cross-peak.

Supplementary Table 6. Antibodies used in this study.

Antibody	Source	Catalog number	Application
Actin	Proteintech	No. 66009-1-Ig	WB: 1:1000
MET	Cell Signaling Technology	No. 8198S	WB: 1:1000<br>IHC: 1:200
LRPPRC	Abcam	No. ab259927	WB: 1:1000<br>ChIP: 1:25
Biotin-G4P	Nanjing Jietai		ChIP: 1:50
Goat anti-mouse IgG	Sigma-Aldrich	AP124	CUT&Tag: 1:100
Anti-FLAG antibody	Sigma-Aldrich	F1804	CUT&Tag: 1:100
HRP-conjugated Goat anti-Rabbit IgG (H+L)	Abclonal	No. AS014	WB: 1:2000
HRP-conjugated Goat anti-Mouse IgG (H+L)	Abclonal	No. AS003	WB: 1:4000
Ki67	Proteintech	No. 27309-1-AP	IHC: 1:2000
