# Supplementary material for: Molecular glue that stabilizes the LRPPRC−MET-G4 interaction complex to drive MET downregulation
Source: Nat Commun. 2026 Jun 4;17:7184. doi: 10.1038/s41467-026-73806-6 (PMC13396614; doi:10.1038/s41467-026-73806-6)
Supplement: Supplementary file 2 — Reporting Summary [file 41467_2026_73806_MOESM2_ESM.pdf]

nature portfolio

Corresponding author(s): Jin-Lei Bian, Hao Zhang, Kai-Bo Wang and Ling-Yi Kong

Last updated by author(s): May 5, 2026

Reporting Summary

Nature Portfolio wishes to improve the reproducibility of the work that we publish. This form provides structure for consistency and transparency in reporting. For further information on Nature Portfolio policies, see our Editorial Policies and the Editorial Policy Checklist.

Statistics

For all statistical analyses, confirm that the following items are present in the figure legend, table legend, main text, or Methods section.

n/a	Confirmed	
☐	☒	The exact sample size (*n*) for each experimental group/condition, given as a discrete number and unit of measurement
☐	☒	A statement on whether measurements were taken from distinct samples or whether the same sample was measured repeatedly
☐	☒	The statistical test(s) used AND whether they are one- or two-sided<br>*Only common tests should be described solely by name; describe more complex techniques in the Methods section.*
☒	☐	A description of all covariates tested
☐	☒	A description of any assumptions or corrections, such as tests of normality and adjustment for multiple comparisons
☐	☒	A full description of the statistical parameters including central tendency (e.g. means) or other basic estimates (e.g. regression coefficient) AND variation (e.g. standard deviation) or associated estimates of uncertainty (e.g. confidence intervals)
☐	☒	For null hypothesis testing, the test statistic (e.g. *F*, *t*, *r*) with confidence intervals, effect sizes, degrees of freedom and *P* value noted<br>*Give P values as exact values whenever suitable.*
☒	☐	For Bayesian analysis, information on the choice of priors and Markov chain Monte Carlo settings
☒	☐	For hierarchical and complex designs, identification of the appropriate level for tests and full reporting of outcomes
☒	☐	Estimates of effect sizes (e.g. Cohen's *d*, Pearson's *r*), indicating how they were calculated

Our web collection on statistics for biologists contains articles on many of the points above.

Software and code

Policy information about availability of computer code

Data collection	Topspin 4.1.1 (Bruker), SpectraManager (Jasco), Image Lab 6.1, Gaussian09, Amber 20, AlphaFold 3.0, CytoFLEX S, Lightcycler 480
Data analysis	Topspin 4.1.1 (Bruker),Image Lab 6.1, chemDraw 18.2, Pymol 2.3.2, VMD 1.9.4, Xplor-NIH 2.48, NMRFAM-SPARKY, GraphPad prism 8.2, Flowjo 10.8

For manuscripts utilizing custom algorithms or software that are central to the research but not yet described in published literature, software must be made available to editors and reviewers. We strongly encourage code deposition in a community repository (e.g. GitHub). See the Nature Portfolio guidelines for submitting code & software for further information.

Data

Policy information about availability of data

All manuscripts must include a data availability statement. This statement should provide the following information, where applicable:
- Accession codes, unique identifiers, or web links for publicly available datasets
- A description of any restrictions on data availability
- For clinical datasets or third party data, please ensure that the statement adheres to our policy

Source data are provided with this paper. The data that support the finding of this study are available from the corresponding authors upon reasonable request. The coordinates and experimental details generated in this study have been deposited in the Protein Data bank under accession codes 9JI9.

Research involving human participants, their data, or biological material

Policy information about studies with human participants or human data. See also policy information about sex, gender (identity/presentation), and sexual orientation and race, ethnicity and racism.

Reporting on sex and gender	*Use the terms sex (biological attribute) and gender (shaped by social and cultural circumstances) carefully in order to avoid confusing both terms. Indicate if findings apply to only one sex or gender; describe whether sex and gender were considered in study design; whether sex and/or gender was determined based on self-reporting or assigned and methods used. Provide in the source data disaggregated sex and gender data, where this information has been collected, and if consent has been obtained for sharing of individual-level data; provide overall numbers in this Reporting Summary. Please state if this information has not been collected. Report sex- and gender-based analyses where performed, justify reasons for lack of sex- and gender-based analysis.*
Reporting on race, ethnicity, or other socially relevant groupings	*Please specify the socially constructed or socially relevant categorization variable(s) used in your manuscript and explain why they were used. Please note that such variables should not be used as proxies for other socially constructed/relevant variables (for example, race or ethnicity should not be used as a proxy for socioeconomic status). Provide clear definitions of the relevant terms used, how they were provided (by the participants/respondents, the researchers, or third parties), and the method(s) used to classify people into the different categories (e.g. self-report, census or administrative data, social media data, etc.) Please provide details about how you controlled for confounding variables in your analyses.*
Population characteristics	*Describe the covariate-relevant population characteristics of the human research participants (e.g. age, genotypic information, past and current diagnosis and treatment categories). If you filled out the behavioural & social sciences study design questions and have nothing to add here, write "See above."*
Recruitment	*Describe how participants were recruited. Outline any potential self-selection bias or other biases that may be present and how these are likely to impact results.*
Ethics oversight	*Identify the organization(s) that approved the study protocol.*

Note that full information on the approval of the study protocol must also be provided in the manuscript.

Field-specific reporting

Please select the one below that is the best fit for your research. If you are not sure, read the appropriate sections before making your selection.

[x] Life sciences [ ] Behavioural & social sciences [ ] Ecological, evolutionary & environmental sciences

For a reference copy of the document with all sections, see nature.com/documents/nr-reporting-summary-flat.pdf

Life sciences study design

All studies must disclose on these points even when the disclosure is negative.

Sample size	Based on previous work, for in vivo experiments, mice were distributed into cohorts with 8 mice per cohort, is enough to observe a > 1.5-fold difference with 90% confidence.
Data exclusions	No data were excluded from the analysis.
Replication	Cell assays and molecular experiments were performed in at least biological triplicates.
Randomization	Mice for in vivo experiments were randomly assigned into cohorts. For other experiments, no randomization.
Blinding	Blinding was not deemed necessary.

Reporting for specific materials, systems and methods

We require information from authors about some types of materials, experimental systems and methods used in many studies. Here, indicate whether each material, system or method listed is relevant to your study. If you are not sure if a list item applies to your research, read the appropriate section before selecting a response.

Materials & experimental systems

n/a	Involved in the study
☐	☒ Antibodies
☐	☒ Eukaryotic cell lines
☒	☐ Palaeontology and archaeology
☐	☒ Animals and other organisms
☒	☐ Clinical data
☒	☐ Dual use research of concern
☒	☐ Plants

Methods

n/a	Involved in the study
☒	☐ ChIP-seq
☐	☒ Flow cytometry
☒	☐ MRI-based neuroimaging

Antibodies

Antibodies used	anti-β-actin (66009-1-Ig, proteintech, WB:1:1000, lot： 10038080), anti-MET (8198S, Cell Signaling Technology, WB: 1:1000, IHC: 1:200, lot:13), anti-LRPPRC (ab259927, abcam, lot: GR3406185-3), anti-Ki67 (27309-1-AP, Proteintech, IHC: 1:2000), Goat anti-mouse IgG (AP124, Sigma Aldrich, Cut&tag: 1:100), Anti-FLAG antibody (F1804, Sigma Aldrich, Cut&tag: 1:100), HRP-conjugated Goat anti-Rabbit IgG (H+L) (AS014, abclonal, WB: 1:2000), HRP-conjugated Goat anti-Mouse IgG (H+L) (AS003, Abclonal, WB: 1:4000)
Validation	All primary and secondary antibodies use in this study are commercially available and have been validated for used applications in human cells by the manufacturers. We have used recommended antibody dilutions for western blot experiments, and detected bands of expected molecular weight, as described by the manufacturers.

Eukaryotic cell lines

Policy information about cell lines and Sex and Gender in Research

Cell line source(s)	All of the cells lines were purchased from Shanghai Cell Bank of the Chinese Academy of Sciences .
Authentication	All of the cell lines used were authenticated by Shanghai Cell Bank of the Chinese Academy of Sciences.
Mycoplasma contamination	All cell lines have been routinely tested for mycoplasma contamination by a gPCR based assay and tested negative.
Commonly misidentified lines (See ICLAC register)	No commonly misidentified cell lines were used in this study.

Animals and other research organisms

Policy information about studies involving animals; ARRIVE guidelines recommended for reporting animal research, and Sex and Gender in Research

Laboratory animals	4-week-old male Balb/c nude mice were purchased from Hangzhou Ziyuan Laboratory Animal Technology Co., Ltd.
Wild animals	This study did not involve wild animals.
Reporting on sex	These results apply only to males.
Field-collected samples	The study did not involve samples collected from the field.
Ethics oversight	All animal care and experimental procedures were approved by the University Committee on Use and Care of Animals of the China Pharmaceutical University (2022-03-024 and YSL-202512002).

Note that full information on the approval of the study protocol must also be provided in the manuscript.

Plants

Seed stocks	*Report on the source of all seed stocks or other plant material used. If applicable, state the seed stock centre and catalogue number. If plant specimens were collected from the field, describe the collection location, date and sampling procedures.*
Novel plant genotypes	*Describe the methods by which all novel plant genotypes were produced. This includes those generated by transgenic approaches, gene editing, chemical/radiation-based mutagenesis and hybridization. For transgenic lines, describe the transformation method, the number of independent lines analyzed and the generation upon which experiments were performed. For gene-edited lines, describe the editor used, the endogenous sequence targeted for editing, the targeting guide RNA sequence (if applicable) and how the editor was applied.*
Authentication	*Describe any authentication procedures for each seed stock used or novel genotype generated. Describe any experiments used to assess the effect of a mutation and, where applicable, how potential secondary effects (e.g. second site T-DNA insertions, mosiacism, off-target gene editing) were examined.*

Flow Cytometry

Plots

Confirm that:

- [x] The axis labels state the marker and fluorochrome used (e.g. CD4-FITC).
- [x] The axis scales are clearly visible. Include numbers along axes only for bottom left plot of group (a 'group' is an analysis of identical markers).
- [x] All plots are contour plots with outliers or pseudocolor plots.
- [x] A numerical value for number of cells or percentage (with statistics) is provided.

Methodology

Sample preparation	Cell apoptosis was quantified by using the Cell Apoptosis Kit with Annexin V-fluorescein isothiocyanate (FITC) and propidium iodide (PI) (BD), according to the manufactures's instructions. Briefly, the testing cells were harvested 48-h post-treatment and washed with cold PBS. Cell density was adjusted to 1×10^6 cells/mL in 1 × annexin-binding buffer and stained with Annexin V-FITC and PI for 15 min at room temperature. The apoptosis rate of stained cells was determined using Flow cytometry (BECKMAN COULTER) with excitation at 488 nm. The emission wavelengths were set at 523 nm for Annexin V-FITC and 617 nm for PI.<br>Cell cycle was quantified by using the propidium iodide (PI) (BD), according to the manufactures's instructions. Briefly, the testing cells were harvested 48-h post-treatment and washed with cold PBS. Cell density was adjusted to 1×10^6 cells/mL in PBS and stained with PI for 15 min at room temperature. The cell cycle of staining cells was determined using Flow cytometry (BECKMAN COULTER).
Instrument	Flow cytometry (BECKMAN COULTER)
Software	Flowjo 10.8.1
Cell population abundance	Cell density was adjusted to 1×10^6 cells/mL
Gating strategy	A standard protocol was employed to set the gating. After using unstained controls to identify autofluorescence, single-stained controls for PI and Annexin V-FITC were used to set the compensation matrix, and the boundary between the normal, apoptotic and necrotic cells was confirmed by comparing the double-stained controls. Using this Annexin V/PI apoptotic detecting method, flow cytometry categorizes the Annexin V-/PI+ population as necrotic cells, the Annexin V+/PI+ populationas and the Annexin V+/PI- population as apoptotic cells.

- [x] Tick this box to confirm that a figure exemplifying the gating strategy is provided in the Supplementary Information.
