# Supplementary material for: Molecular glue that stabilizes the LRPPRC−MET-G4 interaction complex to drive MET downregulation
Source: Nat Commun. 2026 Jun 4;17:7184. doi: 10.1038/s41467-026-73806-6 (PMC13396614; doi:10.1038/s41467-026-73806-6)
Supplement: Supplementary file 3 — Transparent Peer Review file [file 41467_2026_73806_MOESM3_ESM.pdf]

Peer Review File

Molecule Glue That Stabilizes the LRPPRC–MET-G4 Interaction Complex to Drive MET Downregulation

Corresponding Author: Professor Ling-Yi Kong

This file contains all reviewer reports in order by version, followed by all author rebuttals in order by version.

Version 0:

Reviewer comments:

Reviewer #1

(Remarks to the Author)
The study by Yi-Pu Li characterizes a G-quadruplex (G4) epigenetic regulatory element, previously identified within the MET oncogene promoter, by providing its high-resolution NMR structure. The study identifies the LRPPRC protein as a MET-G4 interactor that promotes MET transcription and map its MET-G4 binding domain. Through a screening approach, the study then identifies the alkaloid nitidine (NIT) as a molecular glue capable of stabilizing the LRPPRC-NIT-MET-G4 ternary complex, thereby preventing MET transcription. Finally, the authors attempt to provide preclinical evidence for the potential application of NIT in downregulating MET expression and its oncogenic activity, using a hepatocellular carcinoma cell line (Hep G2) both in vitro and in vivo.
The characterization of the MET-G4 promoter structure and its interaction with LRPPRC is novel, interesting and methodologically sound (as far as can be judged by a non-expert in structural studies). Unfortunately, the part of the work addressing therapeutic application is weak and fails to provide convincing evidence that NIT-mediated transcriptional downregulation of MET could be a viable strategy. The current experimental design suffers from major flaws that preclude publication. These include:
(a) a lack of demonstration that interference with the LRPPRC-MET-G4 complex lead to actual MET protein downregulation in several experiments;
(b) the use of a model (HepG2) known to express only modest levels of MET, levels that are not driven by MET gene amplification and are therefore unlikely to significantly contribute to tumorigeneses, rendering HepG2 an inappropriate model for MET pharmacological targeting;
(c) use of nitidine, which is already known to exert antitumor effect by various mechanisms (see for example PMID: 33024576), without appropriate controls to exclude MET-indipendent nitidine effects, particularly in the in vivo experiments (e.g. by using tumor cells lacking MET expression);
(d) the absence of comparative experiments with a suitable positive control drug for MET inhibition, such as a clinically approved small-molecule kinase inhibitor like tepotinib o capmatinib. However, it should be noted that, in tumors formed by HepG2 cells, these inhibitors, despite having favorable farmacokinetics and in vivo tumor penetration, are expected to be ineffective, as MET is not a therapeutically relevant target in this context.
It must be emphasized that any claim of therapeutic efficacy for a new MET inhibitor must be evaluated in models that demonstrate dependence on MET oncogene activation. Such dependence is defined by specific biomarkers, including specific MET gene mutations (particularly MET exon 14 skipping) and MET gene amplification. These genetic alterations are essential predictors of inhibitor effectiveness, both in preclinical models and in clinical settings, as demonstrated in several studies (see, among others: PMID: 40679792; PMID: 29674709; PMID: 32532746). Importantly, these biomarkers are relevant not only for agents that directly target the MET protein -such as small-molecule tyrosine kinase inhibitors and monoclonal/engineered antibodies directed against the MET extracellular domains- but also for agents that act indirectly by suppressing MET protein expression.
In the introduction, the authors note that current MET inhibitors are poorly effective in ‘patients with MET gene amplification and protein overexpression” (line 65), implying the need for alternative approaches that interfere at transcriptional level. However, this statement overlooks an important diagnostic issue: the lack of clinical response in such patients is often due not to lack of inhibitor efficacy against the overexpressed protein, but to inaccurate identification of MET amplification. In many cases, copy number gains or overexpression measured by IHC are incorrectly interpreted as gene amplification, a common technical issue in molecular diagnostics. In contrast, truly MET-amplified tumors represent a biomarker-defined subset that does respond to MET inhibitors (PMID: 40679792; PMID: 29674709; PMID: 32532746). This principle is consistent with other receptor tyrosine kinases such as HER2, where only true amplification – not mere overexpression –

predicts responses in breast, colorectal, or gastric cancer (PMID: 29659677). Although most of these principles have been established for tumors with relatively high frequency of MET alterations (for which the inhibitors are approved), there is no biological rationale to assume that hepatocellular carcinoma (HCC) is governed by fundamentally different rules. It remains unlikely that HCC exhibit MET pathway dependence in the absence of genetic alterations that cause pathway dysregulation, such as true high-level MET amplification or activating mutations.

Other major concerns:
1.
Figure 3b. Evidence for MET downregulation by LRPPRC silencing is weak. To corroborate this finding (a) an additional siRNA targeting LRPPRC should be tested to rule out off-target effects; (b) given that MET mRNA levels are reduced by only ~50%, and considering the complexity of transcriptional and translational regulation of MET, it is essential to demonstrate effects at the protein level; (c) experiments should be replicated in at least one additional cell line to confirm generalizability. Moreover, the sequence of the control siRNA is missing from the corresponding table and should be provided.
2.
Fig. 3d. Based on the description, the proliferation assay appears to be conducted in the absence of HGF. Since HepG2 cells express wild-type MET, which is not constitutively active and requires HGF for activation, any observed anti-proliferative effect following LRPPRC silencing cannot be attributed to inhibition of MET expression and signaling. Instead, it is more likely due to the multiple other functions of LRPPRC, which extend beyond MET regulation.

Reviewer #2

(Remarks to the Author)
In the manuscript titled “Molecule glue that stabilizes the LRPPRC-MET-G4 interaction complex to facilitate MET downregulation”, the authors use a variety of biophysical and molecular biology techniques to reveal a novel epigenetic regulatory mechanism for regulating the expression of the MET through its promoter G-quadruplex element and the protein LRPPRC. Moreover, an in-house screen was performed to identify a small molecule alkaloid, nitidine, that was shown to directly bind the MET G4 and suppress MET expression in cells and in vivo through a mechanism of stabilizing the ternary LRPPRC-MET-G4 complex. This is the first such example of a protein-G4 molecular ‘glue’ molecule reported to date. The work is overall highly significant as it establishes a potentially new mechanism of gene down-regulation through protein-G4 ternary complex targeting. I recommend publication after the minor revisions below are addressed.
Significance
Overall, this work is highly significant as it provides the first in vivo evidence of protein-G4 molecular ‘glue’ molecules, and so has major implications for the further development of such molecules for additional targets – such as the ‘undruggable’ cMYC in cancer.
Validity
In general, the interpretation of the in vitro binding experiments, NMR structure determination, and molecular dynamics appear to be valid and appropriately interpreted. However, no details of sample concentration or identity (13C/15N?) of the oligonucleotide used in solving the NMR structure are given. Was the assignment made at natural abundance? The details of how the HADDOCK docking was performed is also severely lacking. Were restraints used? If so, is there NOESY data that can be included to support the ternary complex? Moreover, there is very little structural data supporting the ternary complex (e.g., NOESY data showing NIT binding to MET G4, chemical shifts due to LRPPRC domain binding to the NIT/MET G4 complex, etc.).
Data and methodology
The ChIP PCR and in vivo data are outside of my expertise. The quality of the data and presentation of binding data, cell death, NMR, MD, and MST binding data are adequate. There is some NMR data that is lacking: the NMR structural assignment section of the results refers to a “supplementary fig 2d” that supposedly shows internal guanine imino protection by NMR – this should be included.
Analytical Approach
My expertise is in biomolecular structure and interactions. I don’t see any major flaws in the analytical approaches to these techniques.
Suggested improvements
As mentioned above, adding specific details of how the HADDOCK docking was performed, including the imino protection data from NMR experiments, include any additional information to confirm (1) NIT binding site/mode from NOESY data and (2) any additional interaction data from NMR showing direct influence of MET G4 binding to the LRPPRC protein domain. Additionally, although this is a major advancement in the field (G4 molecular glues), is there any detailed analysis of the specificity of binding of NIT to the MET G4 over other G4s? For instance, another isoquinoline alkaloid, berberine, has been demonstrated to be highly promiscuous in its binding. Similarly, many G4 binding proteins (e.g., nucleolin) are fairly non-specific in their binding mode. Any additional specificity information should be included or at least a discussion should be provided as to how specificity might be developed.
Clarity and context
The manuscript is well-written, clear, and concise. See previous note for context.

Reviewer #3

(Remarks to the Author)

Li et al. present a significant finding regarding the molecular basis of inhibiting tumor progression by downregulating MET expression with robust in vivo and in vitro data. Firstly, high-resolution NMR studies comfirmed a novel parallel G-quadruplex (G4) topology within the MET proximal promoter region. Secondly, a new regulatory mechanism was uncovered in the MET signaling pathway, where LRPPRC exhibited enhanced binding affinity with the MET-G4 to promote MET expression. Thirdly, a natural product, nitidine alkaloid (NIT), was screened as a binder to stabilize the MET-G4 structure. Dual-luciferase reporter assays identified that NIT could suppress MET expression via a MET-G4-dependent mechanism in cancer cells. Furthermore, NIT functions as a glue to facilitate the formation and stability of the LRPPRC-NIT-MET-G4 ternary complex.
The manuscript is written clearly and builds on the earlier work of the author group on the interactions between oncogene G4 DNA and natural product alkaloids. Identifying the formation of the stable ternary complex is interesting, but also challenging. Some more experimental details and supplementary validation should be provided. While the MET-G4 structure is robustly validated via comprehensive NMR data, the MET-G4-NIT complex characterization relies solely on 1D NMR spectra. The lack of some supporting experiments seems necessary and the suggestions are described below.
Direct evidence for the formation of ternary complexes is still lacking. Modulating protein-protein interactions (PPIs) is an attractive strategy in drug discovery. When a molecular glue binds to its target, the resulting binary complexes create a temporarily specific surface or pocket on the distorted surface that facilitates guest protein binding, ultimately forming a ternary complex (Solomentsev et al. Biochemistry 2018, 57, 1451; Ryu et al. ACS Nano 2024, 18, 31451.). Hence, precise characterization of specific binding surface and conformational changes is of great importance for PPI modulation by molecular glues. Current ECD spectra analysis showed that the parallel topology of MET-G4 was maintained when it formed a complex with nitidine. However, supplementary structural elucidation is warranted, and we propose implementing techniques, such as native mass spectrometry to evaluate ternary complex structures (Sternicki et al. ChemMedChem. 2021, 16, 2206; Beveridge et al. ACS Cent Sci. 2020, 6, 1223.). The detailed molecular interaction mechanism needs to be described including the binding ternary interface residue contacts.

General comments:
Please provide complete 1D NMR spectra of MET-G4s without and with NIT and entire 2D NOESY spectra of MET-G4s in SI.
In Extended Data Fig. 2a, why does the biotin-labeled MET-G4-MUT sequence with non-G4 structures exhibit a positive band at around 280 nm?
RT-qPCR experiments showed that PABPC3 knockdown had an opposite effect. How to explain this?
On page 13, LRPPRC1025-1394 displayed similar binding affinities for two MET-G4 variants (Pu25m1T and Pu36 DNAs with different flanking lengths, Fig. 3f, g). The discussion conflicts with the EMSA gel results depicted in Fig. 3g, which show EMSA gel results of LRPPRC1025-1394 binding to FAM-labeled Pu25m1T or Pu25m1T-MUT.
On page 14, as a control, the MET-G4-MUT sequence still has a relative high affinity with a Kd value of 342.8 nM. What potential factors could account for this outcome?
On page 14, molecular docking between the LRPPRC1034-1394 domain and the determined MET-G4 structure was carried out using HADDOCK 2.4. The discussion is inconsistent with what Experimental Section (The lowest-energy LRPPRC1025-1394-MET-G4 complex structure) shows.
FRET-melting assay using the increase of 25oC in Tm values as a hit for the screening of MET-G4-binders from a natural product library. What considerations is it for?
Natural benzophenanthridine alkaloids, like NIT, have large planer π-aromatic surface responsible for the stacking interaction with G4 DNA. Prior studies showed their binding to G4-forming promoter sequences of various oncogenes (e.g., BCL2, VEGFA and KRAS) (Bessi et al. ACS Chem. Biol. 2012, 7, 1109; Arora et al. FEBS J. 2008, 275, 3971; Bhadra et al. Biochim. Biophys. Acta 2011, 1810, 485; Bazzicalupi et al. Nucleic Acids Res. 2013, 41, 632.). Chelerythrine, an analogue of NIT, can bind multiple oncogene promoter G4s and downregulate their expression (Jagannath et al. Scientific Reports 2017, 7, 40706). Can authors provide evidence that NIT binds specifically MET-G4?
DMSO served as the solvent for the ligand solution. It is worth considering that the addition of DMSO could disrupt MET-G4 formation. It is not clear from the current results whether the author has taken this into consideration.
DNA polymerase stop assay demonstrated the ability of NIT to stabilize MET-G4 structure in long DNA contexts and thereby inhibit DNA replication. However, to confirm the cell-line specificity, the cytotoxicity of NIT to normal human cells should be tested.

Reviewer #4

(Remarks to the Author)
The work by Ling-Yi Kong and group is an excellent research in G-quadruplex research in this specified domain. I am really very happy to review this paper. MET is an excellent target in several cancers. The promoter G quadruplex structure of it where the LRPPRC protein is binding is an hotspot to target the gene to repress its transcriptional fate. Nitidine as a molecular glue bridge the G-Quadruplex and the said protein. This cultivation is new in the field. This complex (MET-GQ-NITIDINE_LRPPRC) alters the structure and function of LRPPRC, ultimately leading to MET downregulation and tumor suppression. An array of high resolution NMR for G4 structure calculation, biophysical, in cellulo, and animal studies very well established that NITIDINE can be employed in hepatocellular carcinoma to target MET.
The work sounds very good and I recommend publication of the work in Nature Communication after revision.

Major points to be added and explained.

1. I recommend BG4 chip experiment in Hep-G2 cell to be performed to find out the expression of G4 in the promoter of MET gene.

2. Please show affinities of Nitidine with other G-quadruplex structures as control, Find out the Kds using spectroscopic or calorimetric approach.

Version 1:

Reviewer comments:

Reviewer #1

(Remarks to the Author)
The revised manuscript failed to convincingly address several of my criticisms.

POINT N. 1
In my referee report on the first version of the manuscript, in the General Comments, I noted (a) the lack of a clear demonstration that interference with the LRPPRC–MET–G4 complex leads to actual MET protein downregulation in several experiments. Linked to this general observation, I raised, as major concern No. 1 related to Figure 3b, that: “Evidence for MET downregulation by LRPPRC silencing is weak. To corroborate this finding (a) an additional siRNA targeting LRPPRC should be tested to rule out off-target effects; (b) given that MET mRNA levels are reduced by only ~50%, and considering the complexity of transcriptional and translational regulation of MET, it is essential to demonstrate effects at the protein level; (c) experiments should be replicated in at least one additional cell line to confirm generalizability”.

In response to my criticisms, the authors added experiments with an additional siRNA targeting LRPPRC in HepG2 (Extended Data 3d–e) and replicated the experiment in the HCCLM3 cell line. These experiments clearly demonstrate that, although LRPPRC was correctly downregulated in both cell lines together with MET mRNA, the final impact on MET protein is negligible (contrary to what is reported by the authors at lines 245–258). Please note that the MET band decreased in the western blot shown in Extended Data 3g is the upper MET band, corresponding to the inactive intracellular precursor, whereas the mature MET protein is only negligibly reduced. Moreover, the proliferation assay shown in Extended Data 4h is meaningless, as it was performed in the absence of HGF, which is required to activate MET and induce MET-dependent proliferation.

By performing these assays in the absence of HGF, the authors also failed to take into account my major point No. 2, which was as follows: “Fig. 3d. Based on the description, the proliferation assay appears to be conducted in the absence of HGF. Since HepG2 cells express wild-type MET, which is not constitutively active and requires HGF for activation, any observed anti-proliferative effect following LRPPRC silencing cannot be attributed to inhibition of MET signaling. Instead, it is more likely due to the multiple other functions of LRPPRC, which extend beyond MET regulation.” Obviously, the requirement for HGF applies not only to HepG2 cells, but also to the other cell lines in which the experiments were replicated.
The results shown in the revised manuscript therefore argue against a prominent role of LRPPRC in regulating MET expression: the mRNA effect is irrelevant if it does not translate into an effect at the protein level.

POINT N. 2
In my referee report on the first version of the manuscript, General Comments point (c), I objected that the use of nitidine required appropriate controls, as it is already known that this compound exerts antitumor effects through various mechanisms (see, for example, PMID: 33024576). However, the results lacked appropriate controls to exclude MET-independent nitidine effects, particularly in the in vivo experiments (e.g., by using tumor cells lacking MET expression).

In their reply, the authors provide an answer that fails to address this point. First, they state that they conducted experiments in “HepG2 cells, a model of hyperactivated MET signalling”. This definition does not apply to HepG2, which expresses (as is well known from the literature and as shown by the authors in Extended Data Fig. 2) only modest levels of MET. Second, based on the new experiments added (Extended Data Fig. 9a–b), they conclude (in the reply) that “nitidine’s antiproliferative activity stems not only from MET signaling inhibition but also from complementary, MET-independent pathways, aligning with its established polypharmacological profile”. Thus, the new experiments *confirm my objection: nitidine is a non-specific compound. When administered to tumors, it is impossible to discriminate MET-mediated effects from other activities. This is a serious confounding factor that precludes rigorous preclinical—and, even more so, clinical—testing of the molecule as a novel anti-MET agent.*

Considering the lack of evidence that the nitidine target LRPPRC can meaningfully affect MET protein levels, and given that nitidine has numerous effects, one must also consider the possibility that the reduction of MET protein levels shown in Extended Data Fig. 11 results from mechanisms independent of stabilization of the LRPPRC–MET–G4 interaction.

In their reply to my comment on nitidine’s lack of specificity, the authors observe that “capmatinib, a clinically approved MET inhibitor that primarily inhibits phosphorylation of the MET oncoprotein, failed to show a clear concentration-dependent reduction in total MET expression”. It must be stressed that no protein downregulation is expected from tyrosine kinase inhibitors; this is neither expected nor required for clinical effectiveness. The authors then conclude that “mechanistically, although nitidine lacks intrinsic target selectivity, its biological activity becomes functionally selective in MET-amplified cancers: it preferentially binds to and stabilizes the LRPPRC–MET–G4 interaction complex in these cells, leading to coordinate downregulation of MET mRNA and protein levels. This G4-mediated mechanism underlies nitidine’s selective cytotoxicity against MET-amplified cancer cells”. These claims—which are particularly relevant for clinical translation and

therefore should be expressed with extreme caution—lack any supporting evidence.

In summary, this manuscript fails to show that LRPPRC protein, the proposed nitidine target, is responsible for any significant modulation of MET protein levels, and it provides evidence that nitidine can exert antitumor effects independently of MET targeting. At this stage, the biological evidence does not provide any proof of concept that nitidine can be proposed as a new strategy for MET inhibition with clinical translation potential. If the manuscript is accepted for publication based on positive comments by the other reviewers, claims contradicted by the experimental results must be removed from the manuscript, and an honest, rigorous critical assessment of the study limitations and of the translational potential of nitidine must be added.

Reviewer #2

(Remarks to the Author)
All of my question/comments were addressed.

Reviewer #3

(Remarks to the Author)
I have reviewed the revised version of the manuscript submitted for publication in Nature Communications. All changes were clearly tracked, and I compared this version with the previous one. It is an impressive work and the revised manuscript has been improved. I recommend the manuscript for publication, however, only after minor corrections.
The regulatory mechanism at the MET promoter inferred based on the RT-qPCR experimental results can be incorporated into the main text, which will facilitate the understanding of the G4-based regulation.
NIT has been thoroughly analyzed by biophysical, genetic, and pharmacological experiments and confirmed to have excellent binding ability for stabilizing the LRPPRC-MET-G4 complex. Unfortunately, the structure of the ternary MET-G4-NIT-LRPPRC complex could not be obtained through NOESY data. Any comparative analysis of NIT's binding behavior with its analogues, such as berberine, should be incorporated to discuss how to enhance the NIT's selective G4-based antitumor effects.

Reviewer #4

(Remarks to the Author)
The work entitled "Molecule Glue That Stabilizes the LRPPRC–MET-G4 Interaction Complex to Drive MET Downregulation" has been well revised.
The authors have satisfactorily answered all the questions in the revision.
I strongly recommend the publication of the article.

Version 2:

Reviewer comments:

Reviewer #1

(Remarks to the Author)
The experiments presented in the new extended Fig. 9a,b and in Supplementary Fig. 9f still lack an important control: a comparison of the effects of nitidine in the presence and absence of HGF. Indeed, my request was obviously not to replace the experiment performed in the absence of HGF with one performed in the presence of HGF, but rather to compare the effect of nitidine under both conditions, in order to assess the relative contribution of nitidine to the inhibition of HGF-dependent proliferative effects, as opposed to more general inhibitory effects.

Therefore, the issue of nitidine's MET-unrelated effects remains unresolved.

However, given the enthusiasm of the other reviewers and the more honest analysis of the study's limitations, the manuscript deserves publication without further experimental revision.

I strongly recommend rendering the abstract more balanced by:
1. Changing the statement "This complex likely alters the structure and function of LRPPRC, ultimately leading to MET downregulation and potent anticancer effects" (lines 29–30) to: "This complex likely alters the structure and function of LRPPRC, ultimately leading to MET downregulation and anticancer effects that add to those already reported for nitidine."
2. Deleting the word "mainly" (line 33) from the statement: "Additionally, comprehensive in vitro and in vivo experiments demonstrated that nitidine significantly inhibits tumor progression mainly through an LRPPRC–MET-G4-dependent mechanism."

Reviewer #3

(Remarks to the Author)
All comments have been addressed. I recommend the manuscript for publication.

Open Access *This Peer Review File is licensed under a Creative Commons Attribution 4.0 International License, which permits use, sharing, adaptation, distribution and reproduction in any medium or format, as long as you give appropriate credit to the original author(s) and the source, provide a link to the Creative Commons license, and indicate if changes were made.*
In cases where reviewers are anonymous, credit should be given to 'Anonymous Referee' and the source.
The images or other third party material in this Peer Review File are included in the article's Creative Commons license, unless indicated otherwise in a credit line to the material. If material is not included in the article's Creative Commons license and your intended use is not permitted by statutory regulation or exceeds the permitted use, you will need to obtain permission directly from the copyright holder.
To view a copy of this license, visit https://creativecommons.org/licenses/by/4.0/

Response to Reviewers

The following are our point-by-point responses to reviewers' comments.

Reviewer #1 (Remarks to the Author)

The study by Yi-Pu Li characterizes a G-quadruplex (G4) epigenetic regulatory element, previously identified within the MET oncogene promoter, by providing its high-resolution NMR structure. The study identifies the LRPPRC protein as a *MET-G4* interactor that promotes MET transcription and map its *MET*-G4 binding domain. Through a screening approach, the study then identifies the alkaloid nitidine (NIT) as a molecular glue capable of stabilizing the LRPPRC-NIT-*MET*-G4 ternary complex, thereby preventing MET transcription. Finally, the authors attempt to provide preclinical evidence for the potential application of NIT in downregulating MET expression and its oncogenic activity, using a hepatocellular carcinoma cell line (HepG2) both in vitro and in vivo.

The characterization of the *MET*-G4 promoter structure and its interaction with LRPPRC is novel, interesting and methodologically sound (as far as can be judged by a non-expert in structural studies). Unfortunately, the part of the work addressing therapeutic application is weak and fails to provide convincing evidence that NIT-mediated transcriptional downregulation of MET could be a viable strategy. The current experimental design suffers from major flaws that preclude publication. These include:

(a) a lack of demonstration that interference with the LRPPRC-*MET*-G4 complex leads to actual MET protein downregulation in several experiments;

Response: We appreciate the reviewer for these insightful comments. We agree that establishing a direct link between complex interference and MET protein downregulation is crucial to validating our model. In the revised manuscript, we have experimentally strengthened this causal relationship through the following approaches:

1. ChIP experiments: ChIP-qPCR analysis revealed specific enrichment of LRPPRC at the *MET* promoter G4-forming region in cells, and treatment with NIT significantly enhanced LRPPRC binding to this site, providing direct evidence for the functional relevance of the LRPPRC-(NIT)-*MET*-G4 interaction complex in cells (Page 23, line 506-511, Fig. 5l).
2. Biophysical experiments: In vitro biophysical assays, including EMSA, microscale thermophoresis (MST), pull-down coupled with Western Blotting, and drug affinity responsive target stability (DARTS), demonstrated that NIT binds to and stabilizes the LRPPRC–*MET*-G4 interaction complex (Page 22, line 472-485, Fig. 3h and Fig. 5g-j).
3. Correlative downregulation: Consistent with in vitro biophysical experiments, NIT treatment in cells induced a robust, dose-dependent reduction in both *MET* mRNA and protein levels, establishing a pharmacologically tractable link between complex stabilization and *MET* downregulation (Page 20, line 432-434 and 456-458, Fig. 5e and f, Supplementary Fig. 9c-e, and Extended Data Fig. 11b, c).

4. Genetic validation: siRNA-mediated knockdown of *LRPPRC* recapitulated the NIT treatment phenotype, significantly decreasing *MET* mRNA and protein levels, thereby confirming LRPPRC's essential and specific role in sustaining *MET* signaling pathway (Page 12, line 244-248 and 252-254, Extended Data Fig. 3d, 3e, and Extended Data Fig. 4f, g).
5. Cellular imaging support: Immunofluorescence staining with the G4-specific peptide G4P and Cy5-labeled *MET*-G4 forming sequence further demonstrated these findings: LRPPRC overexpression combined with NIT treatment synergistically increased *MET*-G4 signals in cells, further supporting that NIT binds to and stabilizes the LRPPRC-*MET*-G4 interaction complex in cells (Page 24, line 511-527, Extended Data Fig. 12).

Collectively, our biophysical, genetic, and pharmacological experiments demonstrate that stabilizing the LRPPRC-*MET*-G4 complex with a small-molecule glue robustly suppresses *MET* expression. To enhance clarity and logical coherence, we have reorganized these findings in the revised manuscript, explicitly linking each intervention to its functional consequence on *MET* expression.

(b) the use of a model (HepG2) known to express only modest levels of MET, levels that are not driven by *MET* gene amplification and are therefore unlikely to significantly contribute to tumorigeneses, rendering HepG2 an inappropriate model for MET pharmacological targeting;

Response: In response to your comment, we first determined *MET* expression levels across several hepatocellular carcinoma (HCC) cell lines (Extended Data Fig. 2a). Based on this data, we found that HepG2 has moderate basal *MET* expression (Page 10, line 216, Extended Data Fig. 2a), as you mentioned. However, the MET protein level in HepG2 cells is significantly higher than in normal human hepatocytes (WRL68 cells). Moreover, siRNA-mediated *MET* knockdown significantly inhibited HepG2 cell proliferation, especially under HGF stimulation, demonstrating functional MET dependency (Page 10, line 218-220, Extended Data Fig. 2b-e). Additionally, the G4-CUT&Tag experiments in HepG2 cells revealed a significant enrichment of G4 signals at the *MET* proximal promoter region (Page 10, line 220, Fig. 3b). Collectively, we validated HepG2 cells as a suitable model for *MET* pharmacological targeting in HCC cell lines, especially for *MET*-G4 targeting, although it has a moderate basal *MET* expression.

(c) use of nitidine, which is already known to exert antitumor effect by various mechanisms (see for example PMID: 33024576), without appropriate controls to exclude MET-indipendent nitidine effects, particularly in the in vivo experiments (e.g. by using tumor cells lacking MET expression);

Response: Thanks for the comments. We agree that nitidine has multiple targets in cells, whereas the current study focuses on elucidating the concept that small-molecule glue can stabilize the LRPPRC-*MET*-G4 interaction complex, thereby killing the cancer cells. To investigate MET's functional role in nitidine's anticancer activity, we employed HGF-stimulated HepG2 cells, a model of hyperactivated *MET* signaling. In this system, both siRNA-mediated *MET* knockdown and

nitidine treatment alone significantly suppressed cell proliferation; critically, their combination yielded a synergistic antiproliferative effect (Page 20, line 435-439, Extended Data Fig. 9a). This synergistic effect was consistently observed in HCCLM3 cells (Page 20, line 439-443, Extended Data Fig. 9b), indicating that nitidine's antiproliferative activity stems not only from *MET* signaling inhibition but also from complementary, *MET*-independent pathways, aligning with its established polypharmacological profile. Moreover, *LRPPRC* knockdown, whether applied alone or in combination with nitidine, produced comparable inhibitory effects on proliferation and MET protein inhibition in both HGF-stimulated HepG2 and HCCLM3 cells (Page 21, line 443-446, Supplementary Fig. 9f, g). Taken together, these data support an *MET*- and *LRPPRC*-dependent anticancer mechanism of nitidine.

Moreover, we investigated nitidine's effect on EBC-1 and H1993 lung cancer cells, both of which harbor genomic *MET* amplification and exhibit consequent MET protein overexpression (Page 21, line 454-456, Extended Data Fig. 11a). Nitidine markedly suppressed both *MET* mRNA and protein levels in these two cell lines (Page 21, line 456-458, Supplementary Fig. 10a,b and Extended Data Fig. 11b, c). In contrast, capmatinib, a clinically approved MET inhibitor that primarily inhibits phosphorylation of the MET oncoprotein, failed to show a clear concentration-dependent reduction in total MET expression. Its effects on total *MET* expression levels were minimal and inconsistent across cell lines (Page 21, line 458-462, Supplementary Fig. 10c-f). Moreover, the reduced potency of nitidine in MET-low expression H460 cells (Page 21, line 462-464, Supplementary Fig. 10g) further underscores its selectivity for MET-dependent contexts. Taken together, these findings demonstrated nitidine downregulates *MET* expression predominantly through a *MET*-G4-mediated mechanism, as validated in both liver and lung cancer cell models.

For the in vivo experiments, we extended our evaluation to H1993-derived xenografts (Page 25, line 547, Extended Data Fig. 14a). Herein, nitidine again significantly inhibited tumor growth and suppressed *MET* expression levels (Page 25, line 548, Extended Data Fig. 14b–f), achieving comparable antitumor activity compared to the clinically approved MET inhibitor of capmatinib. qPCR, Western Blotting, hematoxylin and eosin (H&E) staining, and immunohistochemical (IHC) analysis further validated the tumor inhibition effect of nitidine by downregulating *MET* expression (Page 25, line 549-553, Extended Data Fig. 14e-g).

With these new data, we demonstrate that nitidine exerts robust, G4-based antitumor effects across multiple cancer models by disrupting the LRPPRC-*MET*-G4 regulatory axis, offering a novel therapeutic strategy for *MET*-driven tumors, particularly those with *MET* gene amplification. Mechanistically, although nitidine lacks intrinsic target selectivity, its biological activity becomes functionally selective in *MET*-amplified cancers: it preferentially binds to and stabilizes the LRPPRC-*MET*-G4 interaction complex in these cells, leading to coordinated downregulation of *MET* mRNA and protein levels. This G4-mediated mechanism underlies nitidine's selective cytotoxicity against *MET*-amplified cancer cells.

(d) the absence of comparative experiments with a suitable positive control drug for MET inhibition, such as a clinically approved small-molecule kinase inhibitor like tepotinib or capmatinib. However, it should be noted that, in tumors formed by HepG2 cells, these inhibitors, despite having favorable farmacokinetics and in vivo tumor penetration, are expected to be ineffective, as MET is not a

therapeutically relevant target in this context.

Response: Thanks for the comments. As mentioned above, we extended our evaluation to H1993-derived xenografts, which harbor genomic *MET* amplification (Page 25, line 547, Extended Data Fig. 14a). Herein, nitidine again significantly inhibited tumor growth and suppressed *MET* expression levels (Page 25, line 548, Extended Data Fig. 14b–f), achieving comparable antitumor activity compared to the clinically approved MET inhibitor of capmatinib. qPCR, Western Blotting, hematoxylin and eosin (H&E) staining, and immunohistochemical (IHC) analysis further validated the tumor inhibition effect of nitidine by downregulating *MET* expression (Page 25, line 549-553, Extended Data Fig. 14e-g).

It must be emphasized that any claim of therapeutic efficacy for a new MET inhibitor must be evaluated in models that demonstrate dependence on *MET* oncogene activation. Such dependence is defined by specific biomarkers, including specific MET gene mutations (particularly MET exon 14 skipping) and MET gene amplification. These genetic alterations are essential predictors of inhibitor effectiveness, both in preclinical models and in clinical settings, as demonstrated in several studies (see, among others: PMID: 40679792; PMID: 29674709; PMID: 32532746). Importantly, these biomarkers are relevant not only for agents that directly target the MET protein -such as small-molecule tyrosine kinase inhibitors and monoclonal/engineered antibodies directed against the MET extracellular domains- but also for agents that act indirectly by suppressing MET protein expression.

In the introduction, the authors note that current MET inhibitors are poorly effective in ‘patients with MET gene amplification and protein overexpression” (line 65), implying the need for alternative approaches that interfere at transcriptional level. However, this statement overlooks an important diagnostic issue: the lack of clinical response in such patients is often due not to lack of inhibitor efficacy against the overexpressed protein, but to inaccurate identification of MET amplification. In many cases, copy number gains or overexpression measured by IHC are incorrectly interpreted as gene amplification, a common technical issue in molecular diagnostics. In contrast, truly MET-amplified tumors represent a biomarker-defined subset that does respond to MET inhibitors (PMID: 40679792; PMID: 29674709; PMID: 32532746). This principle is consistent with other receptor tyrosine kinases such as HER2, where only true amplification – not mere overexpression – predicts responses in breast, colorectal, or gastric cancer (PMID: 29659677). Although most of these principles have been established for tumors with relatively high frequency of MET alterations (for which the inhibitors are approved), there is no biological rationale to assume that hepatocellular carcinoma (HCC) is governed by fundamentally different rules. It remains unlikely that HCC exhibit MET pathway dependence in the absence of genetic alterations that cause pathway dysregulation, such as true high-level MET amplification or activating mutations.

Response: We sincerely thank the reviewer for these insightful and constructive comments. We fully agree that diagnostic limitations, particularly the lack of reliable biomarkers to identify patients most likely to benefit, are a major factor hindering the clinical efficacy of current MET protein-targeted therapeutics. However, we propose that therapeutic resistance may also arise from intrinsic biological mechanisms beyond patient selection. Specifically, in tumors with *MET* gene amplification, cancer cells often overexpress MET protein at levels that saturate available

therapeutic agents (e.g., small-molecule inhibitors or monoclonal antibodies). Under such conditions, residual unbound MET receptors continue to dimerize and activate downstream oncogenic signaling, leading to incomplete pathway suppression and eventual treatment failure. Our approach circumvents this limitation by targeting *MET* expression at the genetic level (*MET*-G4-mediated transcriptional repression), thereby reducing total MET protein burden, not just blocking its activity. This strategy is especially rational and promising for *MET*-amplified cancers, which constitute the primary focus of our study. We believe the newly generated experimental data, mechanistic analyses, and refined conceptual framework presented herein strengthen the novelty, rigor, and translational relevance of our work, and we hope they merit consideration for publication in ***Nature Communications***.

Other major concerns:

1. Figure 3b. Evidence for MET downregulation by LRPPRC silencing is weak. To corroborate this finding (a) an additional siRNA targeting LRPPRC should be tested to rule out off-target effects; (b) given that MET mRNA levels are reduced by only ~50%, and considering the complexity of transcriptional and translational regulation of MET, it is essential to demonstrate effects at the protein level; (c) experiments should be replicated in at least one additional cell line to confirm generalizability. Moreover, the sequence of the control siRNA is missing from the corresponding table and should be provided.

Response: The comments are accepted. We have employed a second, distinct siRNA targeting *LRPPRC* and observed consistent results (Page 11, line 235-239, Fig.3c). This decrease was subsequently confirmed at the protein level (Page 12, line 244-246, Extended Data Fig. 3e). Moreover, *MET* mRNA dropped to ~30% of control levels as early as 24 h post-knockdown (Page 12, line 244-246, Extended Data Fig. 3d). Importantly, all key findings, including *LRPPRC* knockdown efficiency, *MET* mRNA suppression, and MET protein reduction, were robustly reproduced in the highly metastatic HCCLM3 cell line (Page 12, line 252-254, Extended Data Fig. 4f, g).

2. Fig. 3d. Based on the description, the proliferation assay appears to be conducted in the absence of HGF. Since HepG2 cells express wild-type MET, which is not constitutively active and requires HGF for activation, any observed anti-proliferative effect following LRPPRC silencing cannot be attributed to inhibition of MET expression and signaling. Instead, it is more likely due to the multiple other functions of LRPPRC, which extend beyond MET regulation.

Response: Thanks for the comments. We have supplemented the study with cell viability assays in HepG2 cancer cells under HGF stimulation, followed by NIT treatment and *LRPPRC* knockdown. These new data have been included in the revised manuscript (page 20, line 435-447, Extended Data Fig. 9a, b, and Supplementary Fig. 9f).

Reviewer #2 (Remarks to the Author)

In the manuscript titled "Molecule glue that stabilizes the LRPPRC-*MET*-G4 interaction complex to facilitate MET downregulation", the authors use a variety of biophysical and molecular biology techniques to reveal a novel epigenetic regulatory mechanism for regulating the expression of the MET through its promoter G-quadruplex element and the protein LRPPRC. Moreover, an in-house screen was performed to identify a small molecule alkaloid, nitidine, that was shown to directly bind the MET G4 and suppress MET expression in cells and in vivo through a mechanism of stabilizing the ternary LRPPRC-*MET*-G4 complex. This is the first such example of a protein-G4 molecular 'glue' molecule reported to date. The work is overall highly significant as it establishes a potentially new mechanism of gene down-regulation through protein-G4 ternary complex targeting. I recommend publication after the minor revisions below are addressed.

Response: Thank you very much for your support of our findings, and we sincerely appreciate your insightful feedback.

Significance

Overall, this work is highly significant as it provides the first in vivo evidence of protein-G4 molecular 'glue' molecules, and so has major implications for the further development of such molecules for additional targets – such as the 'undruggable' cMYC in cancer.

Validity

In general, the interpretation of the in vitro binding experiments, NMR structure determination, and molecular dynamics appears to be valid and appropriately interpreted. However, no details of sample concentration or identity (13C/15N?) of the oligonucleotide used in solving the NMR structure are given. Was the assignment made at natural abundance? The details of how the HADDOCK docking was performed are also severely lacking. Were restraints used? If so, is there NOESY data that can be included to support the ternary complex? Moreover, there is very little structural data supporting the ternary complex (e.g., NOESY data showing NIT binding to MET G4, chemical shifts due to LRPPRC domain binding to the NIT/MET G4 complex, etc.).

Response: We sincerely thank the reviewer for raising this important point regarding the NMR sample preparation and the docking parameters. We apologize for the omission of these details in the initial manuscript, and we now provide them in the revised "Materials and Methods" section.

"NMR spectroscopy (Page 48, line 934)

All NMR experiments were performed using an unlabeled oligonucleotide sample at natural isotopic abundance (without ^{13}C or ^{15}N enrichment). For the 2D NOESY, COSY, and HSQC experiments, the DNA sample was prepared at a concentration of 1.51 mM in a 25 mM K^+-containing buffer (pH 7), with 90% H_2O/10% D_2O as the solvent. NMR spectra were collected using a Bruker AV-600 spectrometer equipped with a QCI Cryoprobe. The w5 water suppression was applied to all

experiments, unless otherwise indicated. NOESY experiments were conducted at 5, 15, 25, and 35 °C, with mixing times of 80, 300, 350, and 400 ms, respectively, and NS = 32 (32 scans per t_1 increment). The data matrix consisted of 4096 complex points in TD (F2) and 480 in TD (F1). The DQF-COSY spectrum was acquired at 25 °C, while ^{1}H-^{13}C HSQC spectra were obtained at both 25 °C and 35 °C. Chemical shift calibration was indirectly performed for ^{13}C relative to DSS and directly for ^{1}H based on the water signal relative to DSS. NMR spectral processing and analysis were performed using Topspin 3.5 (Bruker) and Sparky (UCSF) software, respectively.

Protein-G4 docking with HADDOCK2.4 web server (Page 50, line 970)

The structural model of $LRPPRC_{1034\text{-}1394}$ was predicted using AlphaFold 3.0, while the solution NMR structure of the *MET*-G4 was determined experimentally in our lab. Protein-G4 docking was performed using the HADDOCK2.4 web server (EASY mode) with default parameters. Residues1034-1328 of LRPPRC were defined as active residues, and all residues of the *MET*-G4 were treated as active. Docking yielded 12 clusters, and the top-ranked model with the lowest binding-energy structure was selected for subsequent molecular dynamics simulation.

Unrestrained molecular dynamics (MD) Simulation (Page 50, line 979)

The top-ranked $LRPPRC_{1034\text{-}1394}$-*MET*-G4 complex structure from HADDOCK2.4 docking was neutralized with K^+ cations, two of which were explicitly positioned between adjacent G-tetrads. The system was solvated in a 10.0 Å truncated octahedral box of TIP3P water using tleap, and 150 mM K^+/Cl^- ions were added to mimic physiological ionic strength. The Amber OL15 and ff19SB force fields were used for DNA and protein, respectively. The initial equilibration protocol consisted of two energy minimization steps: first, 500 steps of steepest descent minimization; second, conjugate gradient minimization, both performed while applying positional restraints (force constant = 25 $kcal \cdot mol^{-1} \cdot Å^{-2}$) to the DNA backbone atoms. Subsequently, the system was gradually heated from 100 K to 300 K over 20 ps under NVT conditions. Under constant pressure and temperature control of 300 K, the final MD simulations were run using PMEMD CUDA Amber 20 for 1250 ns. Snapshots were taken every five picoseconds. No restraints were applied during the whole MD simulations. Trajectory analysis (last 1150 ns) employed VMD and PyMOL for structural visualization and RMSD calculation (all-atom RMSD relative to the top model). Binding free energy contributions per residue (amino acid and nucleotide) were calculated using MM/PBSA. Hierarchical agglomerative (bottom-up) clustering identified dominant conformational states, and representative average structures from each major cluster were used for final structural interpretation and presentation."

We regret that we were unable to acquire NOESY data for the ternary *MET*-G4-NIT-LRPPRC complex. Upon titration of NIT into *MET*-G4, 1D ^{1}H NMR spectra revealed pronounced broadening and substantial chemical shift perturbations of the imino proton signals (Page 18, line 378-381, Extended Data Fig. 8c and Supplementary Fig. 5), rather than the appearance of a sharp, well-resolved set of twelve new imino peaks expected for a single, structurally homogeneous bound state.

This behavior strongly indicates dynamic, multi-modal binding of NIT to *MET*-G4, involving rapid-to-medium exchange among several distinct interaction geometries, not a single, rigid binding pose. Under such conditions, a 2D NOESY experiment would yield numerous weak, broadened, or severely overlapped intermolecular NOE cross-peaks, precluding unambiguous assignment of binding site(s) and orientation(s). Moreover, incorporation of the large (~42 kDa), intrinsically dynamic LRPPRC protein fragment will further exacerbate spectral complexity: severe signal broadening and extensive peak overlap in both 1D and 2D spectra rendered conventional NMR characterization of the full ternary complex impractical. Consequently, direct evidence for ternary complex formation was instead established through a combination of biophysical, biochemical, and cellular approaches, as detailed in our response to Reviewer 1's Question 1.

Data and methodology

The ChIP PCR and in vivo data are outside of my expertise. The quality of the data and presentation of binding data, cell death, NMR, MD, and MST binding data are adequate. There is some NMR data that is lacking: the NMR structural assignment section of the results refers to a "supplementary fig 2d" that supposedly shows internal guanine imino protection by NMR – this should be included.

Response: Thanks for the comments. This is the solvent exchange assay, used to probe the internal protection of guanine imino protons located on the central G-tetrad through 1D NMR. The experimental data are now presented in Extended Data Figure 1b (page 9, lines 181-184, Extended Data Fig. 1b).

Analytical Approach

My expertise is in biomolecular structure and interactions. I don't see any major flaws in the analytical approaches to these techniques.

Suggested improvements

As mentioned above, adding specific details of how the HADDOCK docking was performed, including the imino protection data from NMR experiments, include any additional information to confirm (1) NIT binding site/mode from NOESY data and (2) any additional interaction data from NMR showing direct influence of *MET* G4 binding to the LRPPRC protein domain.

Additionally, although this is a major advancement in the field (G4 molecular glues), is there any detailed analysis of the specificity of binding of NIT to the MET G4 over other G4s? For instance, another isoquinoline alkaloid, berberine, has been demonstrated to be highly promiscuous in its binding. Similarly, many G4 binding proteins (e.g., nucleolin) are fairly non-specific in their binding mode. Any additional specificity information should be included or at least a discussion should be provided as to how specificity might be developed.

Response:

We thank the reviewer for the questions regarding the molecular docking and NMR studies. We have addressed these questions in the above section and hope the reviewer will be satisfied with our answers.

For the other questions, please see below.

The binding selectivity of NIT to other G4s

To determine the binding selectivity of NIT for the parallel *MET*-G4 relative to other G4 topologies, we performed CD and fluorescence experiments. The data demonstrated that NIT exhibits strong binding affinity for diverse G4 structures, as evidenced by substantial thermal stabilization (ΔT_m up to 26 °C) and tight binding (K_d values in the low nanomolar range) across parallel, antiparallel, and hybrid G4 topologies (Page18, line394-397, Supplementary Fig. 7, 8)

The binding selectivity of LRPPRC to other G4s

To characterize the binding selectivity of $LRPPRC_{1025\text{-}1394}$ for different types of G4 structures, we performed competitive EMSA experiments. In this assay, the binding activity of $LRPPRC_{1025\text{-}1394}$ to *MET*-G4 was competitively challenged with canonical G4s of parallel, hybrid, and antiparallel folding topologies. The data reveal that $LRPPRC_{1025\text{-}1394}$ exhibits selective binding affinity for parallel and hybrid G4 structures, with significantly weaker interaction toward antiparallel G4s (Page14, line294-310, Extended Data Fig. 5e).

As you suggested, we include a discussion about nitidine's selectivity on discussion part (Page 27, line 579-584).

"Strikingly, although nitidine lacks intrinsic target selectivity, its biological activity becomes functionally selective in *MET*-amplified cancers: it preferentially binds to and stabilizes the LRPPRC-*MET*-G4 interaction complex in these cells, leading to coordinated downregulation of *MET* mRNA and protein levels. This G4-mediated mechanism underlies nitidine's selective cytotoxicity against *MET*-amplified cancer cells."

Clarity and context

The manuscript is well-written, clear, and concise. See previous note for context.

Reviewer #3 (Remarks to the Author)

Li et al. present a significant finding regarding the molecular basis of inhibiting tumor progression by downregulating MET expression with robust in vivo and in vitro data. Firstly, high-resolution NMR studies confirmed a novel parallel G-quadruplex (G4) topology within the MET proximal promoter region. Secondly, a new regulatory mechanism was uncovered in the MET signaling pathway, where LRPPRC exhibited enhanced binding affinity with the *MET*-G4 to promote MET expression. Thirdly, a natural product, nitidine alkaloid (NIT), was screened as a binder to stabilize the *MET*-G4 structure. Dual-luciferase reporter assays identified that NIT could suppress MET expression via a *MET*-G4-dependent mechanism in cancer cells. Furthermore, NIT functions as a glue to facilitate the formation and stability of the LRPPRC-NIT-*MET*-G4 ternary complex.
The manuscript is written clearly and builds on the earlier work of the author group on the

interactions between oncogene G4 DNA and natural product alkaloids. Identifying the formation of the stable ternary complex is interesting, but also challenging. Some more experimental details and supplementary validation should be provided. While the *MET*-G4 structure is robustly validated via comprehensive NMR data, the *MET*-G4-NIT complex characterization relies solely on 1D NMR spectra. The lack of some supporting experiments seems necessary, and the suggestions are described below.

Direct evidence for the formation of ternary complexes is still lacking. Modulating protein-protein interactions (PPIs) is an attractive strategy in drug discovery. When a molecular glue binds to its target, the resulting binary complexes create a temporarily specific surface or pocket on the distorted surface that facilitates guest protein binding, ultimately forming a ternary complex (Solomentsev et al. Biochemistry 2018, 57, 1451; Ryu et al. ACS Nano 2024, 18, 31451.). Hence, precise characterization of specific binding surface and conformational changes is of great importance for PPI modulation by molecular glues. Current ECD spectra analysis showed that the parallel topology of *MET*-G4 was maintained when it formed a complex with nitidine. However, supplementary structural elucidation is warranted, and we propose implementing techniques, such as native mass spectrometry to evaluate ternary complex structures (Sternicki et al. ChemMedChem. 2021, 16, 2206; Beveridge et al. ACS Cent Sci. 2020, 6, 1223.). The detailed molecular interaction mechanism needs to be described including the binding ternary interface residue contacts.

Response: We sincerely thank the reviewer for these insightful comments.

Upon titration of NIT into *MET*-G4, 1D ^{1}H NMR spectra revealed pronounced broadening and substantial chemical shift perturbations of the imino proton signals (Page 18, line 378-381, Extended Data Fig. 8c and Supplementary Fig. 5), rather than the appearance of a sharp, well-resolved set of twelve new imino peaks expected for a single, structurally homogeneous bound state. This behavior strongly indicates dynamic, multi-modal binding of NIT to *MET*-G4, involving rapid-to-medium exchange among several distinct interaction geometries, not a single, rigid binding pose. Under such conditions, a 2D NOESY experiment would yield numerous weak, broadened, or severely overlapped intermolecular NOE cross-peaks, precluding unambiguous assignment of binding site(s) and orientation(s). Therefore, it is impractical to determine the *MET*-G4-NIT binding complex structure using NMR.

We agree that native mass spectrometry (MS) is a powerful and maybe an appropriate technique for characterizing ternary/binary G4 complexes involving small molecules and/or proteins. However, this approach is currently not feasible in our laboratory due to two key limitations: 1) the absence of a high-resolution native MS instrument capable of preserving non-covalent interactions under gentle ionization conditions; and 2) the lack of in-house expertise in native MS, particularly for G4-ligand-protein systems, which require specialized method development and interpretation. Furthermore, maintaining low-K^+ mobile phases, essential for stabilizing G4 structures during analysis, is incompatible with standard MS workflows in our facility, as K^+ adducts severely suppress ionization efficiency and compromise spectral quality. Given these technical constraints, we adopted complementary biophysical, biochemical, and cellular approaches independently confirm the formation of the ternary complex, as illustrated below:

1. ChIP experiments: ChIP-qPCR analysis revealed specific enrichment of LRPPRC at the

MET promoter G4-forming region in cells, and treatment with NIT significantly enhanced LRPPRC binding to this site, providing direct evidence for the functional relevance of the LRPPRC-(NIT)-*MET-G4* interaction complex in cells (Page 23, line 506-511, Fig. 5l).

2. Biophysical experiments: In vitro biophysical assays, including EMSA, microscale thermophoresis (MST), pull-down coupled with Western Blotting, and drug affinity responsive target stability (DARTS), demonstrated that NIT binds to and stabilizes the LRPPRC–*MET*-G4 interaction complex (Page 22, line 472-485, Fig. 3h and Fig. 5g-j).

3. Correlative downregulation: Consistent with in vitro biophysical experiments, NIT treatment in cells induced a robust, dose-dependent reduction in both *MET* mRNA and protein levels, establishing a pharmacologically tractable link between complex stabilization and *MET* downregulation (Page 20, line 432-434 and 456-458, Fig. 5e and f, Supplementary Fig. 9c-e, and Extended Data Fig. 11b, c).

4. Genetic validation: siRNA-mediated knockdown of *LRPPRC* recapitulated the NIT treatment phenotype, significantly decreasing *MET* mRNA and protein levels, thereby confirming LRPPRC's essential and specific role in sustaining *MET* signaling pathway (Page 12, line 244-248 and 252-254, Extended Data Fig. 3d, 3e, and Extended Data Fig. 4f, g).

5. Cellular imaging support: Immunofluorescence staining with the G4-specific peptide G4P and Cy5-labeled *MET*-G4 forming sequence further demonstrated these findings: LRPPRC overexpression combined with NIT treatment synergistically increased *MET*-G4 signals in cells, further supporting that NIT binds to and stabilizes the LRPPRC-*MET*-G4 interaction complex in cells (Page 24, line 511-527, Extended Data Fig. 12).

Collectively, our biophysical, genetic, and pharmacological experiments demonstrate that stabilizing the LRPPRC-*MET*-G4 complex with a small-molecule glue robustly suppresses *MET* expression. To enhance clarity and logical coherence, we have reorganized these findings in the revised manuscript, explicitly linking each intervention to its functional consequence on *MET* expression. We hope these data are sufficient to address the reviewer's questions.

General comments:

1. Please provide complete 1D NMR spectra of *MET*-G4s without and with NIT and entire 2D NOESY spectra of *MET*-G4s in SI.

   Response: The comment is accepted. We now provide complete 1D NMR spectra of *MET*-G4s without and with NIT (Supplementary Fig. 5), as well as entire 2D NOESY spectra of *MET*-G4s (Supplementary Fig. 1).

2. In Extended Data Fig. 2a, why does the biotin-labeled *MET*-G4-MUT sequence with non-G4 structures exhibit a positive band at around 280 nm?

   Response: Thanks for the comments. The *MET*-G4-MUT sequence predominantly adopts

single-stranded DNA conformations; the positive CD signal observed near 280 nm arises from intrinsic base stacking of nucleic acid residues in these single-stranded or partially folded DNA.

3. RT-qPCR experiments showed that PABPC3 knockdown had an opposite effect. How to explain this?

Response: Our findings reveal a nuanced regulatory mechanism at the *MET* promoter: its stable *MET*-G4 structure acts not as a static switch, but as a dynamic platform that recruits both transcriptional activators and repressors. Specifically, LRPPRC stabilizes the *MET*-G4 and promotes *MET* expression by facilitating assembly of activating complexes; in contrast, PABPC3 is recruited to the same *MET*-G4 and functions as a transcriptional repressor. Its knockdown increases *MET* expression, directly opposing the LRPPRC loss. This reciprocal phenotype is not contradictory; rather, it provides compelling functional evidence for a balanced, bi-functional G4 hub. PABPC3 likely suppresses transcription through one or more complementary mechanisms: a) direct recruitment of corepressors; b) steric interference with activator binding or function proteins, including displacement or occlusion of LRPPRC; or c) promotion of G4 resolution, thereby transiently establishing a repressive chromatin environment. Thus, the net *MET* transcriptional output reflects the real-time equilibrium between these antagonistic G4-bound regulators. Knocking down either factor perturbs this equilibrium, reducing LRPPRC, which favors repression, while depleting PABPC3 favors activation, functionally validating their opposing yet interdependent roles in fine-tuning *MET* gene expression. However, a definitive and accurate conclusion requires additional data; therefore, this discussion has been omitted from the main manuscript.

4. On page 13, $LRPPRC_{1025\text{-}1394}$ displayed similar binding affinities for two *MET*-G4 variants (Pu25m1T and Pu36 DNAs with different flanking lengths, Fig. 3f, g). The discussion conflicts with the EMSA gel results depicted in Fig. 3g, which show EMSA gel results of $LRPPRC_{1025\text{-}1394}$ binding to FAM-labeled Pu25m1T or Pu25m1T-MUT.

Response: We sincerely thank the reviewer for their careful reading and for pinpointing this source of confusion. We apologize for the inconsistency between the text and the figures 3f and 3g, which resulted from an oversight during manuscript preparation. Upon re-examining the relevant section, we have corrected and updated the relevant figures.

"Moreover, $LRPPRC_{1025\text{-}1394}$ displayed similar specific binding affinities for two *MET*-G4 variants (Pu25m1T and Pu36 DNAs with different flanking lengths, Fig. 3g and Extended Data Fig. 5c)"

5. On page 14, as a control, the *MET*-G4-MUT sequence still has a relative high affinity with a Kd value of 342.8 nM. What potential factors could account for this outcome?

Response: We thank the reviewer for raising this point. The observed binding affinity of $LRPPRC_{1025\text{-}1394}$ for the *MET*-G4-MUT sequence (K_d = 342.8 nM) likely reflects non-specific electrostatic interactions, arising from the positively charged protein surface and the negatively

charged single-stranded DNA control, rather than specific G4 structural recognition. Crucially, the affinity for the *MET*-G4 structure is markedly higher ($K_d \approx 69.6$ nM), representing a > 4.9-fold increase. This substantial difference provides robust biophysical evidence for selective recognition of the folded G4 architecture.

6. On page 14, molecular docking between the $LRPPRC_{1034-1394}$ domain and the determined *MET*-G4 structure was carried out using HADDOCK 2.4. The discussion is inconsistent with what Experimental Section (The lowest-energy $LRPPRC_{1025-1394}$-*MET*-G4 complex structure) shows.

   Response: Thank you for your careful review. We sincerely apologize for the error in the Experimental Section and have now corrected it in the revised manuscript.

7. FRET-melting assay using the increase of 25°C in Tm values as a hit for the screening of *MET*-G4-binders from a natural product library. What considerations is it for?

   Response: The primary goal of this screening assay was to identify natural products that potently stabilize the *MET*-G4. To prioritize high-confidence hits, we applied a stringent ΔT_m threshold of > 25 °C, selected to exclude moderate or weak binders, and focused exclusively on compounds with robust stabilization activity. A lower cutoff (e.g., > 5 °C or > 10 °C) would have yielded substantially more candidates, thereby expanding downstream validation efforts without commensurate gains in hit quality. Indeed, only eight compounds surpassed the 25 °C threshold, and all eight compounds displayed ΔT_m values more than 25 °C, making them the most potent and consistent stabilizers identified in the screen. This strategy balanced rigor with feasibility, delivering a compact, high-value set of leads for mechanistic and functional follow-up.

8. Natural benzophenanthridine alkaloids, like NIT, have large planar π-aromatic surface responsible for the stacking interaction with G4 DNA. Prior studies showed their binding to G4-forming promoter sequences of various oncogenes (e.g., BCL2, VEGFA and KRAS) (Bessi et al. ACS Chem. Biol. 2012, 7, 1109; Arora et al. FEBS J. 2008, 275, 3971; Bhadra et al. Biochim. Biophys. Acta 2011, 1810, 485; Bazzicalupi et al. Nucleic Acids Res. 2013, 41, 632.). Chelerythrine, an analogue of NIT, can bind multiple oncogene promoter G4s and downregulate their expression (Jagannath et al. Scientific Reports 2017, 7, 40706).Can authors provide evidence that NIT binds specifically to *MET*-G4?

   Response: Thank you for this insightful comment and for highlighting the relevant literature. We agree that natural benzophenanthridine alkaloids, including nitidine (NIT), commonly exhibit weak binding selectivity to a diverse type of G4 structures.

   To determine the binding selectivity of NIT for the parallel *MET*-G4 relative to other G4 topologies, we performed CD and fluorescence experiments. The data demonstrated that NIT exhibits strong binding affinity for diverse G4 structures, as evidenced by substantial thermal stabilization (ΔT_m up to 26 °C) and tight binding (K_d values in the low nanomolar range) across

parallel, antiparallel, and hybrid G4 topologies (Page18, line394-397, Supplementary Fig. 7, 8).

We have included a discussion about nitidine's selectivity on discussion part (Page 27, line 579-584). "Strikingly, although nitidine lacks intrinsic target selectivity, its biological activity becomes functionally selective in *MET*-amplified cancers: it preferentially binds to and stabilizes the LRPPRC-*MET*-G4 interaction complex in these cells, leading to coordinated downregulation of *MET* mRNA and protein levels. This G4-mediated mechanism underlies nitidine's selective cytotoxicity against *MET*-amplified cancer cells."

9. DMSO served as the solvent for the ligand solution. It is worth considering that the addition of DMSO could disrupt *MET*-G4 formation. It is not clear from the current results whether the author has taken this into consideration.

Response: Thanks for the comments. We investigated the impact of DMSO on *MET*-G4 structure using 1D NMR titration experiments (Page 18, line 383-385, Supplementary Fig. 6). As seen in the new Supplementary Fig. 6, DMSO only has a negligible effect on *MET*-G4 formation.

10. DNA polymerase stop assay demonstrated the ability of NIT to stabilize *MET*-G4 structure in long DNA contexts and thereby inhibit DNA replication. However, to confirm the cell-line specificity, the cytotoxicity of NIT to normal human cells should be tested.

Response: Thank you for raising this important point. We have now evaluated the cytotoxicity of nitidine (NIT) against two normal human liver cell lines (WRL68 and THLE-2) in comparison to the tested cancer cell lines. The results demonstrated that NIT exhibited higher IC_{50} values (indicating lower cytotoxicity) in both WRL68 and THLE-2 cells (Page21, lines 447-450, Supplementary Fig. 9h).

Reviewer #4 (Remarks to the Author):

The work by Ling-Yi Kong and group is an excellent research in G-quadruplex research in this specified domain. I am really very happy to review this paper. MET is an excellent target in several cancers. The promoter G quadruplex structure of it where the LRPPRC protein is binding is an hotspot to target the gene to repress its transcriptional fate. Nitidine as a molecular glue bridge the G-Quadruplex and the said protein. This cultivation is new in the field. This complex (MET-GQ-NITIDINE_LRPPRC) alters the structure and function of LRPPRC, ultimately leading to MET downregulation and tumor suppression. An array of high resolution NMR for G4 structure calculation, biophysical, in cellulo, and animal studies very well established that NITIDINE can be employed in hepatocellular carcinoma to target MET.
The work sounds very good and I recommend publication of the work in Nature Communication after revision.

Response: We sincerely thank you for your valuable support of our findings and for your insightful feedback.

Major points to be added and explained.

1. I recommend BG4 chip experiment in Hep-G2 cell to be performed to find out the expression of G4 in the promoter of MET gene.

Response: We sincerely appreciate the reviewer's insightful suggestion. To directly detect and map G4 structures within the *MET* promoter region in a cellular context, we performed the G4P CUT&Tag (Cleavage Under Targets and Tagmentation) assay in HepG2 cells. This assay offers higher sensitivity and resolution for profiling genomic G4 landscapes.

The G4P CUT&Tag data reveal robust, specific enrichment of G4 signals precisely within the *MET* promoter region in HepG2 cells, offering direct, genome-wide evidence that endogenous *MET*-G4 structures form in cells. These findings not only validate our prior biophysical assays and functional perturbations but also establish that the *MET* promoter adopts a stable, biologically relevant G4 conformation under native cellular conditions.

We have included the G4P CUT&Tag results in the revised manuscript (Page10, line220-222, Fig. 3b)

2. Please show affinities of Nitidine with other G-quadruplex structures as control. Find out the Kds using spectroscopic or calorimetric approach.

Response: We thank the reviewer for this valuable suggestion. To systematically assess nitidine's binding affinity and selectivity across diverse G4 topologies, we performed CD and fluorescence experiments. The data demonstrated that nitidine exhibits strong binding affinity for diverse G4 structures, as evidenced by substantial thermal stabilization (ΔT_m up to 26 °C) and tight binding (K_d values in the low nanomolar range) across parallel, antiparallel, and hybrid G4 topologies (Page18, line394-397, Supplementary Fig. 7, 8).

Response to Reviewers

The following are our point-by-point responses to reviewers' comments.

REVIEWER COMMENTS

Reviewer #1 (Remarks to the Author):

The revised manuscript failed to convincingly address several of my criticisms.

POINT N. 1

In my referee report on the first version of the manuscript, in the General Comments, I noted (a) the lack of a clear demonstration that interference with the LRPPRC–MET–G4 complex leads to actual MET protein downregulation in several experiments. Linked to this general observation, I raised, as major concern No. 1 related to Figure 3b, that: "Evidence for MET downregulation by LRPPRC silencing is weak. To corroborate this finding (a) an additional siRNA targeting LRPPRC should be tested to rule out off-target effects; (b) given that MET mRNA levels are reduced by only ~50%, and considering the complexity of transcriptional and translational regulation of MET, it is essential to demonstrate effects at the protein level; (c) experiments should be replicated in at least one additional cell line to confirm generalizability".

In response to my criticisms, the authors added experiments with an additional siRNA targeting LRPPRC in HepG2 (Extended Data 3d–e) and replicated the experiment in the HCCLM3 cell line. These experiments clearly demonstrate that, although LRPPRC was correctly downregulated in both cell lines together with MET mRNA, the final impact on MET protein is negligible (contrary to what is reported by the authors at lines 245–258). Please note that the MET band decreased in the western blot shown in Extended Data 3g is the upper MET band, corresponding to the inactive intracellular precursor, whereas the mature MET protein is only negligibly reduced. Moreover, the proliferation assay shown in Extended Data 4h is meaningless, as it was performed in the absence of HGF, which is required to activate MET and induce MET-dependent proliferation.

By performing these assays in the absence of HGF, the authors also failed to take into account my major point No. 2, which was as follows: "Fig. 3d. Based on the description, the proliferation assay appears to be conducted in the absence of HGF. Since HepG2 cells express wild-type MET, which is not constitutively active and requires HGF for activation, any observed anti-proliferative effect following LRPPRC silencing cannot be attributed to inhibition of MET signaling. Instead, it is more likely due to the multiple other functions of LRPPRC, which extend beyond MET regulation." Obviously, the requirement for HGF applies not only to HepG2 cells, but also to the other cell lines in which the experiments were replicated.

The results shown in the revised manuscript therefore argue against a prominent role of LRPPRC in regulating MET expression: the mRNA effect is irrelevant if it does not translate into an effect at the protein level.

Response:

Point 1.1: We sincerely thank the reviewer for the insightful comments and the important observation regarding the two MET bands detected by Western blot. As the antibody (CST, #8198S) recognizes both the immature pro-MET precursor (~170 kDa, upper band) and the mature, functionally active receptor (~140 kDa, lower band), our original description inadvertently conflated these distinct molecular forms. To directly address your concern, we have performed quantitative densitometry, restricted exclusively to the mature MET band (~140 kDa, lower band), across three independent biological replicates in both HepG2 and HCCLM3 cell lines (n = 3) (Reviewer Fig. 1). Our analysis reveals a statistically significant decrease in mature MET protein upon LRPPRC knockdown: in HepG2 cells, two distinct siRNAs against LRPPRC reduced mature MET levels by an average of 58% relative to siNC control; in HCCLM3 cells, the same intervention yielded a 52% reduction. We have added a note at the corresponding figure legend to explicitly indicate that the mature MET isoform is the lower band (~140 kDa). Critically, these results confirm that LRPPRC depletion leads not only to downregulation of *MET* mRNA, but also to a significant, reproducible reduction in functional MET protein. These data support our conclusion that LRPPRC plays a prominent role in regulating *MET* expression.

Moreover, depletion of LRPPRC leads to a moderate (~50%) downregulation of both *MET* mRNA and protein levels, which is reasonable. While the present study centers on the LRPPRC–*MET*-G4 interaction, G4-based pull-down and siRNA-mediated knockdown experiments identified several additional *MET*-G4-binding proteins, including EIF3A, DHX9, and HADHA, all of which are implicated in *MET* signaling regulation. Therefore, *MET*-G4–mediated transcriptional upregulation of the *MET* oncogene is likely orchestrated by a cooperative network of these proteins. LRPPRC knockdown reduces *MET* expression, yet the remaining G4-binding proteins (e.g., EIF3A, DHX9, HADHA) may still engage *MET*-G4 and partially sustain *MET* signaling activity. Notably, the natural compound nitidine functions as a molecular glue that effectively stabilizes the LRPPRC–*MET*-G4 interaction complex. This stabilization sequesters LRPPRC on *MET*-G4, likely impairing its native regulatory functions. Moreover, the formed stable ternary complex may sterically hinder the binding of other *MET*-G4 interactors, including EIF3A, DHX9, and HADHA, thereby inducing sustained suppression of *MET* signaling. Our work thus reveals a previously uncharacterized anticancer mechanism: stabilization of pathogenic G4-protein interfaces via molecular glues to kill cancer cells. We propose that developing potent, selective molecular glues targeting the LRPPRC–*MET*-G4 interface represents a promising therapeutic strategy, one with broader implications for G4-protein-targeted drug discovery in oncology. We hope this explanation can address your concerns.

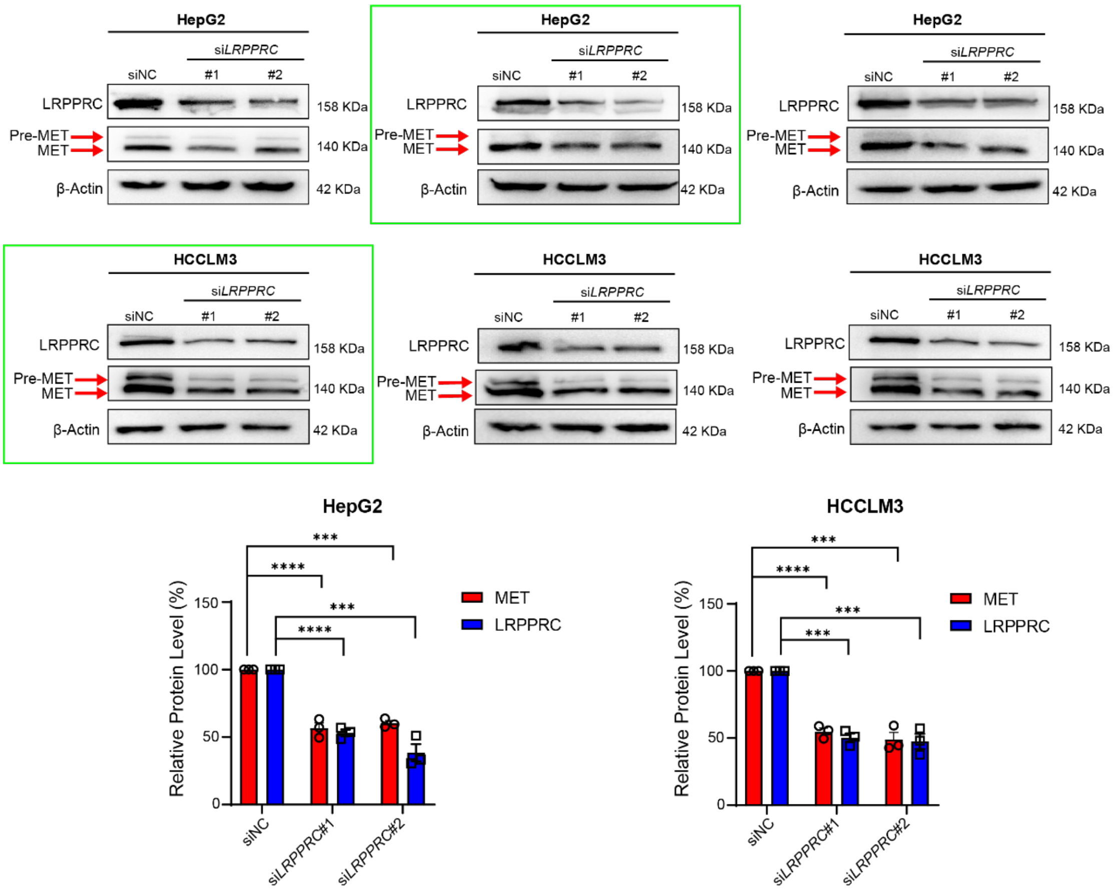

Reviewer Fig. 1. MET protein expression levels in HepG2 or HCCLM3 cells with or without LRPPRC knockdown. Quantification of the mature MET protein (lower band) and LRPPRC from three independent experimental replicates (n=3) is shown as means ± S.E.M. Quantification was normalized to β-Actin bands, and values are presented relative to the siNC group. Statistical analysis was performed using a two-tailed t-test. ****p < 0.0001, ***p < 0.001, **p < 0.01. The images enclosed in green boxes were selected as representative figures for the manuscript.

Point 1.2: We fully agree with the reviewer's suggestion to assess *MET*-dependent proliferation under HGF-stimulated conditions. To address this point, we repeated the proliferation assay in HCCLM3 cells following stimulation with recombinant human HGF. As shown in the updated Extended Data Figures 4e, 4h, and 9b, and Supplementary Figure 9f, HGF-induced proliferation was significantly suppressed in both MET/LRPPRC-knockdown cells and nitidine-treated cells.

POINT . 2

In my referee report on the first version of the manuscript, General Comments point (c), I objected that the use of nitidine required appropriate controls, as it is already known that this compound exerts antitumor effects through various mechanisms (see, for example, PMID: 33024576). However, the results lacked appropriate controls to exclude MET-independent nitidine effects,

particularly in the in vivo experiments (e.g., by using tumor cells lacking MET expression).

In their reply, the authors provide an answer that fails to address this point. First, they state that they conducted experiments in "*HepG2 cells, a model of hyperactivated MET signalling*". This definition does not apply to HepG2, which expresses (as is well known from the literature and as shown by the authors in Extended Data Fig. 2) only modest levels of MET. Second, based on the new experiments added (Extended Data Fig. 9a–b), they conclude (in the reply) that "*nitidine's antiproliferative activity stems not only from MET signaling inhibition but also from complementary, MET-independent pathways, aligning with its established polypharmacological profile*". Thus, the new experiments confirm my objection: nitidine is a non-specific compound. When administered to tumors, it is impossible to discriminate MET-mediated effects from other activities. This is a serious confounding factor that precludes rigorous preclinical—and, even more so, clinical—testing of the molecule as a novel anti-MET agent. Considering the lack of evidence that the nitidine target LRPPRC can meaningfully affect MET protein levels, and given that nitidine has numerous effects, one must also consider the possibility that the reduction of MET protein levels shown in Extended Data Fig. 11 results from mechanisms independent of stabilization of the LRPPRC–MET–G4 interaction.

In their reply to my comment on nitidine's lack of specificity, the authors observe that "*capmatinib, a clinically approved MET inhibitor that primarily inhibits phosphorylation of the MET oncoprotein, failed to show a clear concentration-dependent reduction in total MET expression*". It must be stressed that no protein downregulation is expected from tyrosine kinase inhibitors; this is neither expected nor required for clinical effectiveness. The authors then conclude that "*mechanistically, although nitidine lacks intrinsic target selectivity, its biological activity becomes functionally selective in MET-amplified cancers: it preferentially binds to and stabilizes the LRPPRC–MET–G4 interaction complex in these cells, leading to coordinate downregulation of MET mRNA and protein levels. This G4-mediated mechanism underlies nitidine's selective cytotoxicity against MET-amplified cancer cells*". These claims—which are particularly relevant for clinical translation and therefore should be expressed with extreme caution—lack any supporting evidence.

In summary, this manuscript fails to show that LRPPRC protein, the proposed nitidine target, is responsible for any significant modulation of MET protein levels, and it provides evidence that nitidine can exert antitumor effects independently of MET targeting. At this stage, the biological evidence does not provide any proof of concept that nitidine can be proposed as a new strategy for MET inhibition with clinical translation potential. If the manuscript is accepted for publication based on positive comments by the other reviewers, claims contradicted by the experimental results must be removed from the manuscript, and an honest, rigorous critical assessment of the study limitations and of the translational potential of nitidine must be added.

Response:

Point 2.1: We apologize for the inaccurate description of "HepG2 cells, a model of hyperactivated MET signalling" in the manuscript, which is a mistake. HepG2 cells are not inherently hyperactivated for *MET* signalling; rather, they serve as a responsive model in which *MET* signalling can be robustly activated upon HGF stimulation. This wording has now been corrected on page 20, line 435.

"we employed HGF-stimulated HepG2 cells, a model with an active MET signaling pathway."

Point 2.2: We assessed MET expression levels following capmatinib treatment to clarify that capmatinib and nitidine inhibit the MET signaling pathway through distinct mechanisms. The relevant text has been revised to improve clarity and scientific precision. Page 21, line 455-463.

"As a result, nitidine significantly suppressed both MET mRNA and protein levels in a concentration-dependent manner (Supplementary Fig. 10a, b and Extended Data Fig. 11b, c), whereas capmatinib, a clinically approved MET tyrosine kinase inhibitor[44], failed to reduce MET expression levels consistently across doses or cell lines (Supplementary Fig. 10c-f). This contrast aligns with their distinct mechanisms: capmatinib selectively inhibits MET phosphorylation and downstream signaling without affecting total MET abundance, while nitidine mainly suppresses MET transcription, thereby reducing overall MET expression."

Point 2.3: We acknowledge that nitidine likely engages multiple targets, which complicates mechanistic interpretation. Nevertheless, our integrated biophysical, genetic, and pharmacological analyses robustly demonstrate that nitidine effectively stabilizes the LRPPRC–*MET*-G4 interaction complex, a previously unrecognized anticancer mechanism with compelling therapeutic potential. This work establishes the LRPPRC–*MET*-G4 interaction complex as a biologically significant and druggable target. Nitidine thus serves not merely as a bioactive compound but as a chemical probe that validates this novel, targetable node in cancer biology. We have included a discussion about the specificity problem in the discussion part. We deleted the claims of "*mechanistically, although nitidine lacks intrinsic target selectivity, its biological activity becomes functionally selective in MET-amplified cancers: it preferentially binds to and stabilizes the LRPPRC-MET-G4 interaction complex in these cells, leading to coordinate downregulation of MET mRNA and protein levels. This G4-mediated mechanism underlies nitidine's selective cytotoxicity against MET-amplified cancer cells*" in the main text.

Point 2.4: We have thoroughly revised the manuscript to ensure all claims are fully supported by the experimental results. In the Discussion section, we now provide an honest, rigorous critical assessment of the study limitations. Page 27-29, line 583-621.

"Beyond these novel findings, this study has several limitations that warrant careful consideration. First, our work primarily focuses on the LRPPRC–MET-G4 interaction, whereas G4-based pull-down and siRNA-mediated knockdown experiments identified additional MET-G4–binding proteins, including EIF3A, DHX9, and HADHA, all implicated in MET signaling regulation (Fig. 6f) . The functional interplay among these proteins (e.g., cooperative or competitive binding, hierarchical assembly, or allosteric crosstalk), their collective influence on MET transcription, and how nitidine modulates this regulatory network remain unclear. Addressing these questions is essential to map the broader landscape of MET-G4-mediated gene control. Second, although nitidine robustly stabilizes the LRPPRC–MET-G4 complex across multiple MET-dependent cancer cell lines, resulting in coordinated downregulation of both MET mRNA and protein, it lacks sufficient selectivity to function as a "pure" molecular glue specific to this interface. Its polypharmacological profile complicates mechanistic interpretation[61], as off-target contributions cannot be excluded. Nevertheless, nitidine's preferential cytotoxicity in MET-addicted cancer cells strongly supports the biological relevance and therapeutic potential of pharmacologically targeting the LRPPRC–MET-G4 interface. Importantly, nitidine is not intended as a clinical candidate; rather, it serves as a chemical probe to establish this previously unexplored, targetable node. Third, high-resolution structures of the ternary LRPPRC–NIT–MET-G4 and binary MET-G4–NIT complexes remain undetermined. NMR titration of nitidine into MET-G4 induced pronounced broadening and substantial chemical shift perturbations in the imino proton signals (Extended Data Fig. 8c), indicating dynamic, multi-modal binding with rapid-to-medium exchange among several distinct conformations, rendering the high-resolution structural determination of the complexes currently infeasible. Notably, structurally related natural alkaloids, berberine and coptisine, have shown specific binding to parallel G4s, including KRAS-G4, MYC-G4, and PDGFR-β-G4[62-64]. Given their shared isoquinoline scaffold and cationic planar core, nitidine is predicted to bind MET-G4 primarily via π-π stacking and electrostatic interactions at the 5'- and 3'-terminal G-tetrads. However, its unique ring-fused pattern and extended aromatic conjugation system enhance overall binding affinity, yet at the cost of reduced specificity for parallel G4s, including MET-G4, compared to berberine and coptisine (Supplementary Fig. 12). To advance therapeutic targeting of LRPPRC–MET-G4 interaction, future efforts should prioritize two key directions: (i) high-resolution structural characterization of the Ligand–MET-G4 and LRPPRC–Ligand–MET-G4 complexes using integrated NMR, X-ray, and cryo-EM approaches; and (ii) rational design of next-generation molecular glues targeting the LRPPRC–MET-G4 interaction complex, achieved either through scaffold hopping from the isoquinoline core or via strategic side-chain derivatization, to simultaneously improve potency, selectivity, and drug-like properties."

We really thank the reviewer for these rigorous and constructive critiques throughout this review process. These comments have significantly improved the clarity, honesty, and scientific rigor of our manuscript. We believe the revised version now presents a balanced interpretation of our findings, appropriately acknowledges the limitations, and accurately reflects the strength of the evidence. We hope that with these revisions, the manuscript is now suitable for publication.

Reviewer #2 (Remarks to the Author):

All of my question/comments were addressed.

Response: We sincerely thank you for your valuable support of our findings and for your insightful feedback.

Reviewer #3 (Remarks to the Author):

I have reviewed the revised version of the manuscript submitted for publication in Nature Communications. All changes were clearly tracked, and I compared this version with the previous one. It is an impressive work and the revised manuscript has been improved. I recommend the manuscript for publication, however, only after minor corrections.

The regulatory mechanism at the MET promoter inferred based on the RT-qPCR experimental results can be incorporated into the main text, which will facilitate the understanding of the G4-based regulation.

NIT has been thoroughly analyzed by biophysical, genetic, and pharmacological experiments and confirmed to have excellent binding ability for stabilizing the LRPPRC-*MET*-G4 complex. Unfortunately, the structure of the ternary *MET*-G4-NIT-LRPPRC complex could not be obtained through NOESY data. Any comparative analysis of NIT's binding behavior with its analogues, such as berberine, should be incorporated to discuss how to enhance the NIT's selective G4-based antitumor effects.

Response: We sincerely thank the reviewer for the thoughtful evaluation of our revised manuscript and for recommending it for publication. We have moved the relevant RT-qPCR data from the Supplementary Information into Extended Data Fig. 11, integrating it directly with the mechanistic findings presented in the main text. We have included a discussion about the binding model of nitidine to *MET*-G4 and provided suggestions to enhance its selective G4-based antitumor effects in the discussion part. Page 28-29, line 600-621.

"Third, high-resolution structures of the ternary LRPPRC–NIT–MET-G4 and binary MET-G4–NIT complexes remain undetermined. NMR titration of nitidine into MET-G4 induced pronounced broadening and substantial chemical shift perturbations in the imino proton signals (Extended Data Fig. 8c), indicating dynamic, multi-modal binding with rapid-to-medium exchange among several distinct conformations, rendering the high-resolution structural determination of the complexes currently infeasible. Notably, structurally related natural alkaloids, berberine and coptisine, have shown specific binding to parallel G4s, including KRAS-G4, MYC-G4, and PDGFR-β-G4[62-64]. Given their shared isoquinoline scaffold and cationic planar core, nitidine is predicted to bind MET-G4 primarily via π–π stacking and electrostatic interactions at the 5'- and 3'-terminal G-tetrads. However, its unique ring-fused pattern and extended aromatic conjugation system enhance overall binding affinity, yet at the cost of reduced specificity for parallel G4s, including MET-G4, compared to berberine and coptisine (Supplementary Fig. 12). To advance therapeutic targeting of LRPPRC–MET-G4 interaction, future efforts should prioritize two key directions: (i) high-resolution structural characterization of the Ligand–MET-G4 and LRPPRC–Ligand–MET-G4 complexes using integrated NMR, X-ray, and cryo-EM approaches; and (ii) rational design of next-generation molecular glues targeting the LRPPRC–MET-G4 interaction complex, achieved either through scaffold hopping from the isoquinoline core or via strategic side-chain derivatization, to simultaneously improve potency, selectivity, and drug-like properties."

We thank the reviewer once again for your thoughtful comments and for their positive assessment of our work.

Reviewer #4 (Remarks to the Author):

The work entitled "Molecule Glue That Stabilizes the LRPPRC−*MET*-G4 Interaction Complex to Drive MET Downregulation" has been well revised.
The authors have satisfactorily answered all the questions in the revision.
I strongly recommend the publication of the article.

Response: We sincerely thank you for your valuable support of our findings and for your insightful feedback.

Reviewer #3 (Remarks to the Author):

I have reviewed the revised version of the manuscript submitted for publication in Nature Communications. All changes were clearly tracked, and I compared this version with the previous one. It is an impressive work and the revised manuscript has been improved. I recommend the manuscript for publication, however, only after minor corrections.

The regulatory mechanism at the MET promoter inferred based on the RT-qPCR experimental results can be incorporated into the main text, which will facilitate the understanding of the G4-based regulation.

NIT has been thoroughly analyzed by biophysical, genetic, and pharmacological experiments and confirmed to have excellent binding ability for stabilizing the LRPPRC-*MET*-G4 complex. Unfortunately, the structure of the ternary *MET*-G4-NIT-LRPPRC complex could not be obtained through NOESY data. Any comparative analysis of NIT's binding behavior with its analogues, such as berberine, should be incorporated to discuss how to enhance the NIT's selective G4-based antitumor effects.

Response: We sincerely thank the reviewer for the thoughtful evaluation of our revised manuscript and for recommending it for publication. We have moved the relevant RT-qPCR data from the Supplementary Information into Extended Data Fig. 11, integrating it directly with the mechanistic findings presented in the main text. We have included a discussion about the binding model of nitidine to *MET*-G4 and provided suggestions to enhance its selective G4-based antitumor effects in the discussion part. Page 28-29, line 600-621.

"Third, high-resolution structures of the ternary LRPPRC–NIT–MET-G4 and binary MET-G4–NIT complexes remain undetermined. NMR titration of nitidine into MET-G4 induced pronounced broadening and substantial chemical shift perturbations in the imino proton signals (Extended Data Fig. 8c), indicating dynamic, multi-modal binding with rapid-to-medium exchange among several distinct conformations, rendering the high-resolution structural determination of the complexes currently infeasible. Notably, structurally related natural alkaloids, berberine and coptisine, have shown specific binding to parallel G4s, including KRAS-G4, MYC-G4, and PDGFR-β-G4[62-64]*. Given their shared isoquinoline scaffold and cationic planar core, nitidine is predicted to bind MET-G4 primarily via π–π stacking and electrostatic interactions at the 5'- and 3'-terminal G-tetrads. However, its unique ring-fused pattern and extended aromatic conjugation system enhance overall binding affinity, yet at the cost of reduced specificity for parallel G4s, including MET-G4, compared to berberine and coptisine (Supplementary Fig. 12). To advance therapeutic targeting of LRPPRC–MET-G4 interaction, future efforts should prioritize two key directions: (i) high-resolution structural characterization of the Ligand–MET-G4 and LRPPRC–Ligand–MET-G4 complexes using integrated NMR, X-ray, and cryo-EM approaches; and (ii) rational design of next-*

generation molecular glues targeting the LRPPRC–MET-G4 interaction complex, achieved either through scaffold hopping from the isoquinoline core or via strategic side-chain derivatization, to simultaneously improve potency, selectivity, and drug-like properties."

We thank the reviewer once again for your thoughtful comments and for their positive assessment of our work.

Reviewer #4 (Remarks to the Author):

The work entitled "Molecule Glue That Stabilizes the LRPPRC−*MET*-G4 Interaction Complex to Drive MET Downregulation" has been well revised.
The authors have satisfactorily answered all the questions in the revision.
I strongly recommend the publication of the article.

Response: We sincerely thank you for your valuable support of our findings and for your insightful feedback.

Response to Third Round Reviewers' Comments

Reviewer #1 (Remarks to the Author):

The experiments presented in the new extended Fig. 9a,b and in Supplementary Fig. 9f still lack an important control: a comparison of the effects of nitidine in the presence and absence of HGF. Indeed, my request was obviously not to replace the experiment performed in the absence of HGF with one performed in the presence of HGF, but rather to compare the effect of nitidine under both conditions, in order to assess the relative contribution of nitidine to the inhibition of HGF-dependent proliferative effects, as opposed to more general inhibitory effects.

Therefore, the issue of nitidine's MET-unrelated effects remains unresolved.

However, given the enthusiasm of the other reviewers and the more honest analysis of the study's limitations, the manuscript deserves publication without further experimental revision.

I strongly recommend rendering the abstract more balanced by:

1. Changing the statement "This complex likely alters the structure and function of LRPPRC, ultimately leading to MET downregulation and potent anticancer effects" (lines 29–30) to: "This complex likely alters the structure and function of LRPPRC, ultimately leading to MET downregulation and anticancer effects that add to those already reported for nitidine."

2. Deleting the word "mainly" (line 33) from the statement: "Additionally, comprehensive in vitro and in vivo experiments demonstrated that nitidine significantly inhibits tumor progression mainly through an LRPPRC−MET-G4-dependent mechanism."

Response:

We sincerely thank the reviewer for the constructive feedback and for recognizing the strengths of our study. In response to the reviewer's remaining concerns, we performed CCK-8 assays to assess nitidine's effect on cell viability under two conditions: with versus without HGF stimulation (Supplementary Fig. 20a). These experiments were conducted in parallel in both HepG2 and HCCLM3 cell lines. As expected, nitidine exerted significantly stronger inhibition of cell viability in HGF-stimulated cells compared to unstimulated controls. This newly collected data has been integrated into the main text (page 20, lines 434).

Moreover, we have revised the abstract as suggested. We believe the abstract now more accurately reflects the findings.

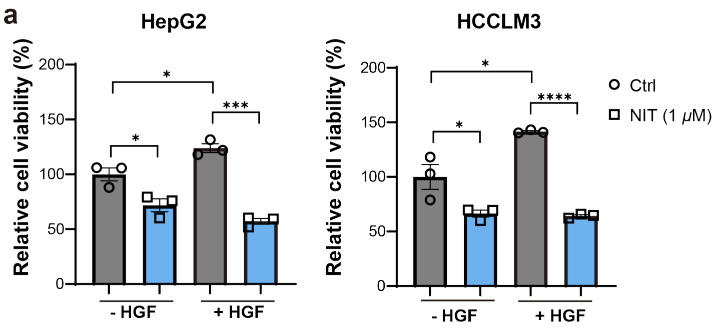

Reviewer Fig. 1. Cell proliferation was assessed using the CCK-8 assay in 40 ng/mL HGF-stimulated HepG2 and HCCLM3 cells treated with 1 μM nitidine for 24h. Data are presented as mean ± SEM; n = 3, **p* = 0.028, **p* = 0.029, ****p* = 0.0001 (for HepG2), **p* = 0.047, **p* = 0.022, *****p* = 0.0000006 (for HCCLM3), two-tailed t-test.

Reviewer #3 (Remarks to the Author):

All comments have been addressed. I recommend the manuscript for publication.

Response:

We thank the reviewer for the positive evaluation and for recommending the manuscript for publication.
